# Synthetic cannabinoid receptor agonists containing silicon: exploring the metabolic pathways of ADMB- and Cumyl-3TMS-PrINACA in human urine specimens and post mortem material compared to in vitro and in silico data

**DOI:** 10.1007/s00204-025-04204-y

**Published:** 2025-10-07

**Authors:** Annette Zschiesche, Jeremy Carlier, Jörg Pietsch, Martin Scheu, Jasmin Seibt, Francesco P. Busardò, Volker Auwärter, Laura M. Huppertz

**Affiliations:** 1 Institute of Forensic Medicine, Forensic Toxicology, Medical Center and Faculty of Medicine, University of Freiburg, Albertstr. 9, 79104 Freiburg, Germany; 2 Hermann Staudinger Graduate School, University of Freiburg, Hebelstr. 27, 79104 Freiburg, Germany; 3 Section of Legal Medicine, Department of Biomedical Sciences and Public Health, Marche Polytechnic University, Via Tronto 10/a, 60126 Ancona, Italy; 4 Institute of Legal Medicine, Medical Faculty Carl Gustav Carus, Dresden Technical University, Fetscherstr. 74, 01307 Dresden, Germany

**Keywords:** New psychoactive substances, Synthetic cannabinoids, 3-Trimethylsilyl propyl tail (3TMS-moiety), LC-HRMS/MS, Metabolism, In silico metabolite prediction

## Abstract

The rapid emergence of synthetic cannabinoid receptor agonists (SCRAs) poses challenges for drug testing, particularly when analyzing urine samples due to the rapid metabolization of the parent compounds. In early 2023, two novel SCRAs were reported to the European Union Drugs Agency (EUDA): ADMB-3TMS-PrINACA and Cumyl-3TMS-PrINACA, which are both indazole SCRAs featuring a trimethylsilyl propyl moiety connected to the tertiary indazole nitrogen. Peaks corresponding to metabolites of ADMB-BINACA (also known as ADB-BUTINACA) and Cumyl-4CN-BINACA observed with retention time shifts in a liquid chromatography-tandem mass spectrometry (LC-MS/MS) method for detecting SCRAs were later identified as metabolites of ADMB- and Cumyl-3TMS-PrINACA. Pooled human liver microsome (pHLMs, 25 µmol/L) and pooled human hepatocyte (PHH, 20 µmol/L) assays were performed to generate metabolites. Additionally, human urine samples were analyzed by reversed phase liquid chromatography-quadrupole-time-of-flight-mass spectrometry (LC-QToF-MS), assisted by GLORYx and BioTransformer 3.0 for in silico metabolite prediction. Gas chromatography-mass spectrometry (GC-MS) was used to identify substances in seized materials. In total, 34 metabolites for ADMB-3TMS-PrINACA and 38 for Cumyl-3TMS-PrINACA were tentatively identified. Major biotransformations included side chain monohydroxylation (specific markers) and TMS-group cleavage, likely initiated by oxidative Si-demethylation followed by further hydroxylation resulting in an *N*-3-OH-propyl metabolite and further oxidation to the respective *N*-propionic acid. Most of these biomarkers were detected in the blood, urine, and stomach content of a deceased poly-drug user exposed to ADMB-3TMS-PrINACA. Overall, Cumyl-3TMS-PrINACA was more prevalent than ADMB-3TMS-PrINACA in Germany according to routine urine testing. This work provides the first investigation of the metabolic fate and suggests biomarkers for these new SCRAs.

**Supplementary Information:**

The online version contains supplementary material available at 10.1007/s00204-025-04204-y.

## Introduction

Biotransformation studies of synthetic cannabinoid receptor agonists, formerly known as “Spice”, are crucial for abstinence control in forensic toxicology. Due to the extensive metabolism of SCRAs, unlike in blood, hair, or saliva, the parent compounds are often not detectable in urine (Diao and Huestis 2019; Giorgetti et al. 2024; Navarro-Tapia et al. 2022). Therefore, it is inevitable to investigate the major biotransformation products excreted after the ingestion of the respective SCRA prior to developing methods for urine analysis.

SCRAs are typically consumed to mimic the effects of the phytocannabinoid Δ9-tetrahydrocannabinol (THC), a partial agonist at the human cannabinoid receptor 1 (hCB_1_). In contrast, most SCRAs act as full agonists at the same receptor. Hence, the side effects (e.g., seizures and coma) associated with their consumption are often more severe (Cohen and Weinstein 2018; Tai and Fantegrossi 2014; van Amsterdam et al. 2015). Particularly in forensic psychiatric facilities and prisons, the circulation of papers and herbal blends impregnated with SCRAs intended for smoking is common and poses significant health risks (Abbott et al. 2023; Cozier et al. 2023; Norman et al. 2020).

The continuous emergence of new SCRAs to evade regulations is often described as a “cat-and-mouse game” between legislators and manufacturers. As the largest new psychoactive substance (NPS) subset, SCRAs impose a substantial workload on forensic toxicology laboratories striving to keep pace with the rapidly evolving drug market, also creating significant challenges for law enforcement authorities. This was exemplified by the appearance of two SCRAs carrying a 3-trimethylsilylpropyl tail at the tertiary indazole nitrogen: ADMB-3TMS-PrINACA and Cumyl-3TMS-PrINACA.

ADMB-3TMS-PrINACA, also known as ADMB-3TMS-PRINACA, ADB-3TMS-PrINACA, or 3TMS-ADB-PRINACA (*N*-(1-amino-3,3-dimethyl-1-oxobutan-2-yl)-1-(3-(trimethylsilyl)propyl)-1*H*-indazole-3-carboxamide), appeared on the German drug market in early March 2023. It resembles ADMB-FUBINACA (ADB-FUBINACA), with the fluorobenzyl tail (FUB) replaced by a 3-trimethylsilylpropyl tail (3TMS-Pr). It features an indazole core (INA), a carboxamide linker (CA), and an amino dimethyl butanone (ADMB) moiety. This compound is the first reported NPS containing a silicon atom in its structure (EUDA 2025a).

Cumyl-3TMS-PrINACA, also known as Cumyl-3TMS-PRINACA or 3TMS-CUMYL-PRINACA (*N*-(2-phenylpropan-2-yl)-1-(3-(trimethylsilyl)propyl)-1*H*-indazole-3-carboxamide), differs from ADMB-3TMS-PrINACA only by featuring a cumyl instead of the ADMB moiety. As of July 2025, Cumyl-3TMS-PrINACA has been reported in seizures in Germany, Sweden, and Hungary (EUDA 2025b).

**Fig. 1:**
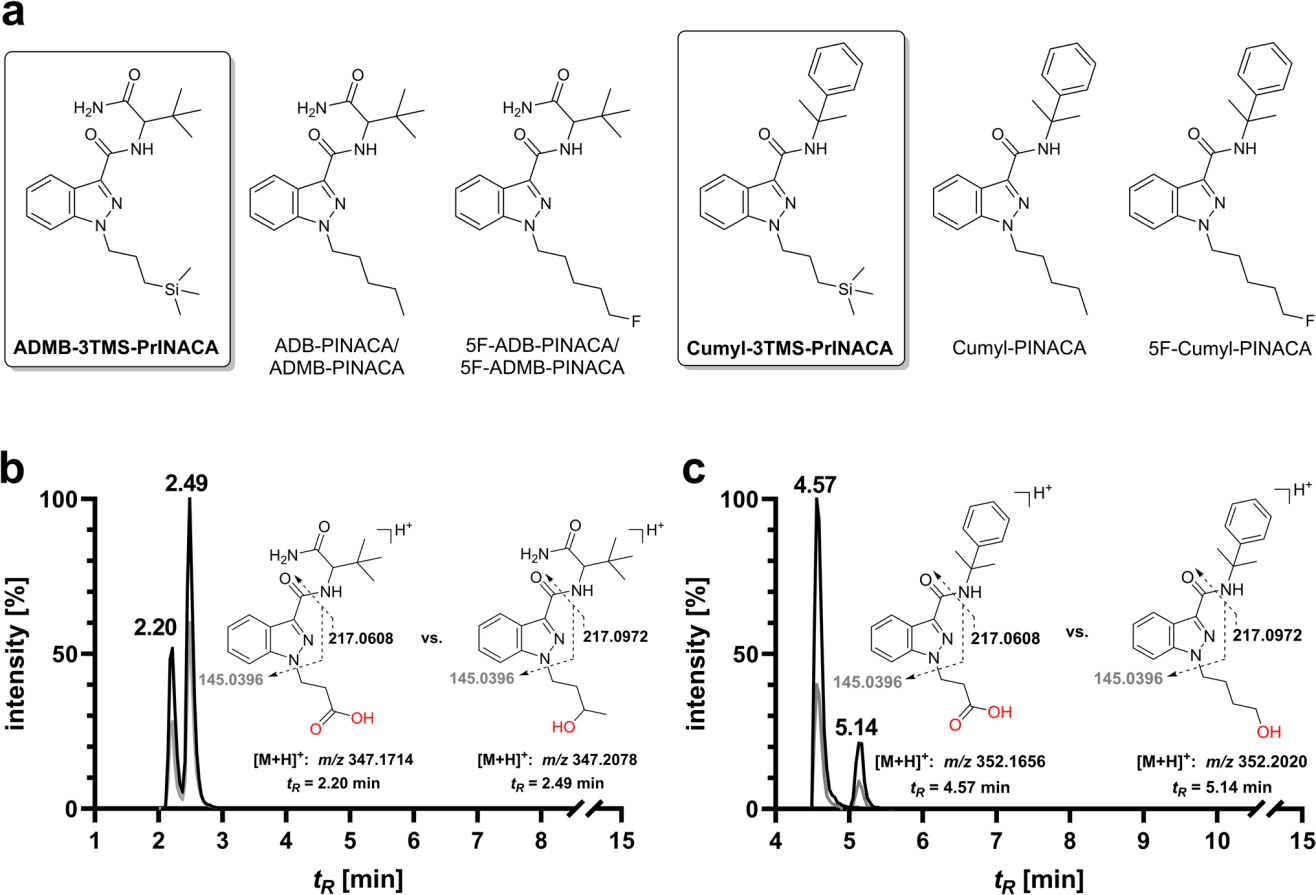
**a** ADMB-3TMS-PrINACA (left) and Cumyl-3TMS-PrINACA (right) and their most closely structurally related SCRAs: ADB-PINACA, 5F-ADB-PINACA, Cumyl-PINACA and 5F-Cumyl-PINACA; **b** extracted ion chromatograms of the *N*-propionic acid metabolite of ADMB-3TMS-PrINACA (left) with the isobaric compound ADB-BUTINACA *N*-3-OH-butyl (right); **c** extracted ion chromatograms of the *N*-propionic acid metabolite of Cumyl-3TMS-PrINACA (left) with the isobaric analog Cumyl-4CN-BINACA *N*-4-OH-butyl (right) found in LC-MS/MS (sMRM) experiments by retention time shifts. The first transition is shown in black, whereas the second in gray

At the end of 2022, the authors noticed a retention time shift in their routine targeted liquid chromatography-tandem mass spectrometry (LC-MS/MS) method for detecting SCRAs in urine and blood samples. The shift was observed for peaks corresponding to the transitions (*m*/*z* 347.2 → 217.1 and 347.2 → 145.0) of the *N*-3-OH metabolite of ADB-BUTINACA (*N*-(1-amino-3,3-dimethyl-1-oxobutan-2-yl)-1-butyl-1*H*-indazole-3-carboxamide, also known as ADB-BINACA or ADMB-BINACA). This metabolite has been described in several studies on ADB-BUTINACA metabolism (Sia et al. 2021; Kavanagh et al. 2022). Similarly, a metabolite of Cumyl-3TMS-PrINACA exhibited the same two transitions (*m*/*z* 352.2 → 217.1 and 352.2 → 145.0) as a biomarker hydroxylated at the *N*-4-OH butyl chain of Cumyl-4CN-BINACA (1-(4-cyanobutyl)-*N*-(1-methyl-1-phenyl–ethyl)indazole-3-carboxamide). The formation of this metabolite has also been reported in experimental studies (Åstrand et al. 2018; Öztürk et al. 2018). LC-QToF-MS analysis identified metabolites of ADMB-3TMS-PrINACA and Cumyl-3TMS-PrINACA characterized by the formal cleavage of the trimethyl silyl group, followed by multiple oxidation steps to a tentatively identified* N*-propionic acid metabolite. The structures of these isobaric metabolites are depicted in Fig. 1.

The substitution of carbon by silicon, known as a carbon-silicon switch or “sila-substitution”, is used in medicinal chemistry to study the effects of this switch on the molecular properties of the resulting compounds, and has particularly been used in drug design (Fotie et al. 2023; Tacke and Dörrich 2016). This approach has also been applied to (synthetic) cannabinoids, affecting pharmacological potency, pharmacokinetics, pharmacodynamics, and metabolic stability (Duan et al. 2021; Panayides et al. 2024). However, the primary purpose for synthesizing the silicon-containing cannabinoids described here was likely circumvention of legal regulation. Similarly, a trimethylsilyl derivative of 1P-LSD, called 1S-LSD (1-(3-(trimethylsilyl)propionyl) lysergic acid diethylamide), recently appeared online and is considered a legal LSD analog with similar effects (Open-Mind Market 2024; Tanaka et al. 2025). This trend seems to extend to other NPS classes as another strategy to evade legal restrictions.

To date, synthetic cannabinoids containing a silicon atom, an *N*-propyl side chain, or a trimethylsilyl propyl moiety have not been reported. This study aimed to fill the gap in knowledge regarding the in vitro and human in vivo metabolic pathways of the newly emerged synthetic cannabinoids ADMB-3TMS-PrINACA and Cumyl-3TMS-PrINACA. In silico metabolite prediction models were used to assess their accuracy when reference substances are unavailable for in vitro testing. Additionally, herbal blends and ash residue samples from individuals documented to have used one of these 3TMS-SCRA substances were analyzed by GC-MS to confirm the substances consumed.

## Materials and methods

### Chemicals and reagents

Formic acid (Rotipuran^®^ ≥ 98%, p.a.), sodium hydroxide (≥ 99%, p.a.), and methanol were obtained from Carl Roth (Karlsruhe, Germany). Acetonitrile (ACN, LC-MS grade, Optigrade^®^) was sourced from Promochem (Wesel, Germany), ammonium formate (10 mol/L, 99.995%) from Sigma-Aldrich (Steinheim, Germany), and isopropanol (Prepsolv^®^) from Merck (Darmstadt, Germany). Deionized water was prepared using an ELGA Medica^®^ Pro system (Celle, Germany).

Reference standards of ADMB-3TMS-PrINACA, Cumyl-3TMS-PrINACA, 5F-Cumyl-PINACA, ADB-INACA, ADB-PINACA, and 5F-ADB-PINACA were purchased from Cayman Chemical (Ann Arbor, MI, USA). Diclofenac was provided by Sigma Aldrich (Milan, Italy). The Slovenian National Forensic Laboratory in Ljubljana supplied Cumyl-PINACA.

Pooled human liver microsomes (pHLMs, 150 donors, 20 mg/mL protein in 250 mM sucrose), NADPH-regenerating solutions A and B, and potassium phosphate buffer 0.5 mol/L (pH 7.4) were from Corning (Amsterdam, the Netherlands). Cryopreserved pooled human hepatocytes (10 donors) and trypan blue (0.4%) were purchased from Lonza (Basel, Switzerland). Williams’ Medium E, supplemented with 2 mmol/L l-glutamine and 20 mol/L HEPES, as well as individual components, was from Sigma-Aldrich (Milan, Italy). Roche Diagnostics (Mannheim, Germany) supplied the β-glucuronidase (Escherichia coli K12) used for conjugate cleavage.

Blank urine was donated by two volunteers and tested for the absence of SCRAs and their metabolites prior to use.

Mobile phase A (1% ACN, 0.1% HCOOH, and 2 mmol/L HCOONH_4_ in water) and mobile phase B (0.1% HCOOH and 2 mmol/L HCOONH_4_ in ACN) were freshly prepared prior to analysis. Sodium formate cluster solution used for external and internal mass calibration of the QToF-MS instrument consisted of 500 mL deionized water, 500 mL isopropanol, 2 mL formic acid, and 10 mL sodium hydroxide (1 mol/L).

### Pooled human liver microsomes (pHLM) incubations

For phase I metabolite identification and plausibility, pHLM assays were performed by incubating ADMB-3TMS-PrINACA and Cumyl-3TMS-PrINACA separately (final concentration each: 10 µg/mL) in triplicates with control samples as stated elsewhere (Zschiesche et al. 2024).

The same protocol but with single incubation times of 30 min and 60 min was used for Cumyl-PINACA, 5F-Cumyl-PINACA, ADB-PINACA, and 5F-ADB-PINACA.

For identification of tentative metabolites by LC-HESI-QToF-MS analysis, 30 µL of the supernatant was evaporated to dryness under a stream of nitrogen and reconstituted in 30 μL mobile phase A/B (60/40, *v*/*v*).

### Pooled human hepatocyte (PHH) incubations

ADMB- and Cumyl-3TMS-PrINACA were incubated separately with 10-donor cryopreserved hepatocytes following a modified protocol (Di Trana et al. 2021). Thawed hepatocytes were washed, resuspended in supplemented Williams' Medium E (sWME), and adjusted to 2 × 10^6^ viable cells/mL (assessed by trypan blue exclusion). Incubations (250 μL cell suspension + 250 μL 20 μmol/L 3TMS-SCRA in sWME) were performed in sterile 24-well plates at 37 °C for 0 or 3 h. Negative (cells only), substances (compounds only), and positive (20 μmol/L diclofenac) controls were included. Reactions were quenched with 500 μL ice-cold ACN, centrifuged (15,000×*g*, 10 min, room temperature), and supernatants were stored at − 80 °C until analysis.

After thawing, the incubations were centrifuged for 10 min at 16,550×*g*. An aliquot of 100 μL of the supernatant was mixed with 100 μL of ACN and 50 µL of ammonia formate and centrifuged (10 min, 2898×*g*). The supernatant was evaporated to dryness under nitrogen at 40 °C, and the remaining residue was reconstituted in 100 μL of mobile phases A/B (60/40, *v*/*v*).

To evaluate phase I metabolites, 100 µL of thawed incubates were centrifuged (10 min, 16,550×*g*), then mixed with 100 µL phosphate buffer (pH 6) and 20 µL β-glucuronidase. After hydrolysis at 45 °C for 1 h, the reaction was quenched with 200 µL acetonitrile and 100 µL ammonium formate. Samples were centrifuged again (10 min, 2898×*g*), evaporated under nitrogen at 40 °C, and reconstituted in 30 µL mobile phase A/B (60:40, *v*/*v*).

### Human urine samples

Aliquots of 22 authentic urine samples from 16 individuals (all male, mean age 31.2 ± 5.4 years) submitted to the forensic toxicology department in Freiburg, Germany, were screened for SCRAs as part of an abstinence control program. All urines were previously tested positive for at least one of the two 3TMS-SCRAs by a targeted LC-MS/MS method including putative metabolites of the SCRAs in question. Ten samples contained only Cumyl-3TMS-PrINACA, two only ADMB-3TMS-PrINACA. Another ten urine samples were positive for both substances. To avoid bias, only samples negative for 5F- and Cumyl-PINACA and 5F- and ADB-PINACA were included in this study. No further SCRAs that could have produced similar metabolites were detected in any of the samples. Analyses were performed per client requests for abstinence verification. One of the samples originated from a legally closed death case.

To investigate phase I and II metabolites of both 3TMS-SCRAs, urine samples were processed as previously reported, with blank urine control samples prepared accordingly (Zschiesche et al. 2024). This procedure, except for the enzymatic hydrolysis step, was also applied to the blood samples and stomach contents of the deceased. All samples were reconstituted in 30 µL mobile phase A/B (60:40, *v*/*v*) prior LC-QToF-MS-analysis.

In this study, only phase I metabolites were ranked and further compared regarding their usability as consumption biomarkers, since phase II SCRA metabolites are routinely hydrolyzed prior to analysis in forensic toxicology. By hydrolyzing glucuronic acid conjugates back to their parent compounds or more easily detectable metabolites, the sensitivity and reliability of the detection are significantly increased, allowing for more accurate identification.

### In silico metabolite prediction

The simplified molecular input line entry system (SMILES) was used as inputs for the in silico algorithms. Metabolites of ADMB-3TMS-PrINACA (canonical SMILES: O=C(C1=NN(CCC[Si](C)(C)C)C2=C1C=CC=C2)NC(C(C)(C)C)C(N)=O) and Cumyl-3TMS-PrINACA (canonical SMILES: O=C(NC(C)(C)C1=CC=CC=C1)C2 = NN(CCC[Si](C)(C)C)C3=CC=CC=C32) were predicted using two in silico freeware approaches: GLORYx (de Bruyn Kops et al. 2021; Stork et al. 2020) (available at https://nerdd.univie.ac.at/gloryx/) and BioTransformer (v. 3.0) (Wishart et al. 2022) (available at https://biotransformer.ca/new). For GLORYx, the prediction “phase I and II metabolism” was used. For BioTransformer, the metabolic transformation “Human and Human Gut Microbial Transformation (AllHuman)” allowed three iterations of reactions to calculate. The “combined” CYP mode was used.

The results were used to create an inclusion list to trigger auto-MS/MS experiments by HPLC-HESI-QToF-MS. Details can be found in the supporting information (Table S1).

Since silicon was not included in the training sets for either in silico models, the Si atom was replaced with a C atom in both SCRAs for comparison purposes. For the altered ADMB-3TMS-PrINACA, *N*-(1-amino-3,3-dimethyl-1-oxobutan-2-yl)-1-(4,4-dimethylpentyl)-1*H*-indazole-3-carboxamide, in the following: “ADMB-4,4-dimethyl-PINACA” the SMILES is NC(=O)C(NC(=O)c2nn(CCCC(C)(C)C)c1ccccc12)C(C)(C)C. For “Cumyl-4,4-dimethyl-PINACA”, 1-(4,4-dimethylpentyl)-*N*-(2-phenylpropan-2-yl)-1*H*-indazole-3-carboxamide, the SMILES notation is: CC(C)(NC(= O)c2nn(CCCC(C)(C)C)c1ccccc12)c3ccccc3.

### Seized materials

Herbal blends and ash residue seized from two forensic psychiatric patients, from whom there were also urine samples available, were sent to the forensic toxicology department in Freiburg, Germany, for screening of synthetic cannabinoids via GC-MS.

Aliquots of 100 mg for herbal blend material were dissolved in 1 mL of methanol, vortexed, and centrifuged. Afterwards, 10 µL of the organic layer was evaporated to dryness and subsequently reconstituted. The joint-shaped aluminum foil containing ash residues was rinsed with 4 mL of methanol, and 50 µL of the clear supernatant were evaporated to dryness and reconstituted.

Stock solutions of ADMB-3TMS-PrINACA and Cumyl-3TMS-PrINACA (*c* = 1 mg/mL, 10 μL) were evaporated to dryness under a gentle stream of nitrogen (40 °C) and reconstituted in 100 μL of ACN.

### Instrumental

#### High-performance liquid chromatography electrospray ionization quadrupole time-of-flight mass spectrometry (HPLC-HESI-QToF-MS)

For in vivo and in vitro metabolite identification, a reversed-phase HPLC-QToF-MS, consisting of an Impact II QToF instrument with a heated-electrospray-ionization (HESI) source coupled with an Elute HPLC system (Bruker Daltonik, Bremen, Germany), was used. Chromatographic separation was achieved on a Kinetex^®^ C18 column (2.6 μm, 100 Å, 100 × 2.1 mm; Phenomenex, Aschaffenburg, Germany) applying gradient elution as follows: starting at 25% B until 0.5 min; increasing to 35% B by 4.0 min (at 0.25 mL/min); 40% B by 5.0 min; 43% B by 7.0 min (at 0.25 mL/min); 50% B by 10.5 min; 65% B by 14.5 min then raised to 95% B from 15.0 to 16.5 min; followed by re-equilibration at 25% B from 16.6 to 17.5 min. The flow rate was 0.40 mL/min except as specified otherwise.

The injection volume was 10 µL. Autosampler and column oven temperatures were set to 10 and 40 °C, respectively. HyStar™ (ver. 6.0), DataAnalysis (DA, ver. 5.6), and TASQ 2024b (Bruker Daltonik, Bremen, Germany) were used for data acquisition and processing. The MS was operated in VIP-HESI (Vacuum Insulated Probe Heated Electrospray Ionization) positive mode (*m*/*z* 50 – 650) with dry gas temperature at 230 °C (8.0 L/min), nebulizer gas pressure of 2.7 bar, and nitrogen serving as collision gas. The capillary and endplate offset voltages were 3000 and 500 V, respectively. Probe gas temperature was 400 °C with a flow of 5.0 L/min. Mass calibration was performed using sodium formate clusters and high-precision calibration mode.

Full scan and broadband collision-induced dissociation (bbCID, collision energy (CE): 30 ± 6 eV) data was acquired at a rate of 2 Hz. Spectra were compared to hypothetical metabolites derived from the biotransformations of structurally related SCRAs and in silico predictions. Hits for the molecular ions of anticipated metabolites were further analyzed in a full MS and auto-MS/MS scan to record product ion spectra. The mean area ratio (MAR [%]) was calculated by normalizing each chromatographic peak area to the respective highest abundant metabolite.

Metabolite identification (in vivo/in vitro) required precursor mass error < 5 ppm, signal-to-noise > 3:1, and fragment mass tolerance ± 10 ppm.

#### GC-EI-MS analysis of seizures/evidence material

The GC-EI-MS consisted of a 6890 series gas chromatograph equipped with an HP-5MS capillary column (30 m × 0.25 mm × 0.25 μm), combined with a 5973 series mass selective detector and a 7683 B series injector (Agilent Technologies, Waldbronn, Germany). Method details are described elsewhere (Moosmann et al. 2012), briefly: Injection volume: 1 µL, splitless; Ionization: electron ionization (EI) with 70 eV; scan mode: full scan, 50–600 *m*/*z*; run-time: 20 min; carrier gas: helium, 1.0 mL/min.

## Results

### Metabolism in vivo, in vitro, in silico

For all of the metabolites and the parent substances, the [M + H]^+^, the respective sodium adduct ([M + Na]^+^: *m*/*z* shift of + 21.9819 to [M + H]^+^) were detected.

### Metabolites of ADMB-3TMS-PrINACA in vivo and in vitro

The parent substance ADMB-3TMS-PrINACA shows a signal at *m*/*z* 389.2367 [M + H]^+^ that was also present in the pHLMs and PHH incubations at 13.1 min, and in the post mortem samples (urine, femoral and heart blood, and gastric content). In ante mortem urine samples, no ADMB-3TMS-PrINACA could be detected. ADMB-3TMS-PrINACA shows fragments of *m*/*z* 73.0468 (trimethylsilyl cation), 145.0396 (indazole acylium cation), and 259.1261 (3TMS-PrINACA indazolyl acylium cation) as well as *m*/*z* 277.1367. The ion *m*/*z* 344.2153 can be explained by alpha cleavage.

**Fig. 2:**
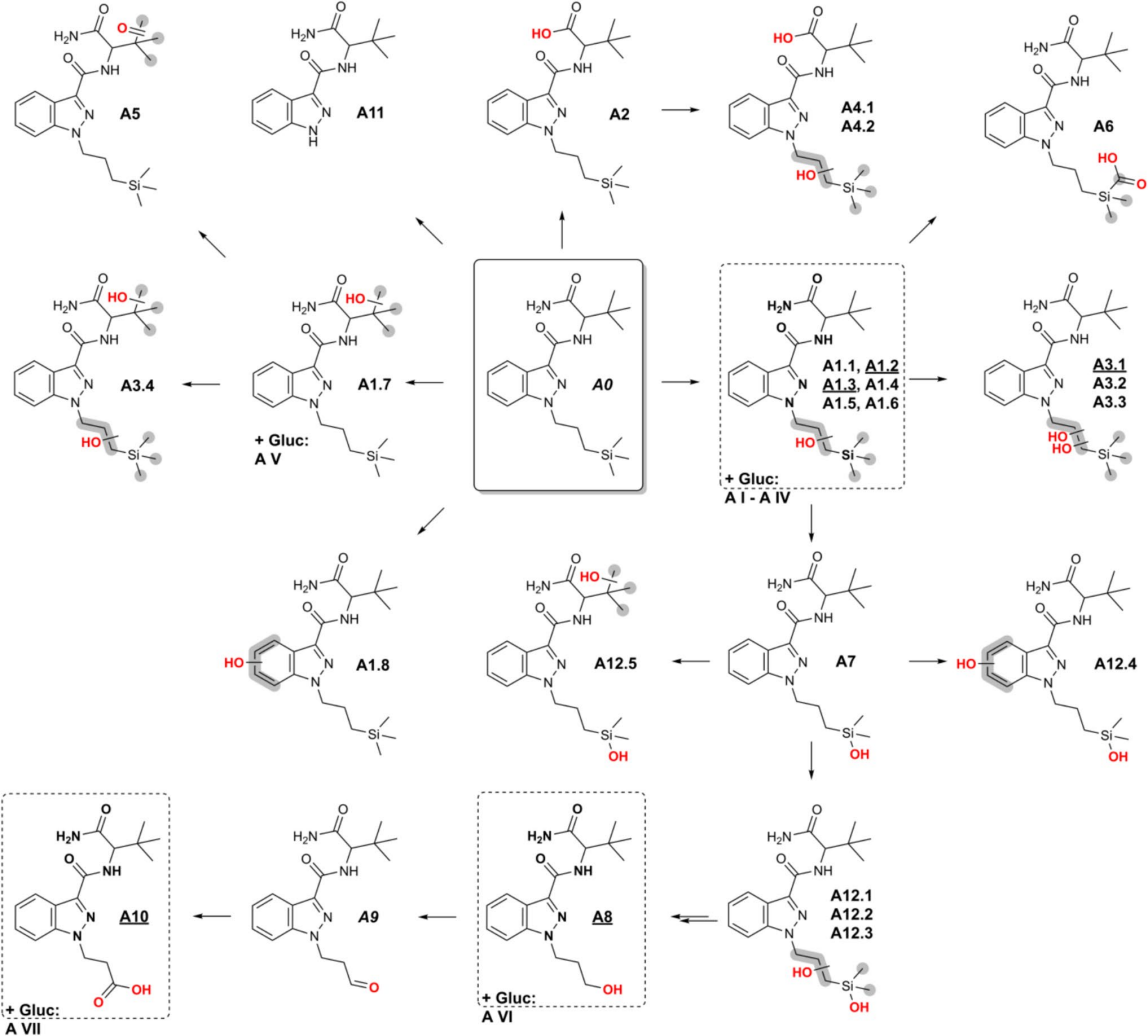
Postulated structures of metabolites of ADMB-3TMS-PrINACA found in vitro (pHLMs and PHH) and in vivo (urine) and their proposed metabolic pathways. Metabolites in dashed line boxes and underlined IDs are suggested as suitable urinary biomarkers. IDs in *italics* were only found in vitro. *Gluc* glucuronic acid conjugate. It was not possible to determine the exact site of biotransformation for the structural units highlighted in gray. The double arrows indicate a potentially complex, multi-step biotransformation process

**Fig. 3:**
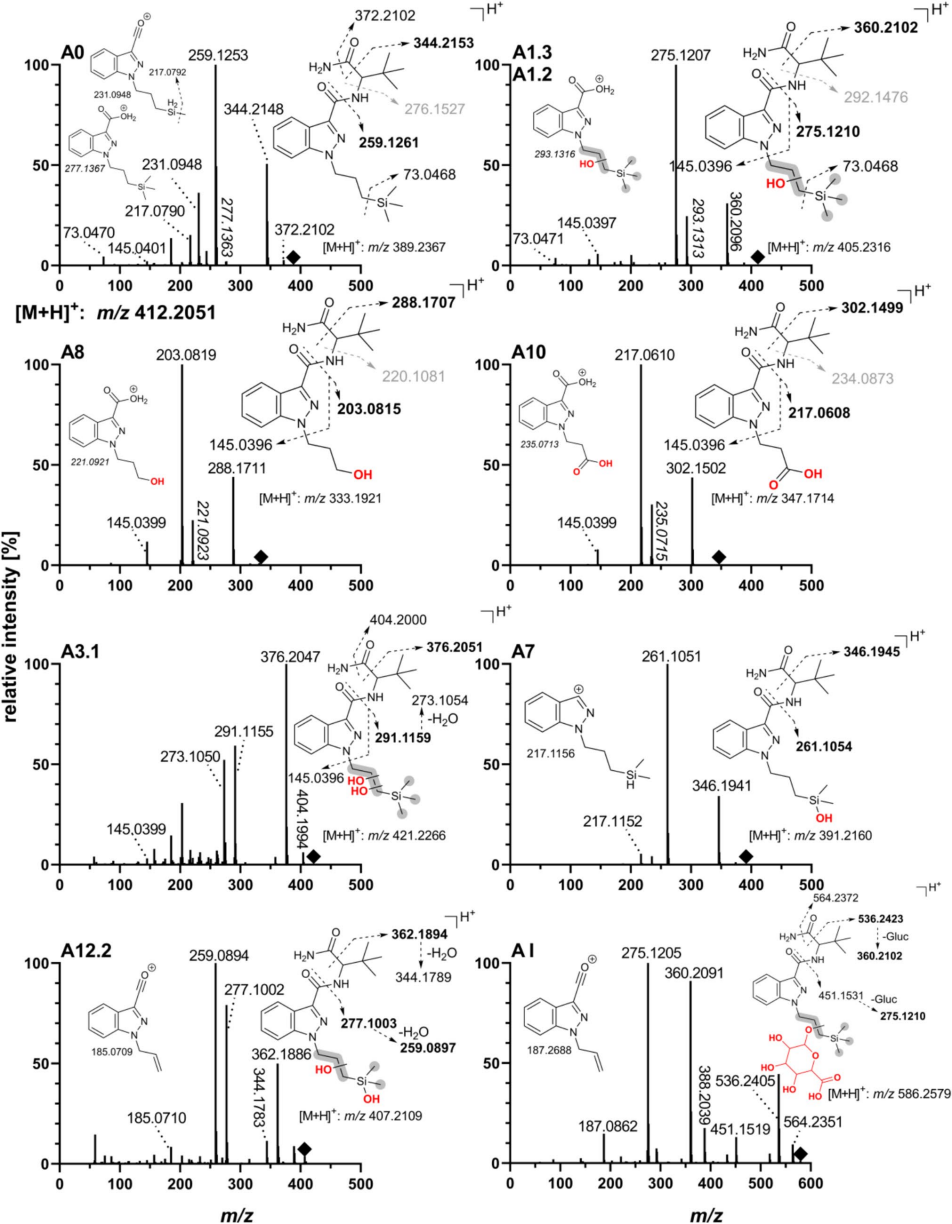
Product ion spectra and suggested fragmentation patterns of ADMB-3TMS-PrINACA and its most abundant metabolites (autoMS/MS) obtained by LC-QToF-MS in positive HESI mode. Structural fragment ions in **bold** represent those with the highest intensities, suggested as markers for screening. Fragments with *m*/*z* values in *italics* indicate a possible “*N*–*O*-exchange”, and therefore, presumed fragments shown in light gray are not present in the fragmentation spectra (Fig. S5). Squares denote the precursor ion. The collision energy was dependent on the precursor mass, with 25 eV for *m*/*z* 300 and 32 eV for *m*/*z* 500

The resulting fragment ions of the biomarkers vary depending on the structural differences between the identified metabolite and ADMB-3TMS-PrINACA. Metabolites detected in vivo and in vitro were assigned identifiers corresponding to their biotransformation stage (oxidation, hydrolysis, conjugation, etc.) and subsequently ordered according to their retention times. In Fig. 2, biomarkers of ADMB-3TMS-PrINACA in all three models are illustrated. The most abundant and mechanistically interesting phase I biomarkers are listed in Table 1; Table S2 lists all tentatively identified phase I metabolites. Phase II metabolites in urine and PHH can be found in Table S3. The fragment spectra of the parent compound and its most abundant metabolites are depicted in Fig. 3.

**Table 1:** Metabolic transformation, elemental composition, retention time (*t*_R_), accurate mass of molecular ion in positive ionization modes ([M + H]^+^) with mass error, diagnostic product ions, and in vivo and in vitro mean area ratios (MAR, [%]) based on relative peak areas of LC-QToF-MS analysis for phase I metabolites of ADMB-3TMS-PrINACA

ID	*t*_R_ (min)	Metabolic transformation (location)	Elemental composition	Calculated* m*/*z* [M + H]^+^ (Δppm)	Diagnostic product ions *m*/*z*	MAR in vivo [%]	MAR in vitro [%]	
						Urines	pHLMs	PHH
**A1.2**	**7.02**	**Hydroxylation (S)**	**C**_**20**_**H**_**32**_**N**_**4**_**O**_**3**_**Si**	**405.2316 (1.6)**	**275.1210; 293.1316; 360.2102**	**78.37%**	54.03%	33.98%
**A1.3**	**7.96**	**Hydroxylation (S)**	**C**_**20**_**H**_**32**_**N**_**4**_**O**_**3**_**Si**	**405.2316 (1.1)**	**275.1210; 293.1316; 360.2102**	**100.00%**	100.00%	100.00%
**A3.1**	**4.34**	**Dihydroxylation (2 × S)**	**C**_**20**_**H**_**32**_**N**_**4**_**O**_**4**_**Si**	**421.2266 (0.4)**	**376.2051; 291.1159; 273.1054**	**57.96%**	13.59%	1.94%
A8	2.91	Cleavage 3TMS + terminal hydroxylation (S)	C_17_H_24_N_4_O_3_	333.1921 (− 1.0)	203.0815; 220.1081; 288.1707	48.39%	26.65%	9.68%
A10	2.97	Cleavage 3TMS + carboxylic acid formation (S)	C_17_H_22_N_4_O_4_	347.1714 (0.5)	217.0608, 234.0873; 302.1499	53.27%	0.99%	0.90%
A12.2	3.63	Demethylation (S) + Dihydroxylation (2xS)	C_19_H_30_N_4_O_4_Si	407.2109 (− 0.2)	259.0897; 277.1003; 145.0396	41.73%	1.46%	0.24%
A0	13.10	ADMB-3TMS-PrINACA (Parent)	C_20_H_32_N_4_O_2_Si	389.2367 (0.5)	259.1261; 344.2153; 73.0468	□	■	■

Only hydrolyzed urines and PHH supernatants are shown. The three most abundant phase I metabolites found in urine samples are highlighted in **bold**. The parent substance was excluded from the MAR calculation. Table S2 lists all phase I metabolites tentatively identified in this study

*t*_*R*_ retention time, *S* side chain (3TMS-PrINACA), *pHLM* pooled liver microsomes, *PHH* primary human hepatocytes, ■ detected but not included in MAR, □ not detected

In total, 27 phase I metabolites of ADMB-3TMS-PrINACA were identified across in vivo (urine) and in vitro (pHLMs, PHH) models, deriving from combinations of monohydroxylation (8 metabolites), dihydroxylation (5 metabolites), hydrolysis (4 metabolites, including 2 combined with hydroxylation), ketone or aldehyde formation (2 metabolites), one acid formation, one *N*-dealkylation, and cleavage of the 3TMS group followed by further oxidation or functionalization (3 metabolites). Six metabolites (A7, A12.1 – A12.5) underwent multiple modifications, specifically demethylation combined with mono- or dihydroxylation. Most of the biotransformation steps occurred at the 3TMS-PrINACA side chain. Only a few occurred at the ADMB-linked group or the indazole core. The most abundant metabolite at 100% MAR (overall rank: 1) in all matrices was A1.3, a monohydroxylation at the side chain. Characteristic fragment ions facilitated structural elucidation, *m*/*z* 145.0396, reflecting the unaltered INACA moiety and *m*/*z* 73.0468 was further indicative of silylated structures. A1.2, also a hydroxylated species, is ranked 2nd in vivo and in vitro on an area basis, whereas a dihydroxylated metabolic product on the side chain (A3.1) ranked 3rd in urine. The study could not clarify if the metabolic reaction occurred on the propyl side chain or the 3TMS group. The metabolite A8, based on 3TMS cleavage and further terminal hydroxylation (rank 5 in urine, 4th in pHLM, and 3rd in PHH), showed the characteristic fragment *m*/*z* 203.0815 (hydroxylated *N*-propylindazole acylium cation). Further oxidation of A8 to the *N*-propionic acid metabolite A10 ranked 4th in urine but had relatively low levels in vitro. A10 showed the diagnostic fragment *m*/*z* 217.0608, indicating a carboxylic acid structure. Incubations of ADB-PINACA and 5F-ADB-PINACA in pHLM assays did not result in the generation of metabolites A8 and A10 (Figs. S1 and S2).

The *N*-propionic acid metabolite (A10) was detected in the deceased's stomach contents, femoral, and heart blood, but the *N*-3-hydroxypropyl metabolite (A8) could not be detected. In heart blood and urine, relatively high-intensity signals for monohydroxylated species were observed.

Since no UDPG-generating system was added to the pHLM incubations, only phase I metabolites were generated. In human urine samples, a total of 7 phase II metabolites for ADMB-3TMS-PrINACA – all of them were *O*-glucuronic acid conjugates – could be detected; whereas in PHH incubation, only three of these were present (Table S3). Assigning structures based on fragmentation was not possible. However, the elution sequence is expected to be identical to that of the monohydroxylated species. A I seemed to be the most abundant phase II metabolite in urine. A8 is the corresponding aglycone of A VI and A10 of A VII.

### Metabolites of Cumyl-3TMS-PrINACA in vivo and in vitro

**Fig. 4:**
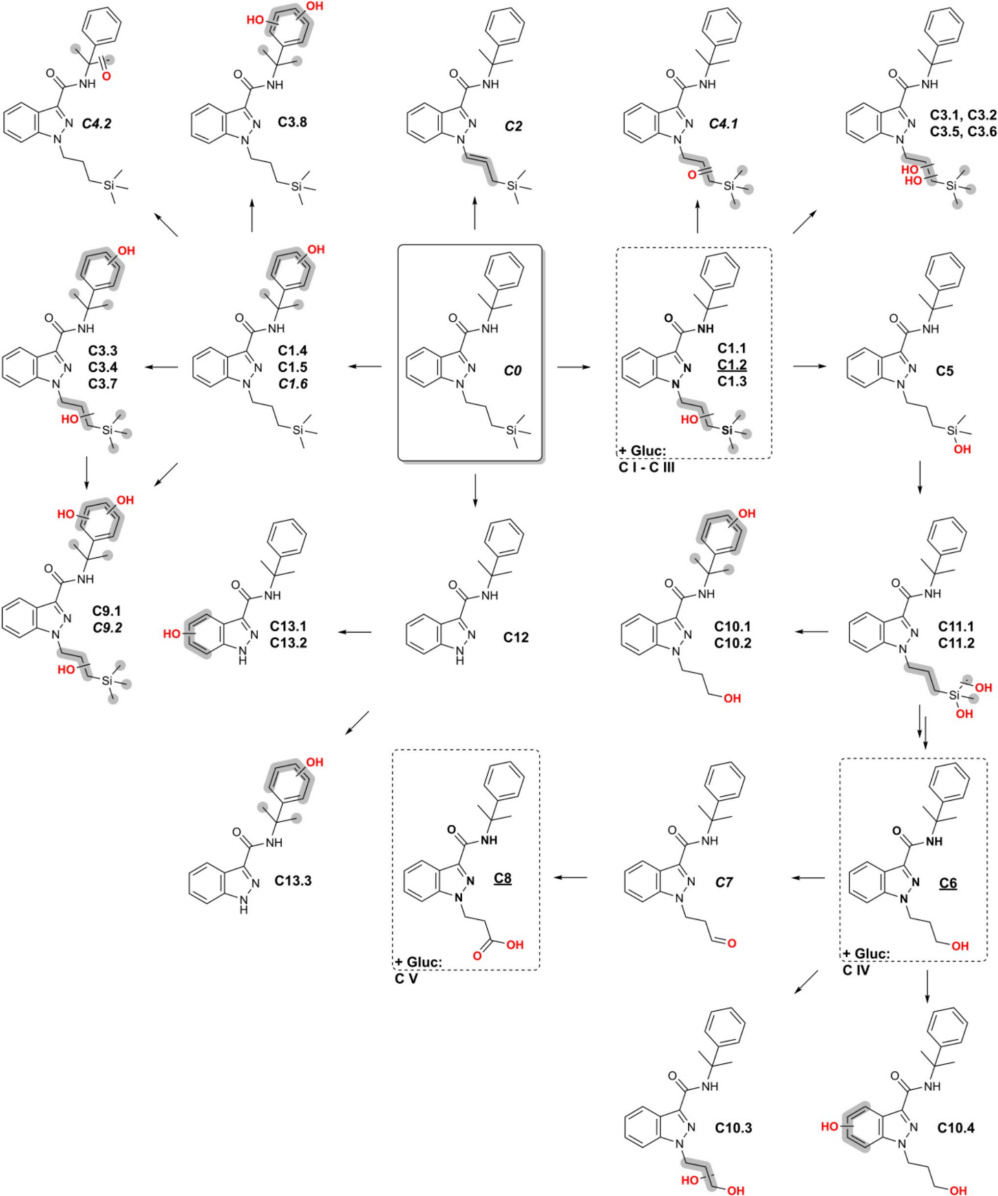
Postulated structures of metabolites of Cumyl-3TMS-PrINACA found in vitro (pHLMs and PHH) and in vivo (urine) and their proposed metabolic pathways. Metabolites in dashed line boxes and underlined IDs are suggested as suitable urinary biomarkers. IDs in *italics* were only found in vitro. *Gluc* glucuronic acid conjugate. It was not possible to determine the exact site of biotransformation for the structural units highlighted in gray. The double arrows indicate a potentially complex, multi-step biotransformation process

**Fig. 5:**
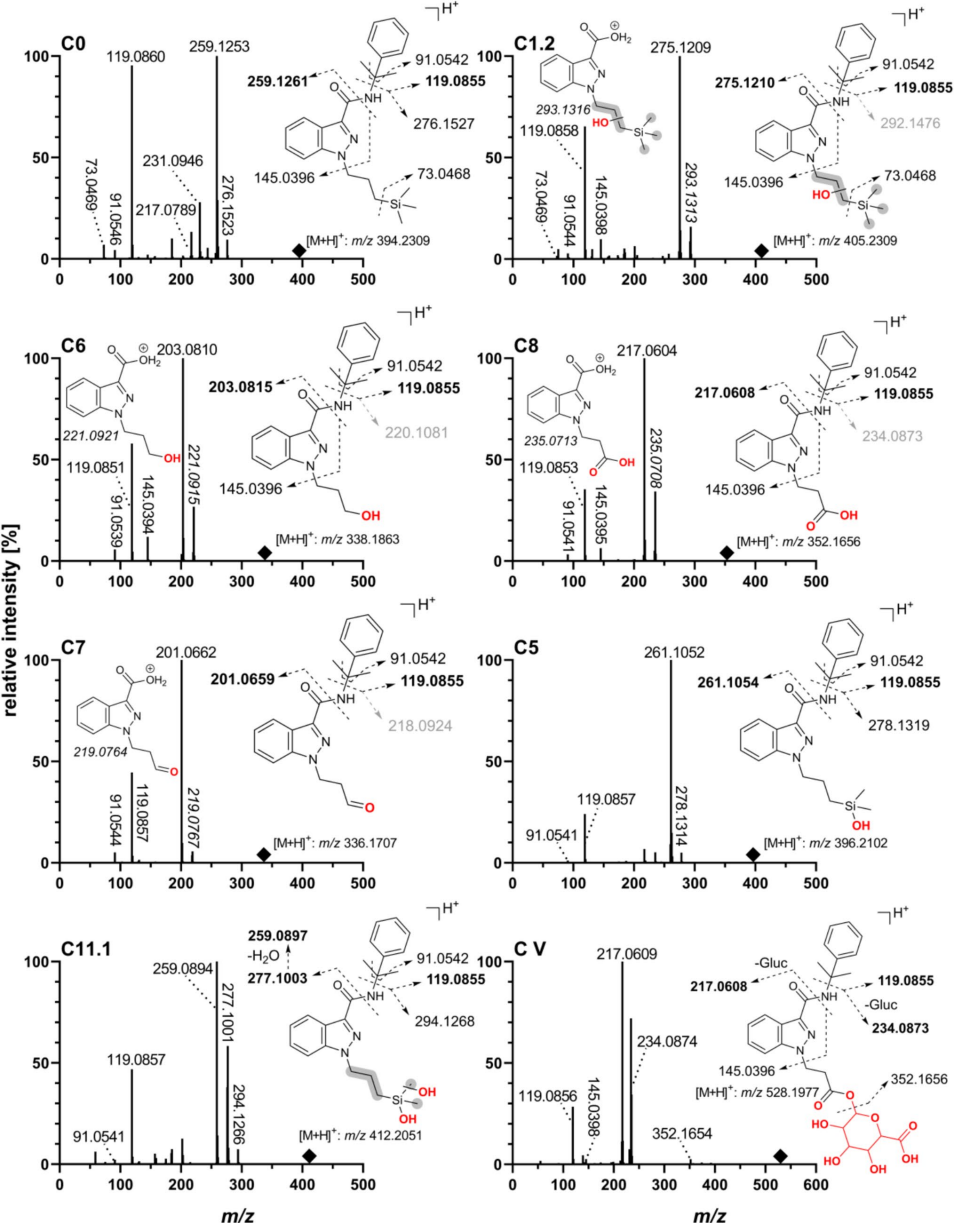
Product ion spectra and suggested fragmentation patterns of Cumyl-3TMS-PrINACA and its most abundant metabolites (autoMS/MS) obtained by LC-QToF-MS in positive HESI mode. Structural fragment ions in **bold** represent those with the highest intensities, suggested as markers for screening. Fragments with *m*/*z* values in *italics* indicate a possible “*N*–*O*-exchange”, and therefore, presumed fragments shown in light gray are not present in the fragmentation spectra (Figs. S5 and S6). Squares denote the precursor ion. The collision energy was dependent on the precursor mass, with 25 eV for *m*/*z* 300 and 32 eV for *m*/*z* 500

Cumyl-3TMS-PrINACA, the parent substance, shows an [M + H]^+^ ion at *m*/*z* 394.2309 and elutes at 16.02 min. It was observed in pHLM and hepatocyte incubations but was not detected in urine. The compound shows characteristic fragments at *m*/*z* 73.0468, 91.0542 (tropylium ion), 119.0855 (cumyl ion), 145.0396 (indazole acylium cation), and 259.1261 (3TMS-PrINACA indazolyl acylium cation). Figure 4 presents the detected metabolites of Cumyl-3TMS-PrINACA. The most abundant and mechanistically interesting phase I biomarkers are listed in Table 2. A complete list of all identified phase I metabolites is provided in Table S4, whereas phase II metabolites are given in Table S5. The fragmentation spectra of the parent compound and potential biomarkers are illustrated in Fig. 5.

**Table 2:** Metabolic transformation, elemental composition, retention time (*t*_R_), accurate mass of molecular ion in positive ionization modes ([M + H]^+^) with mass error, diagnostic product ions, and in vivo and in vitro mean area ratios (MAR, [%]) based on relative peak areas of LC-QToF-MS analysis for phase I metabolites of Cumyl-3TMS-PrINACA

ID	*t*_R_ (min)	Metabolic transformation (location)	Elemental composition	Calculated* m*/*z* [M + H]^+^ (Δppm)	Diagnostic product ions *m*/*z*	MAR in vivo [%]	MAR in vitro [%]	
						Urine	pHLMs	PHH
**C1.2**	**13.18**	**Hydroxylation (S)**	**C**_**23**_**H**_**31**_**N**_**3**_**O**_**2**_**Si**	**410.2258 (− 0.58)**	**275.1210; 119.0855; 145.0396**	**100.00%**	74.36%	70.84%
C5	11.44	Demethylation + hydroxylation (S)	C_22_H_29_N_3_O_2_Si	396.2102 (**− **1.27)	261.1054; 278.1319; 119.0855	1.78%	22.26%	21.30%
**C6**	**6.92**	**Cleavage 3TMS + terminal hydroxylation (S)**	**C**_**20**_**H**_**23**_**N**_**3**_**O**_**2**_	**338.1863 (0.26)**	**203.0815; 119.0855; 145.0396**	**75.48%**	100.0%	100.0%
C7	8.17	Cleavage 3TMS + terminal aldehyde formation (S)	C_20_H_21_N_3_O_2_	336.1707 (**− **0.42)	201.0658; 119.0855	□	34.14%	13.70%
**C8**	**6.87**	**Cleavage 3TMS + carboxylic acid formation (S)**	**C**_**20**_**H**_**21**_**N**_**3**_**O**_**3**_	**352.1656 (0.06)**	**217.0608; 119.0855; 145.0396**	**70.66%**	32.83%	59.30%
C11.1	7.66	Demethylation (S) + dihydroxylation (2 × S)	C_22_H_29_N_3_O_3_Si	412.2051 (**− **0.51)	259.0897; 277.1003; 119.0855	16.74%	19.25%	26.37%
C0	16.02	Cumyl-3TMS-PrINACA (Parent)	C_23_H_31_N_3_OSi	394.2309 (**− **0.23)	259.1261; 119.0852; 73.0466	□	■	■

Only hydrolyzed urine samples and PHH supernatants are listed. The three most abundant phase I metabolites found in urine samples are highlighted in **bold**. The parent substance was excluded from the MAR calculation. Table S4 lists all phase I metabolites tentatively identified in this study

*t*_*R*_ retention time, *S* side chain (3TMS-PrINACA), *pHLM* pooled liver microsomes, *PHH* primary human hepatocytes, ■ detected but not included in MAR, □ not detected

A total of 33 metabolites of Cumyl-3TMS-PrINACA could be detected. Again, these metabolites arise through diverse biotransformations involving oxidation, demethylation, and alkyl chain cleavage. Six metabolites derived from monohydroxylation at the side chain and the cumyl moiety. A shift of 15.9949 Da (oxygen) from the unaltered cumyl ion *m*/*z* 119.0855 to 135.0804 indicates hydroxylation. Among these, C1.2 was the most abundant metabolite detected in all systems, ranking 1st in urine and 2^nd^ *in vitro*, and is characterized by a diagnostic fragment at *m*/*z* 275.1210 and unaltered fragments compared to the parent compound at *m*/*z* 145.0396 and 119.0855, indicating hydroxylation at the side chain. Furthermore, dihydroxylated (8), ketone/aldehyde (2), triols (2), demethylation and further mono- or dihydroxylation (3), and *N*-dealkylated metabolites including hydroxylated forms (4) were observed. Two of the last-mentioned metabolites showed a fragment at *m*/*z* 161.0346, a hydroxylated indazole core. However, hydroxylation, especially at the side chain and cumyl moiety, was the predominant pathway. A metabolic pathway of Cumyl-3TMS-PrINACA involves cleavage of the 3TMS group, followed by further oxidation as well. Cleavage and terminal hydroxylation lead to C6 ranking 1st in vitro and 2nd in vivo. Oxidation of C6 to the likely aldehyde intermediate C7 was observed only in vitro. The final oxidation to the *N*-propionic acid metabolite (C8) is ranked 3rd in urine and PHH and 5th in pHLM. C6 and C8, lacking the 3TMS group, exhibited characteristic fragments of *m*/*z* 203.0815 and 217.0608. C6 was also further hydroxylated, resulting in 4 isomers.

The biomarkers C6 and C8 were also formed in pHLM incubations of Cumyl-PINACA with an increase in area after 1 h incubation compared to 0.5 h. In 5F-Cumyl-PINACA incubations, only C6 was slightly detectable after 1 h (Figs. S3 and S4).

Five *O*-glucuconjugated phase II metabolites could be observed in human urine samples, four of them in human hepatocytes (Table S5). C V was the most abundant phase II metabolite in urine samples; in hepatocytes, it was C I. C1.1 is most likely the aglycone of C I, C1.2 of C II and C1.3 of C III. The highly abundant phase I metabolites C6 (C IV) and C8 (C V) formed conjugates with glucuronic acid as well.

### In silico metabolite prediction

Using GLORYx for ADMB-3TMS-PrINACA, 28 metabolites were predicted (Table S6). Of these, 26 were phase I metabolites, primarily involving monohydroxylations with mostly high priority scores. The remaining phase I reactions included several hydrolysis reactions at the primary and secondary amides, dealkylations, and dehydrogenations. Two of the predicted metabolites were phase II biotransformation products formed by *N*-glucuronidation of the parent compound.

For Cumyl-3TMS-PrINACA, GLORYx predicted 25 metabolites (Table S7). These were formed primarily through a monohydroxylation step with high priority scores, along with hydrolysis, a carboxylic acid formation, dealkylations, and dehydrogenations. Additionally, two phase II metabolites were predicted.

BioTransformer was only able to predict the *N*-glucuronidation of the amide of ADMB-3TMS-PrINACA, which was also predicted by GLORYx. No metabolites could be predicted for Cumyl-3TMS-PrINACA. For the molecule resulting after formal exchange of the silicon in ADMB-3TMS-PrINACA by a carbon atom (“ADMB-4,4-dimethyl-PINACA”), 143 biomarkers were predicted, featuring several hydroxylations, along with further *O*-glucuronidations, *O*-sulfatations, and oxidations of primary alcohols to aldehydes. The Si–C exchange in the former Cumyl-3TMS-PrINACA (“Cumyl-4,4-dimethyl-PINACA”) resulted in 246 predicted metabolites, which exhibited analogous biotransformation products. The results can be found in Tables S8 and S9. For completeness, this experiment was also conducted with GLORYx. For both substances without a silicon atom, analogous metabolites were predicted with the quaternary carbon atom. The results can be found in Table S10 and S11. The priority scores were largely consistent.

### Seized materials

**Fig. 6:**
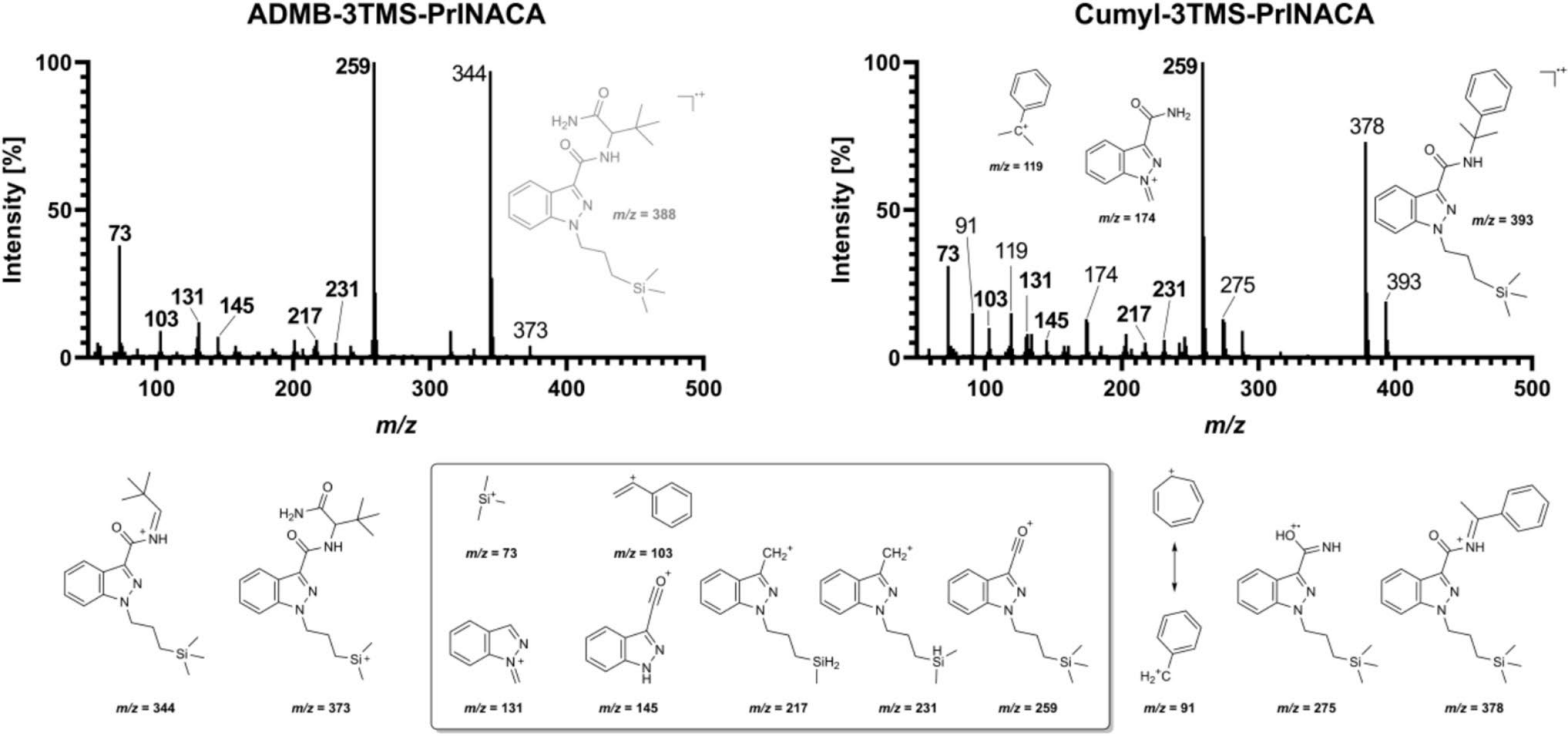
GC-EI-MS spectra of the herbal blend (left, ADMB-3TMS-PrINACA) and the ash (right, Cumyl-3TMS-PrINACA). The tentative fragments shown in the box are identical for both SCRAs. Fragments displayed left and right of the box, or in the respective MS spectrum, were detected for that specific SCRA exclusively

Figure 6 shows the GC-MS spectra of the seized material. In this case, this was a herbal blend containing Cumyl-3TMS-PrINACA and rests of ash in aluminum foil positive for ADMB-3TMS-PrINACA. Additionally, in the mentioned post-mortem case, a herbal blend containing ADMB-3TMS-PrINACA was found at the scene.

Only the molecular ion peak of Cumyl-3TMS-PrINACA (*m*/*z* 393) could be detected. For both SCRAs, the acylium-indazole-alkyl ion (*m*/*z* 259) represents the base peak. The indazole core structure is represented by the fragments *m*/*z* 103, 131, and 145. The unique trimethylsilyl ion, corresponding to *m*/*z* 73, can be observed in both EI-MS spectra. For Cumyl-3TMS-PrINACA, the tropylium ion (*m*/*z* 91) and the cumyl ion (*m*/*z* 119) are characteristic fragments.

### Prevalence of both synthetic cannabinoids

The first urine specimens positive for ADMB-3TMS-PrINACA excretion products dated to late December 2022. The last samples positive for this substance were from early June 2023. Over the two-year period from December 2022 to December 2024, 25.64% of all urine samples tested for synthetic cannabinoids yielded positive results (*N* = 3085). Among these, samples positive for ADMB-3TMS-PrINACA accounted for a small fraction of 0.42% (*N* = 13). In 9 cases, Cumyl-3TMS-PrINACA was detected alongside ADMB-3TMS-PrINACA.

In contrast, urine samples from Cumyl-3TMS-PrINACA users were not received until the end of March 2023. By December 2024, Cumyl-3TMS-PrINACA showed a higher prevalence compared to ADMB-3TMS-PrINACA. During the 2-year observation period, Cumyl-3TMS-PrINACA was detected in 7.29% of all SCRA-positive samples (*N* = 225). In 77.78% of these, Cumyl-3TMS-PrINACA was the only SCRA detected. The most frequently co-consumed SCRAs were MDMB-4en-PINACA (9.33%), Cumyl-PINACA (8.89%), and ADB-BUTINACA (7.11%). 5F-Cumyl-PINACA was co-ingested in 1.78% of the cases. Since Cumyl-PINACA, 5F-Cumyl-PINACA, and Cumyl-3TMS-PrINACA yield common metabolites, differentiation was achieved by identifying substance-specific monohydroxylated metabolites.

**Fig. 7:**
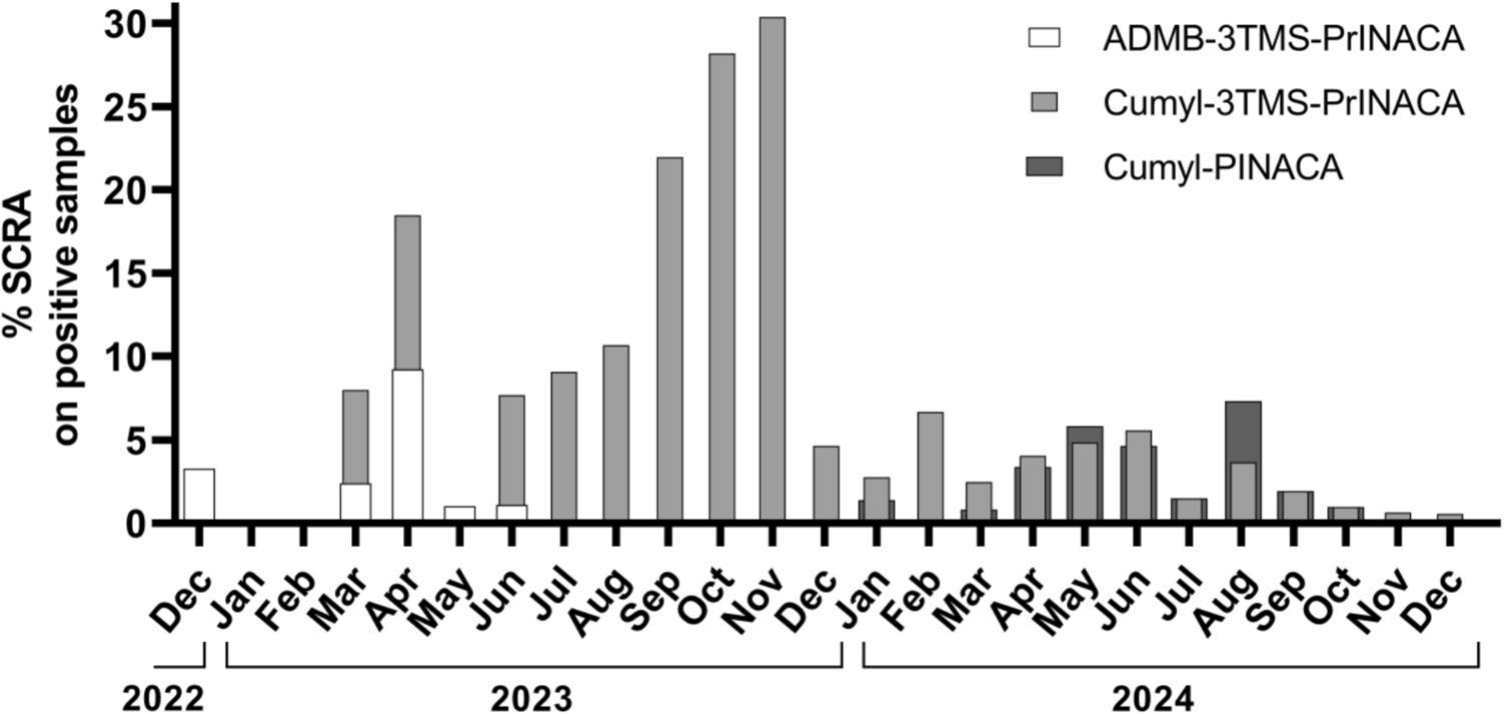
Prevalence of metabolites of ADMB-3TMS-PrINACA (white), Cumyl-3TMS-PrINACA (light gray), and Cumyl-PINACA (dark gray) in SCRA positive urine samples sent to the Institute of Forensic Medicine, Freiburg, Germany

A graphical representation and further details on the prevalence of ADMB-, Cumyl-3TMS-PrINACA, and Cumyl-PINACA from December 2022 to December 2024 are given in Fig. 7.

One fatal case involving ADMB-3TMS-PrINACA was analyzed. ADMB-3TMS-PrINACA was detected at approximate concentrations of 6.6 ng/mL (femoral blood) and 58 ng/mL (cardiac blood), alongside other drugs, indicating a mixed intoxication. Further details of the post-mortem case can be found in the supporting information (Table S12).

## Discussion

### Metabolism in vivo, in vitro, in silico

#### Comparison of metabolites of ADMB- and Cumyl-3TMS-PrINACA in vivo and in vitro

The cumyl group increases Cumyl-3TMS-PrINACA’s lipophilicity, causing it and its metabolites to elute later in reversed-phase liquid chromatography than ADMB-3TMS-PrINACA and its metabolites. For both 3TMS-featuring SCRAs, the predominant and specific metabolites in human urine appear to be monohydroxylated species (**A1.3**, **A1.2** and **C1.2**). Monohydroxylations are commonly described as primary biotransformation products for many SCRAs (Diao and Huestis 2019; Giorgetti et al. 2024; Zschiesche et al. 2024). Both compounds also produce highly abundant but nonspecific biomarkers (*N*-3-OH-propyl, **A8/C6**; and *N*-propionic acid, **A10/C8**), likely from oxidative 3TMS side chain cleavage. The proposed *N*-propionic acid metabolite may alternatively be an *N*-propyl hydroxyketone, but literature supports carboxylic acid formation via β-oxidation of *N*-pentyl side chain based SCRAs (Hutter et al. 2013; Zschiesche et al. 2024), presumably explaining the formation of C8 seen in Cumyl-PINACA and 5F-Cumyl-PINACA pHLM incubations. To differentiate between Cumyl-3TMS-PrINACA and Cumyl-PINACA/5F-Cumyl-PINACA intake, compound specific targets, e.g., C1.2 must be included in screening methods. The exact mechanism of 3TMS-cleavage remains unclear. Notably, ADB-PINACA and 5F-ADB-PINACA pHLM incubations lack A8 and A10 despite the formation of the *N*-pentanoic acid metabolite being known (Carlier et al. 2017; Schwartz et al. 2015), which is a substrate for β-oxidation and loss of the C_2_ unit. Fragment ions of A10 and C8 exhibit retention time shifts when compared to the isobaric metabolites of ADB-BUTINACA and Cumyl-4CN-BINACA in the same LC-MS/MS run. Intermediate aldehyde metabolites (A9, C7) were only observed in vitro, suggesting rapid in vivo oxidation to *N*-propionic acids, likely via aldehyde dehydrogenase, consistent with aldehyde instability (Holm et al. 2016).

Both 3TMS-SCRAs produced a metabolite likely formed by oxidative Si-demethylation of the 3TMS group, resulting in a (hydroxy(dimethyl)silyl)propyl side chain (A7, C5). These silanolized metabolites were also reported for 1S-LSD in vitro recently (Azuma et al. 2025). The metabolites A7 and C5 may be the predecessors to A12.1 – A12.3 and C11.1, C11.2, which result from hydroxylation of the side chain. Based on A12.1 – A12.3 and C11.1, C11.2, the biomarkers C6 and A8 could result from the cleavage of the former 3TMS group. Alternatively, C6 and A8 could be formed by a mechanism similar to that described above for SCRAs with an *N*-pentyl side chain. C- or Si-demethylation, typically catalyzed by cytochrome P450 demethylases, involves hydroxylation, rearrangement to a hemiacetal, and formaldehyde elimination (Sarai et al. 2024; Jiang et al. 2013). Further hydroxylation of the Si atom likely follows. For compounds with a *tert* butyl instead of a 3TMS group, a similar process was observed for the antiviral drug ombitasvir. An oxidative C-demethylation was followed by subsequent monohydroxylation in this case (Shen et al. 2016).

Ombitasvir and finasteride – also carrying a *tert*-butyl group – undergo sequential hydroxylation to a tert-butanol and oxidation to a 2,2-dimethylpropanoic acid (Huskey et al. 1995; Lundahl et al. 2009; Shen et al. 2016). Metabolites A1.1 – A1.6 and C1.1 – C1.3, indistinguishable by the fragmentation of the *N*-propyl or 3TMS groups, likely correspond to tert-butanol derivatives. Metabolite A6 may represent the analogous 2,2-dimethylpropanoic acid structure. However, no acid metabolite could be detected for Cumyl-3TMS-PrINACA, either because its abundance was too low or because it quickly reacted further to form metabolite C8 by β-oxidation. A similar mechanism is suggested for the metabolite A10, resulting from the ADMB-3TMS-PrINACA carboxylic acid metabolite A6.

The *N*-dealkylated metabolites ADMB-INACA (ADB-INACA), confirmed with a reference standard and Cumyl-INACA, could only be detected to a limited extent across all three matrices. This was also documented in previous studies (Kavanagh et al. 2022; Zschiesche et al. 2024). Neither ADMB- nor Cumyl-3TMS-PrINACA was detected in urine from living persons. This is consistent with most in vivo metabolism studies (Diao and Huestis 2019; Zschiesche et al. 2024).

Reference standards for metabolites of newly emerged NPS are often unavailable. Biotransformation products were identified tentatively based on retention times and fragmentation patterns. Precise biotransformation sites require authentic standards or compound isolation with structural elucidation by NMR spectroscopy. Peak area rankings do not reflect absolute concentrations due to ionization variability, matrix effects, and unknown drug use parameters (frequency, administration route, timing, and dose). Despite these limitations, this approach aids in identifying metabolites as target analytes. Individual metabolite profiles may also vary with CYP enzyme polymorphisms (Giorgetti et al. 2023; Musshoff et al. 2010).

Both pHLMs and PHH generally showed good agreement with the metabolites detected in human urine. Compared to PHH and animal studies, pHLMs are an easy-to-use and cost-effective alternative. Enzymatic hydrolysis is recommended to increase the sensitivity of metabolite detection.

ADMB-3TMS-PrINACA (*m*/*z* 277.1367) and many of the metabolites discussed exhibited a fragment suggesting an “*N*–*O* exchange”. This phenomenon was also observed in some other SCRAs. Possible mechanistic details are given in the supporting information.

### In silico metabolite prediction

Both in silico prediction models failed to predict biomarkers for ADMB- and Cumyl-3TMS-PrINACA after 3TMS-group cleavage. However, GLORYx accurately predicted at least three monohydroxylated side chain metabolites for both 3TMS-SCRAs, with monohydroxylation at the 3TMS moiety ranked first for both compounds. Interestingly, BioTransformer encountered significant limitations in predicting metabolites for these substances. However, both in silico models were trained and developed exclusively on compounds containing only carbon, hydrogen, oxygen, nitrogen, sulfur, phosphorus, or halogen atoms but no silicon (de Bruyn Kops et al. 2021; Wishart et al. 2022). For BioTransformer, other settings were tested, but none predicted more than the previously described phase II metabolite of ADMB-3TMS-PrINACA.

The input of the SMILES of molecules with Si substituted by C in BioTransformer led to an increase in predicted metabolites for “ADMB-4,4-dimethyl-PINACA” and “Cumyl-4,4-dimethyl-PINACA”, highlighting difficulties in processing silicon atoms. In contrast, GLORYx handled silicon-containing compounds more effectively, despite their absence from its training set, suggesting an ability to recognize silicon analogs of carbon compounds. However, neither GLORYx nor BioTransformer could predict metabolites resulting from cleavage and subsequent oxidation of the 3TMS or tert-butyl group cleavage. Despite the in silico models’ current limitations in handling silicon-containing compounds, these analyses illuminate critical areas for model improvement and suggest the need for diverse training datasets to enhance prediction accuracy.

Various in silico models, including GLORYx and BioTransformer, have been assessed for metabolite prediction, but no single model outperforms the others. Combining multiple models is therefore strongly advised to enhance accuracy (Scholz et al. 2023; Pelletier et al. 2025). In silico software provides a time- and cost-efficient alternative to in vitro experiments, offering initial insights in untargeted analyses, especially when reference materials are lacking (Kirchmair et al. 2015; Zschiesche et al. 2024).

### Seized materials

The stable fragment *m*/*z* 73 is often referred to as characteristic for substances bearing a 3TMS moiety, and common fragments can be observed by the loss of a methyl radical from the 3TMS group (Harvey and Vouros 2020). Both SCRAs showed the base fragment generated by alpha cleavage of the carbonyl group (*m*/*z* 259), and after a hydrogen shift and a rearrangement, the acylium-indazole (*m*/*z* 145) and the methylidene-indazolium ion (*m*/*z* 131) were reported for indazole core containing SCRAs (Liu et al. 2021; Luo et al. 2024). However, the tropylium (*m*/*z* 91) and the cumyl ion (*m*/*z* 119) are typical fragments in GC-EI-MS for Cumyl-containing SCRAs (Angerer et al. 2018).

The presence of specific SCRAs in the seized material, along with their metabolites detected in the exhibit owner’s urine, confirms the consumption of the respective SCRA.

### Prevalence of both synthetic cannabinoids

A possible explanation for the lower prevalence of ADMB- compared to Cumyl-3TMS-PrINACA could be the higher potency at the human cannabinoid receptor 1 (hCB_1_) of the former, leading users to potentially avoid repeated consumption. However, a decline in positive samples was noted in December 2023, before the 3TMS propyl moiety as a side chain for SCRAs was added to the German New Psychoactive Substances Act (NpSG, Neues-psychoaktive Stoffe-Gesetz) in June 2024 (Bundesministerium für Gesundheit 2024).

When comparing SCRAs with the same scaffold but different head-groups, ADMB-containing SCRAs generally appear to be more potent than their cumyl counterparts, depending on the assay used (Grafinger et al. 2021). Currently, no pharmacological or toxicological data are available for ADMB-3TMS-PrINACA and Cumyl-3TMS-PrINACA, highlighting the need for further research. Their structural similarity to potent SCRAs implies they likely show strong cannabinoid receptor activation.

During the period of the study, ADB-BUTINACA and MDMB-4en-PINACA were the most prevalent SCRAs, with prevalences of 21.98% and 32.25% of positive SCRA urine findings, respectively. However, these prevalence data are limited to German forensic psychiatric patients and prison inmates undergoing abstinence testing and cannot be generalized to the broader population. Additionally, the number of positive specimens tested should be increased to enhance the statistical robustness of the findings.

## Conclusions

Detecting the intake of synthetic cannabinoids in urine remains a challenge for laboratories. The SCRAs investigated in this study were extensively metabolized, as the parent compounds were not detected in urine samples analyzed in the context of abstinence control. To address this issue, LC-HESI-HRMS/MS was used for the tentative characterization and identification of phase I and II metabolites of ADMB-3TMS-PrINACA and Cumyl-3TMS-PrINACA in human urine. These findings were verified through in vitro plausibility checks using pHLM and primary hepatocyte incubation with the respective SCRAs. For both 3TMS-based SCRAs, a metabolite monohydroxylated at the *N*-propyl-3TMS side chain (**A1.3**, **C1.2**) was identified as the most abundant and reliable urinary biomarker to prove consumption. Additionally, the authors suggest the *N*-3-OH propyl metabolite (**A8** and **C6**) and the further oxidized *N*-propionic acid metabolite (**A10** and **C8**) as biomarkers for both synthetic cannabinoids. Notably, metabolites C6 and C8 were also generated by incubating Cumyl-PINACA in vitro. Most of the suggested biomarkers of ADMB-3TMS-PrINACA were also found in stomach contents, femoral and heart blood as well as urine obtained from a deceased individual.

The in vitro models, especially the pHLM assays, showed good agreement with the metabolites identified in human urine. GLORYx seems to be more suitable for predicting metabolites of compounds containing a trimethylsilyl propyl moiety compared to BioTransformer. For BioTransformer, a useful strategy to improve prediction for such compounds could be the replacement of the silicon atom by a carbon atom prior to simulation. However, both in silico models are limited in their ability to predict metabolites resulting from 3TMS cleavage or oxidative Si-demethylation.

## Supplementary information

Below is the link to the electronic supplementary material.

Supplementary file1 (DOCX 916 KB)

## Data Availability

Derived data supporting the findings of this study are available from the corresponding author upon request.

## References

[CR1] Abbott MJ, Dunnett J, Wheeler J, Davidson A (2023) The identification of synthetic cannabinoids in English prisons. Forensic Sci Int 348:111613. 10.1016/j.forsciint.2023.111613 36922254 10.1016/j.forsciint.2023.111613

[CR2] Angerer V, Mogler L, Steitz J-P, Bisel P, Hess C, Schoeder CT, Müller CE, Huppertz LM, Westphal F, Schäper J, Auwärter V (2018) Structural characterization and pharmacological evaluation of the new synthetic cannabinoid CUMYL-PEGACLONE. Drug Test Anal 10:597–603. 10.1002/dta.2237 28670781 10.1002/dta.2237

[CR3] Åstrand A, Vikingsson S, Lindstedt D, Thelander G, Gréen H, Kronstrand R, Wohlfarth A (2018) Metabolism study for CUMYL-4CN-BINACA in human hepatocytes and authentic urine specimens: free cyanide is formed during the main metabolic pathway. Drug Test Anal 10:1270–1279. 10.1002/dta.2373 10.1002/dta.2373 29577658

[CR4] Azuma Y, Tanaka M, Asada A, Doi T (2025) In vitro metabolic fate of 1-[3-(trimethylsilyl)propanoyl] lysergic acid diethylamide (1S-LSD), a silicon-containing LSD analog. Forensic Toxicol. 10.1007/s11419-025-00735-2 40730742 10.1007/s11419-025-00735-2

[CR5] Bundesministerium für Gesundheit (2024) Bundesgesetzblatt Teil I—Fünfte Verordnung zur Änderung der Anlage des Neue-psychoaktive-Stoffe-Gesetzes - Bundesgesetzblatt [WWW Document]. https://www.recht.bund.de/bgbl/1/2024/210/VO. Accessed 8 Jan 25

[CR6] Carlier J, Diao X, Scheidweiler KB, Huestis MA (2017) Distinguishing intake of new synthetic cannabinoids ADB-PINACA and 5F-ADB-PINACA with human hepatocyte metabolites and high-resolution mass spectrometry. Clin Chem 63:1008–1021. 10.1373/clinchem.2016.267575 28302730 10.1373/clinchem.2016.267575

[CR7] Cohen K, Weinstein AM (2018) Synthetic and non-synthetic cannabinoid drugs and their adverse effects-a review from public health prospective. Front Public Health 6:162. 10.3389/fpubh.2018.00162 29930934 10.3389/fpubh.2018.00162 PMC5999798

[CR8] Cozier GE, Andrews RC, Frinculescu A, Kumar R, May B, Tooth T, Collins P, Costello A, Haines TSF, Freeman TP, Blagbrough IS, Scott J, Shine T, Sutcliffe OB, Husbands SM, Leach J, Bowman RW, Pudney CR (2023) Instant detection of synthetic cannabinoids on physical matrices, implemented on a low-cost, ultraportable device. Anal Chem 95:13829–13837. 10.1021/acs.analchem.3c01844 37642957 10.1021/acs.analchem.3c01844 PMC10515102

[CR9] de Bruyn Kops C, Šícho M, Mazzolari A, Kirchmair J (2021) GLORYx: prediction of the metabolites resulting from phase 1 and phase 2 biotransformations of xenobiotics. Chem Res Toxicol 34:286–299. 10.1021/acs.chemrestox.0c00224 32786543 10.1021/acs.chemrestox.0c00224 PMC7887798

[CR10] Di Trana A, Brunetti P, Giorgetti R, Marinelli E, Zaami S, Busardò FP, Carlier J (2021) In silico prediction, LC-HRMS/MS analysis, and targeted/untargeted data-mining workflow for the profiling of phenylfentanyl in vitro metabolites. Talanta 235:122740. 10.1016/j.talanta.2021.122740 34517608 10.1016/j.talanta.2021.122740

[CR11] Diao X, Huestis MA (2019) New synthetic cannabinoids metabolism and strategies to best identify optimal marker metabolites. Front Chem. 10.3389/fchem.2019.00109 30886845 10.3389/fchem.2019.00109 PMC6409358

[CR12] Duan W, Sun Y, Wu M, Zhang Z, Zhang T, Wang H, Li F, Yang L, Xu Y, Liu Z-J, Hua T, Nie H, Cheng J (2021) Carbon-silicon switch led to the discovery of novel synthetic cannabinoids with therapeutic effects in a mouse model of multiple sclerosis. Eur J Med Chem 226:113878. 10.1016/j.ejmech.2021.113878 34634742 10.1016/j.ejmech.2021.113878

[CR13] EUDA (2025a) EDND: formal notification ADMB-3TMS-PrINACA. European Union Drugs Agency [WWW Document]. https://ednd2.emcdda.europa.eu/ednd/substanceProfiles/1396. Accessed 30 July 2025

[CR14] EUDA (2025b) EDND: Formal Notification Cumyl-3TMS-PrINACA. European Union Drugs Agency [WWW Document]. https://ednd2.emcdda.europa.eu/ednd/substanceProfiles/1402. Accessed 30 July 2025

[CR15] Fotie J, Matherne CM, Wroblewski JE (2023) Silicon switch: carbon–silicon bioisosteric replacement as a strategy to modulate the selectivity, physicochemical, and drug-like properties in anticancer pharmacophores. Chem Biol Drug Design 102:235–254. 10.1111/cbdd.14239 10.1111/cbdd.14239 37029092

[CR16] Giorgetti A, Amurri S, Fazio G, Bini C, Anniballi L, Pirani F, Pelletti G, Pelotti S (2023) The evaluation of CYP2D6, CYP2C9, CYP2C19, and CYP2B6 phenoconversion in post-mortem casework: the challenge of forensic toxicogenetics. Metabolites 13:661. 10.3390/metabo13050661 37233702 10.3390/metabo13050661 PMC10221100

[CR17] Giorgetti A, Zschiesche A, Groth O, Haschimi B, Scheu M, Pelletti G, Fais P, Musshoff F, Auwärter V (2024) ADB-HEXINACA—a novel synthetic cannabinoid with a hexyl substituent: phase I metabolism in authentic urine samples, a case report and prevalence on the German market. Drug Test Anal 16:1350–1365. 10.1002/dta.3657 38350637 10.1002/dta.3657

[CR18] Grafinger KE, Vandeputte MM, Cannaert A, Ametovski A, Sparkes E, Cairns E, Juchli PO, Haschimi B, Pulver B, Banister SD, Stove CP, Auwärter V (2021) Systematic evaluation of a panel of 30 synthetic cannabinoid receptor agonists structurally related to MMB-4en-PICA, MDMB-4en-PINACA, ADB-4en-PINACA, and MMB-4CN-BUTINACA using a combination of binding and different CB1 receptor activation assays. Part III: the G protein pathway and critical comparison of different assays. Drug Test Anal 13:1412–1429. 10.1002/dta.3054 33908179 10.1002/dta.3054

[CR19] Harvey DJ, Vouros P (2020) Mass spectrometric fragmentation of trimethylsilyl and related alkylsilyl derivatives. Mass Spectrom Rev 39:105–211. 10.1002/mas.21590 31808199 10.1002/mas.21590

[CR20] Holm NB, Noble C, Linnet K (2016) JWH-018 ω-OH, a shared hydroxy metabolite of the two synthetic cannabinoids JWH-018 and AM-2201, undergoes oxidation by alcohol dehydrogenase and aldehyde dehydrogenase enzymes in vitro forming the carboxylic acid metabolite. Toxicol Lett 259:35–43. 10.1016/j.toxlet.2016.07.007 27421777 10.1016/j.toxlet.2016.07.007

[CR21] Huskey SW, Dean DC, Miller RR, Rasmusson GH, Chiu SH (1995) Identification of human cytochrome P450 isozymes responsible for the in vitro oxidative metabolism of finasteride. Drug Metab Dispos 23:1126 8654202

[CR22] Hutter M, Moosmann B, Kneisel S, Auwärter V (2013) Characteristics of the designer drug and synthetic cannabinoid receptor agonist AM-2201 regarding its chemistry and metabolism. J Mass Spectrom 48:885–894. 10.1002/jms.3229 23832945 10.1002/jms.3229

[CR23] Jiang W, Wilson MA, Weeks DP (2013) O-Demethylations catalyzed by rieske nonheme iron monooxygenases involve the difficult oxidation of a saturated C-H bond. ACS Chem Biol 8:1687–1691. 10.1021/cb400154a 23719540 10.1021/cb400154a

[CR24] Kavanagh P, Pechnikov A, Nikolaev I, Dowling G, Kolosova M, Grigoryev A (2022) Detection of ADB-BUTINACA metabolites in human urine, blood, kidney and liver. J Anal Toxicol 46:641–650. 10.1093/jat/bkab088 34341821 10.1093/jat/bkab088

[CR25] Kirchmair J, Göller AH, Lang D, Kunze J, Testa B, Wilson ID, Glen RC, Schneider G (2015) Predicting drug metabolism: experiment and/or computation? Nat Rev Drug Discov 14:387–404. 10.1038/nrd4581 25907346 10.1038/nrd4581

[CR26] Liu C-M, Jia W, Meng X, Hua Z-D (2021) Identification and quantification of 10 indole/indazole carboxamide synthetic cannabinoids in 36 herbal blends by gas chromatography-mass spectrometry and nuclear magnetic resonance spectroscopy. J Forensic Sci 66:2156–2166. 10.1111/1556-4029.14873 34431514 10.1111/1556-4029.14873

[CR27] Lundahl A, Lennernäs H, Knutson L, Bondesson U, Hedeland M (2009) Identification of finasteride metabolites in human bile and urine by high-performance liquid chromatography/tandem mass spectrometry. Drug Metab Dispos 37:2008–2017. 10.1124/dmd.109.027870 19635781 10.1124/dmd.109.027870

[CR28] Luo X, Zhang J, Huang K, Liu X, Yang N, Li J, Luo Q (2024) Investigation of electron ionization mass spectrometric fragmentation pattern of indole- and indazole-type synthetic cannabinoids. Forensic Chem 37:100557. 10.1016/j.forc.2024.100557

[CR29] Moosmann B, Kneisel S, Girreser U, Brecht V, Westphal F, Auwärter V (2012) Separation and structural characterization of the synthetic cannabinoids JWH-412 and 1-[(5-fluoropentyl)-1H-indol-3yl]-(4-methylnaphthalen-1-yl)methanone using GC–MS, NMR analysis and a flash chromatography system. Forensic Sci Int 220:e17–e22. 10.1016/j.forsciint.2011.12.010 22264627 10.1016/j.forsciint.2011.12.010

[CR30] Musshoff F, Stamer UM, Madea B (2010) Pharmacogenetics and forensic toxicology. Forensic Sci Int 203:53–62. 10.1016/j.forsciint.2010.07.011 20828952 10.1016/j.forsciint.2010.07.011

[CR31] Navarro-Tapia E, Codina J, Villanueva-Blasco VJ, García-Algar Ó, Andreu-Fernández V (2022) Detection of the synthetic cannabinoids AB-CHMINACA, ADB-CHMINACA, MDMB-CHMICA, and 5F-MDMB-PINACA in biological matrices: a systematic review. Biology 11:796. 10.3390/biology11050796 35625524 10.3390/biology11050796 PMC9139075

[CR32] Norman C, Walker G, McKirdy B, McDonald C, Fletcher D, Antonides LH, Sutcliffe OB, Nic Daéid N, McKenzie C (2020) Detection and quantitation of synthetic cannabinoid receptor agonists in infused papers from prisons in a constantly evolving illicit market. Drug Test Anal 12:538–554. 10.1002/dta.2767 31944624 10.1002/dta.2767

[CR33] Open-Mind Market (2024) 1S LSD Blotter 150mcg—Open-Mind Market. 1S LSD Blotter 150 mcg. https://openmind.market/produkt/1s-lsd-blotter-150mcg/. Accessed 24 June 2024

[CR34] Öztürk YE, Yeter O, Öztürk S, Karakus G, Ates I, Buyuk Y, Yurdun T (2018) Detection of metabolites of the new synthetic cannabinoid CUMYL-4CN-BINACA in authentic urine samples and human liver microsomes using high-resolution mass spectrometry. Drug Test Anal 10:449–459. 10.1002/dta.2248 28691766 10.1002/dta.2248

[CR35] Panayides J-L, Lyall Riley D, Hasenmaile F, van Otterlo WAL (2024) The role of silicon in drug discovery: a review. RSC Med Chem. 10.1039/D4MD00169A 39430101 10.1039/d4md00169a PMC11484438

[CR36] Pelletier R, Nahle D, Sarr M, Bourdais A, Morel I, Le Daré B, Gicquel T (2025) Identifying metabolites of new psychoactive substances using in silico prediction tools. Arch Toxicol 99:2953–2973. 10.1007/s00204-025-04049-5 40358677 10.1007/s00204-025-04049-5 PMC12198081

[CR37] Sarai NS, Fulton TJ, O’Meara RL, Johnston KE, Brinkmann-Chen S, Maar RR, Tecklenburg RE, Roberts JM, Reddel JCT, Katsoulis DE, Arnold FH (2024) Directed evolution of enzymatic silicon-carbon bond cleavage in siloxanes. Science 383:438–443. 10.1126/science.adi5554 38271505 10.1126/science.adi5554

[CR38] Scholz V-A, Stork C, Frericks M, Kirchmair J (2023) Computational prediction of the metabolites of agrochemicals formed in rats. Sci Total Environ 895:165039. 10.1016/j.scitotenv.2023.165039 37355108 10.1016/j.scitotenv.2023.165039

[CR39] Schwartz MD, Trecki J, Edison LA, Steck AR, Arnold JK, Gerona RR (2015) A common source outbreak of severe delirium associated with exposure to the novel synthetic cannabinoid ADB-PINACA. J Emerg Med 48:573–580. 10.1016/j.jemermed.2014.12.038 25726258 10.1016/j.jemermed.2014.12.038 PMC9049074

[CR40] Shen J, Serby M, Surber B, Lee AJ, Ma J, Badri P, Menon R, Kavetskaia O, Morais SMde, Sydor J, Fischer V (2016) Metabolism and disposition of pan-genotypic inhibitor of Hepatitis C virus NS5A ombitasvir in humans. Drug Metab Dispos 44:1148–1157. 10.1124/dmd.115.067496 27179128 10.1124/dmd.115.067496

[CR41] Sia CH, Wang Z, Goh EML, Tan YL, Fong CY, Moy HY, Chan ECY (2021) Urinary metabolite biomarkers for the detection of synthetic cannabinoid ADB-BUTINACA abuse. Clin Chem 67:1534–1544. 10.1093/clinchem/hvab134 34387654 10.1093/clinchem/hvab134

[CR42] Stork C, Embruch G, Šícho M, de Bruyn Kops C, Chen Y, Svozil D, Kirchmair J (2020) NERDD: a web portal providing access to in silico tools for drug discovery. Bioinformatics 36:1291–1292. 10.1093/bioinformatics/btz695 32077475 10.1093/bioinformatics/btz695

[CR43] Tacke R, Dörrich S (2016) Drug design based on the carbon/silicon switch strategy. In: Schwarz J (ed) Atypical elements in drug design, topics in medicinal chemistry. Springer International Publishing, Cham, pp 29–59. 10.1007/7355_2014_55

[CR44] Tai S, Fantegrossi WE (2014) Synthetic cannabinoids: pharmacology, behavioral effects, and abuse potential. Curr Addict Rep 1:129–136. 10.1007/s40429-014-0014-y 26413452 10.1007/s40429-014-0014-y PMC4582439

[CR45] Tanaka R, Kawamura M, Ito M, Kikura-Hanajiri R (2025) Identification of two lysergic acid diethylamide analogs, 1-(3-(trimethylsilyl) propionyl) lysergic acid diethylamide (1S-LSD) and 1-(2-thienoyl)-6-allyl-nor-d-lysergic acid diethylamide (1T-AL-LAD), in paper sheet products distributed on the internet. Forensic Toxicol. 10.1007/s11419-025-00718-3 40180768 10.1007/s11419-025-00718-3

[CR46] van Amsterdam J, Brunt T, van den Brink W (2015) The adverse health effects of synthetic cannabinoids with emphasis on psychosis-like effects. J Psychopharmacol 29:254–263. 10.1177/0269881114565142 25586398 10.1177/0269881114565142

[CR47] Wishart DS, Tian S, Allen D, Oler E, Peters H, Lui VW, Gautam V, Djoumbou-Feunang Y, Greiner R, Metz TO (2022) BioTransformer 3.0—a web server for accurately predicting metabolic transformation products. Nucleic Acids Res 50:W115–W123. 10.1093/nar/gkac313 35536252 10.1093/nar/gkac313 PMC9252798

[CR48] Zschiesche A, Scheu M, Thieme D, Keiler AM, Pulver B, Huppertz LM, Auwärter V (2024) Insights into the metabolism of CH-PIATA—a novel synthetic cannabinoid featuring an acetamide linker. J Anal Toxicol. 10.1093/jat/bkae013 38441323 10.1093/jat/bkae013

