# Supplementary material for: Synthetic cannabinoid receptor agonists containing silicon: exploring the metabolic pathways of ADMB- and Cumyl-3TMS-PrINACA in human urine specimens and post mortem material compared to in vitro and in silico data
Source: Arch Toxicol. 2025 Oct 7;100(2):525–42. doi: 10.1007/s00204-025-04204-y (PMC12886380; doi:10.1007/s00204-025-04204-y)
Supplement: Supplementary file 1 — Supplementary file1 (DOCX 916 KB) [file 204_2025_4204_MOESM1_ESM.docx]

***Supplementary Information***

**Synthetic Cannabinoid Receptor Agonists Containing Silicon: Exploring the Metabolic Pathways of ADMB- and Cumyl-3TMS-PrINACA in Human Urine Specimens Compared to *In Vitro* and *In Silico* Data**

**Annette Zschiesche^1,2^, Jeremy Carlier^3^, Jörg Pietsch^4^, Martin Scheu^1,2^, Jasmin Seibt^4^, Francesco P. Busardò^3^, Volker Auwärter^1^ and Laura M. Huppertz^1^***

^1^Institute of Forensic Medicine, Forensic Toxicology, Medical Center and Faculty of Medicine, University of Freiburg, Albertstr. 9, Freiburg 79104, Germany

^2^Hermann Staudinger Graduate School, University of Freiburg, Hebelstr. 27, 79104 Freiburg, Germany

^3^Section of Legal Medicine, Department of Biomedical Sciences and Public Health, Marche Polytechnic University, via Tronto 10/a, 60126, Ancona, Italy

^4^Institute of Legal Medicine, Medical Faculty Carl Gustav Carus, Dresden Technical University, Fetscherstr. 74, 01307, Dresden, Germany

***** Correspondence: [laura.huppertz@uniklinik-freiburg.de](mailto:laura.huppertz@uniklinik-freiburg.de)

Journal name: Archives of Toxicology

**Table S1** Inclusion list for LC-QToF-MS/MS acquisition for ADMB-, Cumyl-3TMS-PrINACA and their metabolites with quadrupole mass selection: ±0.5 Da

| **Parent compound and metabolites** | **[M+H]^+^ *m/z*** |
| --- | --- |
| **ADMB-3TMS-PrINACA** | 389.2367 |
| Monohydroxylation | 405.2316 |
| Dehydration | 387.2211 |
| Ketone/aldehyde | 403.2160 |
| Hydrolysis | 390.2207 |
| Hydroxylation+ Hydrolysis | 406.2157 |
| Dihydroxylation | 421.2266 |
| *N*-Dealkylation | 275.1503 |
| *N*-Dealkylation + hydroxylation | 291.1450 |
| Dihydrodiole | 423.2422 |
| -3TMS+ hydroxylation | 333.1921 |
| -3TMS+ carboxylic acid | 347.1714 |
| -3TMS+ aldehyde | 331.1761 |
| -3TMS+ hydroxylation + hydrolysis | 334.1761 |
| -3TMS+ carboxylic acid + hydrolysis | 348.1554 |
| Triole | 437.2214 |
| Acid formation | 419.2101 |
| Demethylation | 375.2211 |
| 2xDemethylation | 361.2054 |
| Demethylation + hydroxylation | 391.2160 |
| Demethylation + dihydroxylation | 407.2109 |
| Monohydroxylation + glucuronic acid | 581.2637 |
| -3TMS+ hydroxylation + glucuronic acid | 509.2242 |
| -3TMS+ carboxylic acid + glucuronic acid | 523.2035 |
| Dihydroxylation + glucuronic acid | 597.2586 |
| Monohydroxylation + sulfate | 485.1885 |
| **Cumyl-3TMS-PrINACA** | 394.2309 |
| Monohydroxylation | 410.2258 |
| Dehydration | 392.2153 |
| Ketone/aldehyde | 408.2102 |
| Dihydroxylation | 426.2207 |
| *N*-Dealkylation | 280.1444 |
| *N*-Dealkylation + hydroxylation | 296.1394 |
| Dihydrodiole | 428.2364 |
| -3TMS+ hydroxylation | 338.1863 |
| -3TMS+ carboxylic acid | 352.1656 |
| -3TMS+ aldehyde | 336.1707 |
| Triole | 442.2157 |
| O-Methylation | 422.2269 |
| Acid formation | 424.2051 |
| Demethylation | 380.2153 |
| 2xDemethylation | 366.1996 |
| -3TMS+ dihydroxylation | 354.1817 |
| Demethylation + hydroxylation | 396.2102 |
| Demethylation + dihydroxylation | 412.2051 |
| Monohydroxylation + glucuronic acid | 586.2579 |
| -3TMS+ hydroxylation + glucuronic acid | 514.2184 |
| -3TMS+ carboxylic acid + glucuronic acid | 528.1977 |
| Dihydroxylation + glucuronic acid | 602.2383 |
| Monohydroxylation + sulfate | 490.1826 |

**Table S2** Metabolic transformation, elemental composition, retention time (t_R_), accurate mass of molecular ion in positive ionization modes ([M+H]^+^) with mass error, diagnostic product ions, and *in vivo* and *in vitro* mean area ratios (MAR, [%]) based on relative peak areas of LC-QToF-MS analysis for phase I metabolites of ADMB-3TMS-PrINACA. Only hydrolyzed urines and PHH supernatants are shown. The three most abundant phase I metabolites found in urine samples are highlighted in **bold.** The parent substance was excluded from the MAR calculation

| **ID** | **t_R_ (min)** | **Metabolic Transformation (Location)** | **Elemental Composition** | **Calculated *m/z* [M+H]^+^ (Δppm)** | **Diagnostic Product Ions *m/z*** | **MAR *In Vivo* [%]** | **MAR *In Vitro* [%]** | |
| --- | --- | --- | --- | --- | --- | --- | --- | --- |
|  |  |  |  |  |  | **Urines** | **pHLMs** | **PHH** |
| A1.1 | 5.80 | Hydroxylation (S) | C_20_H_32_N_4_O_3_Si | 405.2316 (2.5) | 230.1288; 275.1210; 187.0866 | 7.34% | 15.63% | □ |
| **A1.2** | **7.02** | **Hydroxylation (S)** | **C_20_H_32_N_4_O_3_Si** | **405.2316 (1.6)** | **275.1210; 293.1316; 360.2102** | 78.37% | 54.03% | 33.98% |
| **A1.3** | **7.96** | **Hydroxylation (S)** | **C_20_H_32_N_4_O_3_Si** | **405.2316 (1.1)** | **275.1210; 293.1316; 360.2102** | 100.00% | 100.00% | 100.00% |
| A1.4 | 8.23 | Hydroxylation (S) | C_20_H_32_N_4_O_3_Si | 405.2316 (2.1) | 275.1210; 293.1316; 360.2102 | 7.60% | 23.47% | 9.59% |
| A1.5 | 9.22 | Hydroxylation (S) | C_20_H_32_N_4_O_3_Si | 405.2316 (2.3) | 275.1210; 360.2102; 342.1996 | 7.17% | 4.52% | 1.37% |
| A1.6 | 9.56 | Hydroxylation (S) | C_20_H_32_N_4_O_3_Si | 405.2316 (2.0) | 275.1210; 360.2102; 342.1996 | 7.66% | 7.95% | 2.80% |
| A1.7 | 10.55 | Hydroxylation (ADMB) | C_20_H_32_N_4_O_3_Si | 405.2316 (1.2) | 259.1261; 330.1990; 73.0468 | 4.41% | 26.30% | 7.09% |
| A1.8 | 10.79 | Hydroxylation (IN) | C_20_H_32_N_4_O_3_Si | 405.2316 (1.7) | 275.1210; 360.2102; 161.0346 | 4.82% | 4.81% | 1.98% |
| A2 | 14.32 | Hydrolysis (ADMB) | C_20_H_31_N_3_O_3_Si | 390.2207 (0.2) | 259.1262; 231.0948; 73.0468 | 5.64% | 0.25% | 3.47% |
| **A3.1** | **4.34** | **Dihydroxylation (2xS)** | **C_20_H_32_N_4_O_4_Si** | **421.2266 (0.4)** | **376.2051; 291.1159; 273.1054** | 57.96% | 13.59% | 1.94% |
| A3.2 | 5.08 | Dihydroxylation (2xS) | C_20_H_32_N_4_O_4_Si | 421.2266 (-1.6) | 275.121; 291.1159; 145.0396 | 15.35% | 6.21% | 1.46% |
| A3.3 | 5.34 | Dihydroxylation (2xS) | C_20_H_32_N_4_O_4_Si | 421.2266 (0.1) | 273.1054; 291.1159; 145.0396 | 4.26% | 1.44% | 0.44% |
| A3.4 | 5.67 | Dihydroxylation (S+ADMB) | C_20_H_32_N_4_O_4_Si | 421.2266 (1.2) | 275.121; 346.1945; 145.0396 | 18.71% | 12.95% | 2.39% |
| A4.1 | 8.45 | Hydrolysis (ADMB) + Hydroxylation (S) | C_20_H_31_N_3_O_4_Si | 406.2157 (-1.3) | 275.1210; 360.2102; 145.0396 | 31.21% | 0.05% | 0.76% |
| A4.2 | 9.38 | Hydrolysis (ADMB) + Hydroxylation (S) | C_20_H_31_N_3_O_4_Si | 406.2157 (-0.1) | 275.1210; 360.2102; 145.0396 | 37.34% | 0.31% | 2.41% |
| A5 | 10.38 | Ketone/aldehyde (ADMB) | C_20_H_31_N_4_O_3_Si | 403.216 (0.4) | 259.1262; 231.0948; 185.1073 | 0.76% | 10.48% | 2.42% |
| A6 | 5.11 | Acid formation (S) | C_20_H_30_N_4_O_4_Si | 419.2109 (1.4) | 275.1210; 401.2003 | 8.56% | 0.49% | 0.47% |
| A7 | 6.31 | Demethylation + hydroxylation (S) | C_19_H_29_N_4_O_3_Si | 391.2160 (3.2) | 261.1054; 346.1945; | 17.34% | 27.81% | 5.66% |
| A8 | 2.91 | Cleavage 3TMS + terminal hydroxylation (S) | C_17_H_24_N_4_O_3_ | 333.1921 (-1.0) | 203.0815; 220.1081; 288.1707 | 48.39% | 26.65% | 9.68% |
| A9 | 3.45 | Cleavage 3TMS + terminal aldehyde (S) | C_17_H_22_N_4_O_3_ | 331.1764 (-4.9) | 201.0659; 286.1550; 145.0396 | □ | 0.38% | 0.32% |
| A10 | 2.97 | Cleavage 3TMS + carboxylic acid formation (S) | C_17_H_22_N_4_O_4_ | 347.1714 (0.5) | 217.0608, 234.0873; 302.1499 | 53.27% | 0.99% | 0.90% |
| A11 | 2.69 | *N*-Dealkylation (ADMB-INACA) | C_14_H_18_N_4_O_2_ | 275.1503 (-1.0) | 145.0396; 230.1279; 258.1237 | 2.11% | 1.33% | 0.71% |
| A12.1 | 3.21 | Demethylation (S) + Dihydroxylation (2xS) | C_19_H_30_N_4_O_4_Si | 407.2109 (-2.1) | 259.0897; 277.1003; 145.0396 | 2.47% | 0.99% | 0.01% |
| A12.2 | 3.63 | Demethylation (S) + Dihydroxylation (2xS) | C_19_H_30_N_4_O_4_Si | 407.2109 (-0.2) | 259.0897; 277.1003; 145.0396 | 41.73% | 1.46% | 0.24% |
| A12.3 | 3.78 | Demethylation (S) + Dihydroxylation (2xS) | C_19_H_30_N_4_O_4_Si | 407.2109 (-1.8) | 259.0897; 277.1003; 145.0396 | 3.14% | 0.37% | 0.08% |
| A12.4 | 4.11 | Demethylation (S) + Dihydroxylation (S+IN) | C_19_H_30_N_4_O_4_Si | 407.2109 (-1.1) | 277.1003; 161.0346 | 4.13% | 0.91% | 0.04% |
| A12.5 | 4.52 | Demethylation (S) + Dihydroxylation (S+ADMB) | C_19_H_30_N_4_O_4_Si | 407.2109 (-1.0) | 261.1054; 145.0396 | 36.51% | 2.14% | 0.30% |
| A0 | 13.10 | ADMB-3TMS-PrINACA (Parent) | C_20_H_32_N_4_O_2_Si | 389.2367 (0.5) | 259.1261; 344.2153; 73.0468 | □ | ■ | ■ |

t_R_: retention time, IN: indazole; S: side chain (3TMS-PrINACA), pHLM: pooled liver microsomes, PHH: primary human hepatocytes, ■: detected but not included in MAR, □: not detected


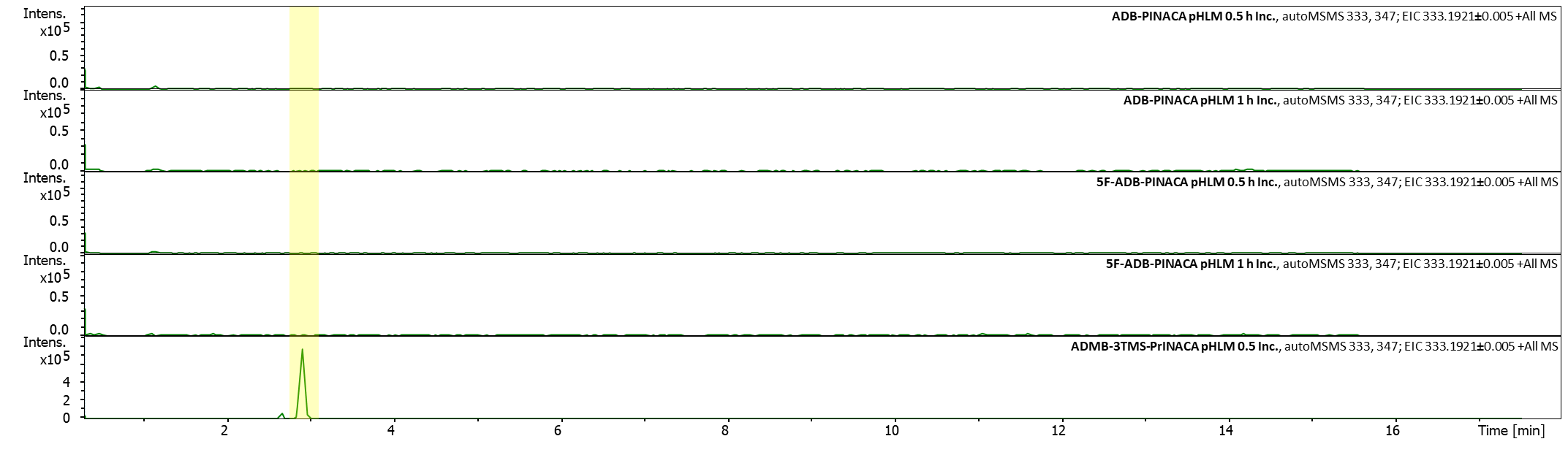


**Figure S1** Metabolite A8 (ADMB-3TMS-PrINACA -3TMS + OH, *m/z* = 333.1921) measured in means of LC-HR-MS/MS in positive autoMS/MS mode in pHLM incubations of ADB-PINACA (incubation time: 0.5 and 1 h), 5F-ADB-PINACA (incubation time: 0.5 and 1 h), and ADMB-3TMS-PrINACA incubation time: 0.5 h)


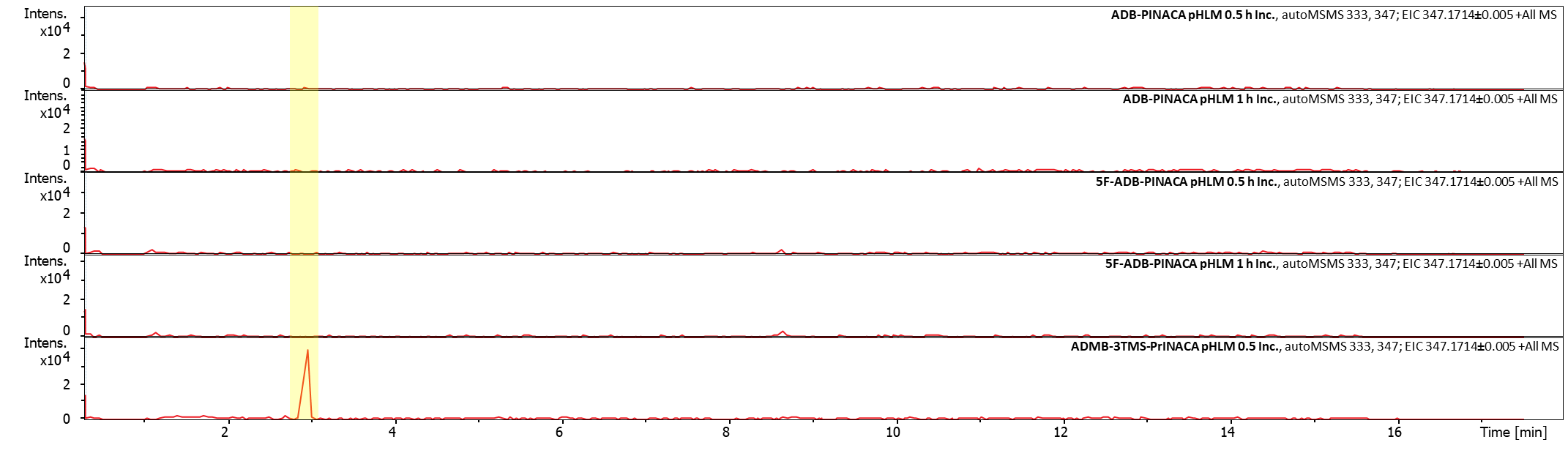


**Fig. S2** Metabolite A10 (ADMB-3TMS-PrINACA -3TMS + COOH, *m/z* = 347.1714) measured in means of LC-HR-MS/MS in positive autoMS/MS mode in pHLM incubations of ADB-PINACA (incubation time: 0.5 and 1 h), 5F-ADB-PINACA (incubation time: 0.5 and 1 h), and ADMB-3TMS-PrINACA incubation time: 0.5 h)

**Table S3** Phase II metabolites of ADMB-3TMS-PrINACA with retention times, structural modifications, exact masses of the protonated molecules, elemental composition, mass errors, and major and characteristic fragment ions. Filled boxes: confirmation, Empty boxes: no confirmation

| **ID** | **t_R_ [min]** | **Modification (Location)** | **Elemental Composition** | **Calculated *m/z* [M+H]^+^ (Δppm)** | **Diagnostic Ions *m/z*** | **Urine** | **PHH** |
| --- | --- | --- | --- | --- | --- | --- | --- |
| **A I** | **5.11** | **Hydroxylation (S) + glucuronidation** | **C_26_H_40_N_4_O_9_Si** | **581.2637 (1.68)** | **275.1210; 360.2102; 388.2051** | **■** | **■** |
| A II | 5.32 | Hydroxylation (S) + glucuronidation | C_26_H_40_N_4_O_9_Si | 581.2637 (1.87) | 275.1210; 360.2102; 388.2051 | ■ | □ |
| A III | 5.48 | Hydroxylation (S) + glucuronidation | C_26_H_40_N_4_O_9_Si | 581.2637 (1.87) | 275.1210; 360.2102; 388.2051 | ■ | □ |
| A IV | 5.76 | Hydroxylation (S) + glucuronidation | C_26_H_40_N_4_O_9_Si | 581.2637 (1.92) | 275.1210; 360.2102; 388.2051 | ■ | ■ |
| A V | 7.51 | Hydroxylation (ADMB) + glucuronidation | C_26_H_40_N_4_O_9_Si | 581.2637 (1.48) | 259.1261; 344.2153; 388.2051 | ■ | ■ |
| A VI | 1.67 | Cleavage 3TMS + terminal hydroxylation (S) + glucuronidation (S) | C_23_H_32_N_4_O_9_ | 509.2242 (-1.61) | 203.0815; 288.1707 | ■ | □ |
| A VII | 1.75 | Cleavage 3TMS + carboxylic acid formation (S) + glucuronidation (S) | C_23_H_30_N_4_O_10_ | 523.2035 (0.68) | 217.0608; 302.1499 | ■ | □ |

t_R_: retention time, S: side chain (3TMS-PrINACA), ADMB: *N*-(1-Aminocarbonyl-2,2-dimethylpropyl), PHH: primary human hepatocytes

**Table S4** Metabolic transformation, elemental composition, retention time (t_R_), accurate mass of molecular ion in positive ionization modes ([M+H]^+^) with mass error, diagnostic product ions, and *in vivo* and *in vitro* mean area ratios (MAR, [%]) based on relative peak areas of LC-QToF-MS analysis for phase I metabolites of Cumyl-3TMS-PrINACA. Only hydrolyzed urines and PHH supernatants are shown. The three most abundant phase I metabolites found in urine samples are highlighted in **bold.** The parent substance was excluded from the MAR calculation

| **ID** | **t_R_ (min)** | **Metabolic Transformation (Location)** | **Elemental Composition** | **Calculated *m/z* [M+H]^+^ (Δppm)** | **Diagnostic Product Ions *m/z*** | **MAR *In Vivo* [%]** | **MAR *In Vitro* [%]** | |
| --- | --- | --- | --- | --- | --- | --- | --- | --- |
|  |  |  |  |  |  | **Urine** | **pHLMs** | **PHH** |
| C1.1 | 12.06 | Hydroxylation (S) | C_23_H_31_N_3_O_2_Si | 410.2258 (-0.43) | 275.1210; 292.1476; 187.0866; 119.0855 | 10.18% | 3.85% | 18.05% |
| **C1.2** | **13.18** | **Hydroxylation (S)** | **C_23_H_31_N_3_O_2_Si** | **410.2258 (-0.58)** | **275.1210; 119.0855; 145.0396** | **100.00%** | 74.36% | 70.84% |
| C1.3 | 14.20 | Hydroxylation (S) | C_23_H_31_N_3_O_2_Si | 410.2258 (0.70) | 119.0855; 275.1210; 292.1476 | 24.36% | 26.65% | 38.0% |
| C1.4 | 14.76 | Hydroxylation (Cumyl) | C_23_H_31_N_3_O_2_Si | 410.2258 (-1.79) | 135.0804; 259.1261; 231.1312 | 0.09% | 2.94% | 0.00% |
| C1.5 | 14.86 | Hydroxylation (Cumyl) | C_23_H_31_N_3_O_2_Si | 410.2258 (-1.24) | 259.1261; 231.1312; 135.0804 | 0.35% | 3.89% | 1.27% |
| C1.6 | 15.55 | Hydroxylation (Cumyl) | C_23_H_31_N_3_O_3_Si | 410.2258 (-0.52) | 259.1261; 231.1312; 135.0804 | □ | 0.99% | □ |
| C2 | 15.95 | Dehydrogenation (S) | C_23_H_29_N_3_OSi | 392.2153 (-1.48) | 257.1105; 274.1370; 119.0855 | □ | 3.40% | □ |
| C3.1 | 8.58 | Dihydroxylation (2xS) | C_23_H_31_N_3_O_3_Si | 426.2207 (-0.60) | 119.0855; 273.1054; 290.1319; 308.1425 | 3.23% | 3.52% | 4.39% |
| C3.2 | 9.65 | Dihydroxylation (2xS) | C_23_H_31_N_3_O_3_Si | 426.2207 (-0.71) | 119.0855; 185.0709; 273.1054; 290.1319 | 4.02% | 3.84% | 2.75% |
| C3.3 | 9.92 | Dihydroxylation (S+Cumyl) | C_23_H_31_N_3_O_3_Si | 426.2207 (-0.76) | 135.0804; 275.1210; 292.1476 | 2.23% | 0.99% | 0.58% |
| C3.4 | 10.14 | Dihydroxylation (S+Cumyl) | C_23_H_31_N_3_O_3_Si | 426.2207 (-1.05) | 135.0804; 275.1210; 292.1476 | 4.04% | 2.24% | 1.24% |
| C3.5 | 10.85 | Dihydroxylation (2xS) | C_23_H_31_N_3_O_3_Si | 426.2207 (-1.72) | 119.0855; 185.0709; 273.1054; 290.1319 | 1.39% | 1.81% | 0.85% |
| C3.6 | 10.98 | Dihydroxylation (2xS) | C_23_H_31_N_3_O_3_Si | 426.2207 (-0.95) | 119.0855; 273.1054; 290.1319 | 1.19% | 0.78% | 0.19% |
| C3.7 | 11.17 | Dihydroxylation (S+Cumyl) | C_23_H_31_N_3_O_3_Si | 426.2207 (-1.29) | 135.0804; 275.1210; 292.1476 | 2.67% | 0.61% | 0.28% |
| C3.8 | 13.44 | Dihydroxylation (2xCumyl) | C_23_H_31_N_3_O_3_Si | 426.2207 (-1.42) | 151.0754; 259.1261; 276.1527 | 0.01% | 0.48% | 0.06% |
| C4.1 | 14.11 | Ketone/aldehyde (S) | C_23_H_29_N_3_O_2_Si | 408.2102 (-1.94) | 273.1054; 290.1319; 73.0468 | □ | 1.42% | □ |
| C4.2 | 15.88 | Ketone/aldehyde (Cumyl) | C_23_H_29_N_3_O_2_Si | 408.2102 (0.05) | 259.1261; 231.1312; 133.0648 | □ | 0.86% | □ |
| C5 | 11.44 | Demethylation + hydroxylation (S) | C_22_H_29_N_3_O_2_Si | 396.2102 (-1.27) | 261.1054; 278.1319; 119.0855 | 1.78% | 22.26% | 21.30% |
| **C6** | **6.92** | **Cleavage 3TMS + terminal hydroxylation (S)** | **C_20_H_23_N_3_O_2_** | **338.1863 (0.26)** | **203.0815; 119.0855; 145.0396** | **75.48%** | 100.0% | 100.0% |
| C7 | 8.17 | Cleavage 3TMS + terminal aldehyde formation (S) | C_20_H_21_N_3_O_2_ | 336.1707 (-0.42) | 201.0658; 119.0855 | □ | 34.14% | 13.70% |
| **C8** | **6.87** | **Cleavage 3TMS + carboxylic acid formation (S)** | **C_20_H_21_N_3_O_3_** | **352.1656 (0.06)** | **217.0608; 119.0855; 145.0396** | **70.66%** | 32.83% | 59.30% |
| C9.1 | 8.42 | Triol (S + 2xCumyl) | C_23_H_31_N_3_O_4_Si | 442.2157 (-0.88) | 275.1210; 151.0754; 291.1159 | 0.16% | 1.10% | □ |
| C9.2 | 9.74 | Triol (S + 2xCumyl) | C_23_H_31_N_3_O_4_Si | 442.2157 (-0.50) | 151.0754; 291.1159 | □ | 0.37% | □ |
| C10.1 | 4.38 | Cleavage 3TMS + terminal hydroxylation (S) + hydroxylation (Cumyl) | C_20_H_23_N_3_O_3_ | 354.1817 (0.88) | 203.0815; 135.0804; 220.1081 | 0.06% | 1.74% | □ |
| C10.2 | 4.6 | Cleavage 3TMS + terminal hydroxylation (S) + hydroxylation (Cumyl) | C_20_H_23_N_3_O_3_ | 354.1817 (1.03) | 203.0815; 135.0804; 220.1081 | 2.97% | 3.06% | 1.93% |
| C10.3 | 5.07 | Cleavage 3TMS + terminal hydroxylation (S) + hydroxylation (S) | C_20_H_23_N_3_O_3_ | 354.1817 (0.77) | 219.0764; 119.0855; 236.1030 | 4.04% | 2.24% | 1.24% |
| C10.4 | 5.89 | Cleavage 3TMS + terminal hydroxylation (S) + hydroxylation (IN) | C_20_H_23_N_3_O_3_ | 354.1817 (1.49) | 219.0764; 161.0351; 236.1030 | 0.41% | 1.86% | 0.17% |
| C11.1 | 7.66 | Demethylation (S) + dihydroxylation (2xS) | C_22_H_29_N_3_O_3_Si | 412.2051 (-0.51) | 259.0897; 277.1003; 119.0855 | 16.74% | 19.25% | 26.37% |
| C11.2 | 8.27 | Demethylation (S) + dihydroxylation (2xS) | C_22_H_29_N_3_O_3_Si | 412.2051 (-1.75) | 119.0855; 258.1057; 276.1165 | 15.25% | 22.04% | 12.37% |
| C12 | 7.09 | *N*-Dealkylation (Cumyl-INACA) | C_17_H_17_N_3_O | 280.1444 (-1.43) | 145.0396; 119.0855; 91.054 | 0.04% | 5.73% | 8.06% |
| C13.1 | 4.56 | *N*-Dealkylation + hydroxylation (Cumyl) | C_17_H_17_N_3_O_2_ | 296.1394 (-3.05) | 145.0396; 135.0804 | 0.31% | 0.06% | 0.03% |
| C13.2 | 4.82 | *N*-Dealkylation + hydroxylation (IN) | C_17_H_17_N_3_O_2_ | 296.1394 (2.90) | 161.0346; 119.0855 | 0.78% | 0.02% | □ |
| C13.3 | 5.14 | *N*-Dealkylation + hydroxylation (IN) | C_17_H_17_N_3_O_2_ | 296.1394 (-2.32) | 161.0346; 119.0855 | 0.96% | 0.04% | 0.30% |
| C0 | 16.02 | Cumyl-3TMS-PrINACA (Parent) | C_23_H_31_N_3_OSi | 394.2309 (-0.23) | 259.1261; 119.0852; 73.0466 | □ | ■ | ■ |

t_R_: retention time, IN: indazole; S: side chain (3TMS-PrINACA), pHLM: pooled liver microsomes, PHH: primary human hepatocytes, ■: detected but not included in MAR, □: not detected


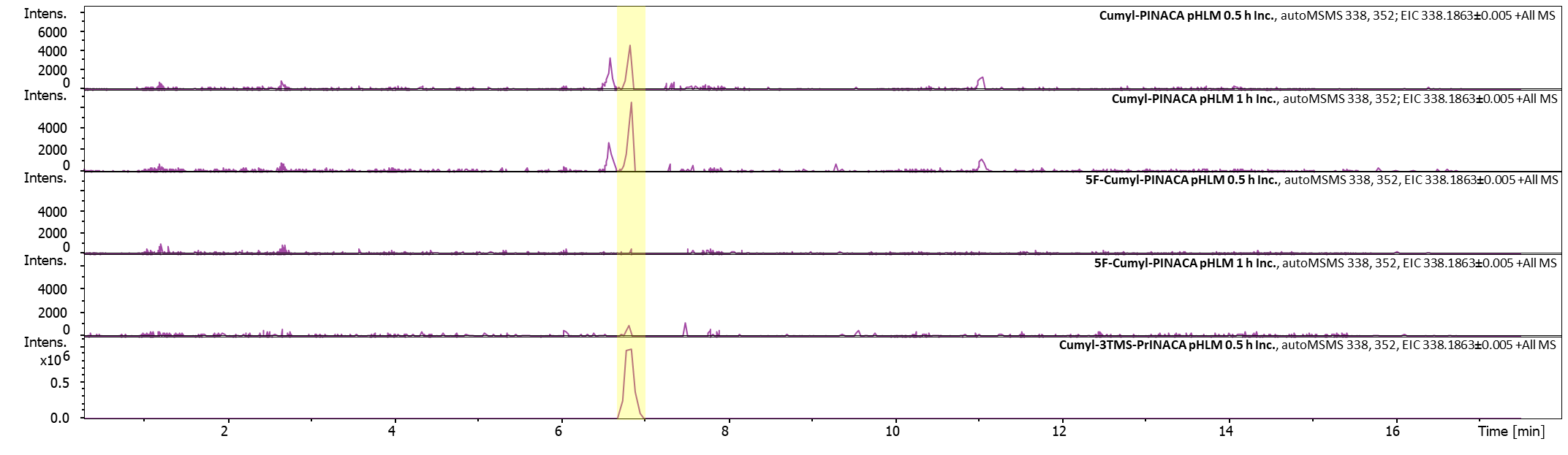


**Fig. S3** Metabolite C6 (Cumyl-3TMS-PrINACA -3TMS + OH, *m/z* = 338.1863) measured in means of LC-HR-MS/MS in positive autoMS/MS mode in pHLM incubations of Cumyl-PINACA (incubation time: 0.5 and 1 h), 5F-Cumyl-PINACA (incubation time: 0.5 and 1 h), and Cumyl-3TMS-PrINACA incubation time: 0.5 h)


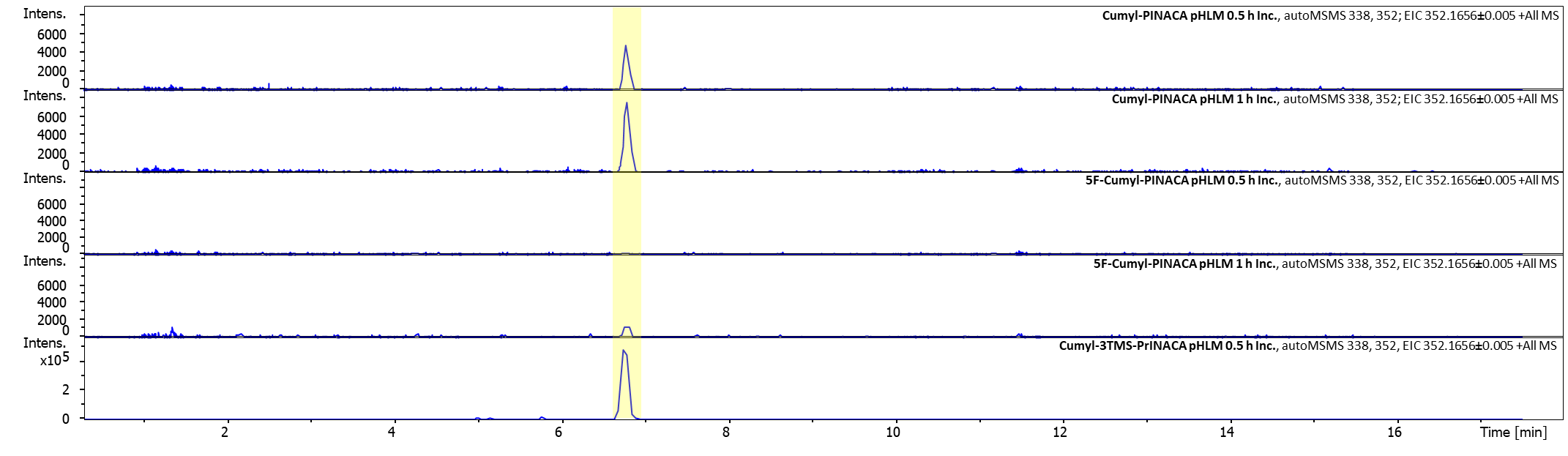


**Fig. S4** Metabolite C8 (Cumyl-3TMS-PrINACA -3TMS + COOH, *m/z* = 352.1666) measured in means of LC-HR-MS/MS in positive autoMS/MS mode in pHLM incubations of Cumyl-PINACA (incubation time: 0.5 and 1 h), 5F-Cumyl-PINACA (incubation time: 0.5 and 1 h), and Cumyl-3TMS-PrINACA incubation time: 0.5 h)

**Table S5** Phase II metabolites of Cumyl-3TMS-PrINACA with retention times, structural modification, exact masses of the protonated molecules, elemental composition, mass errors, and major and characteristic fragment ions. Filled boxes: confirmation, Empty boxes: no confirmation

| **ID** | **t_R_ [min]** | **Modification (Location)** | **Elemental Composition** | **Calculated *m/z* [M+H]^+^ (Δppm)** | **Diagnostic Ions *m/z*** | **Urine** | **PHH** |
| --- | --- | --- | --- | --- | --- | --- | --- |
| C I | 8.22 | Hydroxylation (S) + glucuronidation | C_29_H_39_N_3_O_8_Si | 586.2579 (-0.67) | 275.1210; 292.1476; 119.0855 | ■ | ■ |
| C II | 8.79 | Hydroxylation (S) + glucuronidation | C_29_H_39_N_3_O_8_Si | 586.2579 (0.07) | 275.1210; 292.1476; 119.0855 | ■ | ■ |
| C III | 9.08 | Hydroxylation (S) + glucuronidation | C_29_H_39_N_3_O_8_Si | 586.2579 (1.63) | 275.1210; 292.1476; 119.0855 | ■ | ■ |
| C IV | 4.55 | Cleavage 3TMS + terminal hydroxylation (S) + glucuronidation | C_26_H_31_N_3_O_8_ | 514.2184 (-0.23) | 203.0815; 220.1081; 119.0855 | ■ | ■ |
| **C V** | **4.58** | **Cleavage 3TMS + carboxylic acid formation (S) + glucuronidation** | **C_26_H_29_N_3_O_9_** | **528.1977 (-0.13)** | **217.0608; 234.0873; 119.0855** | ■ | □ |

t_R_: retention time, S: side chain, PHH: primary human hepatocytes

**Table S6** *In silico* metabolite predictions with GLORYx for ADMB-3TMS-PrINACA with the structures, the ranks with the appropriate calculated priority scores, the reaction type, the formula and formula weight (FW)

| **ID** | **Structure (SMILES)** | **Rank** | **Priority Score** | **Reaction Type** | **Formula** | **FW** |
| --- | --- | --- | --- | --- | --- | --- |
| 1 | NC(=O)C(NC(=O)c2nn(CCC[Si](C)(C)C)c1ccccc12)C(C)(C)C | - | - | parent | C_20_H_32_N_4_O_2_Si | 388.5792 |
| 2 | O=C(N)C(NC(=O)c2nn(c1ccccc12)CCC[Si](CO)(C)C)C(C)(C)C | 1 | 0.384 | aliphatic hydroxylation | C_20_H_32_N_4_O_3_Si | 404.5786 |
| 3 | O=C(NO)C(NC(=O)c2nn(c1ccccc12)CCC[Si](C)(C)C)C(C)(C)C | 2 | 0.320 | amine hydroxylation | C_20_H_32_N_4_O_3_Si | 404.5786 |
| 4 | O=C(O)C(NC(=O)c2nn(c1ccccc12)CCC[Si](C)(C)C)C(C)(C)C | 2 | 0.320 | hydrolysis_(primary_amide) | C_20_H_31_N_3_O_3_Si | 389.5639 |
| 5 | O=C(N)C(O)(NC(=O)c2nn(c1ccccc12)CCC[Si](C)(C)C)C(C)(C)C | 2 | 0.320 | aliphatic_hydroxylation_(tert_carbon_next_to_sp^2^) | C_20_H_32_N_4_O_3_Si | 404.5786 |
| 6 | O=C(N)C(NC(=O)c2nn(c1ccccc12)CCC[Si](C)(C)C)C(C)(C)CO | 5 | 0.288 | aliphatic hydroxylation | C_20_H_32_N_4_O_3_Si | 404.5786 |
| 7 | O=C(N)C(NC(=O)c2nn(c1ccccc12)CCC(O)[Si](C)(C)C)C(C)(C)C | 6 | 0.260 | aliphatic hydroxylation | C_20_H32N_4_O_3_Si | 404.5786 |
| 8 | O=C(N)C(N(O)C(=O)c2nn(c1ccccc12)CCC[Si](C)(C)C)C(C)(C)C | 7 | 0.252 | amine hydroxylation | C_20_H_32_N_4_O_3_Si | 404.5786 |
| 9 | O=C(O)c2nn(c1ccccc12)CCC[Si](C)(C)C | 7 | 0.252 | hydrolysis_(secondary_amide) | C_14_H_20_N_2_O_2_Si | 276.4063 |
| 10 | O=C(N)C(N)C(C)(C)C | 7 | 0.252 | hydrolysis_(secondary_amide) | C_6_H_14_N_2_O | 130.1882 |
| 11 | O=C(NC1OC(C(=O)O)C(O)C(O)C1O)C(NC(=O)c3nn(c2ccccc23)CCC  [Si](C)(C)C)C(C)(C)C | 10 | 0.231 | *N*-glucuronidation_(aliphatic_NH_2_) | C_26_H_40_N_4_O_8_Si | 564.7033 |
| 12 | O=CCC[Si](C)(C)C | 11 | 0.136 | *N*-dealkylation | C_6_H_14_OSi | 130.2603 |
| 13 | O=C(N)C(NC(=O)c2nn(c1ccccc12)C(O)CC[Si](C)(C)C)C(C)(C)C | 11 | 0.136 | aliphatic hydroxylation | C_20_H_32_N_4_O_3_Si | 404.5786 |
| 14 | O=C(N)C(NC(=O)c2nnc1ccccc12)C(C)(C)C | 11 | 0.136 | *N*-dealkylation_(nCH_2_) | C_14_H_18_N_4_O_2_ | 274.3183 |
| 15 | OCCC[Si](C)(C)C | 11 | 0.136 | *N*-dealkylation_(nCH_2_) | C_6_H_16_OSi | 132.2761 |
| 16 | O=C(N)C(NC(=O)c2n[n+]([O-])(c1ccccc12)CCC[Si](C)(C)C)C(C)(C)C | 11 | 0.136 | *N*-oxidation | C_20_H_32_N_4_O_3_Si | 404.5786 |
| 17 | O=C(c2nn(c1ccccc12)CCC[Si](C)(C)C)N | 16 | 0.128 | *N*-dealkylation | C_14_H_21_N_3_OSi | 275.4215 |
| 18 | O=C(C(=O)N)C(C)(C)C | 16 | 0.128 | *N*-dealkylation | C_6_H_11_NO_2_ | 129.1570 |
| 19 | O=C(N)C(NC(=O)c2nn(c1c(O)cccc12)CCC[Si](C)(C)C)C(C)(C)C | 18 | 0.124 | aromatic_hydroxylation_(ortho_to_nitrogen) | C_20_H_32_N_4_O_3_Si | 404.5786 |
| 20 | O=C(N)C(NC(=O)c2nn(c1ccc(O)cc12)CCC[Si](C)(C)C)C(C)(C)C | 18 | 0.124 | aromatic_hydroxylation_(para_to_nitrogen) | C_20_H_32_N_4_O_3_Si | 404.5786 |
| 21 | O=C(N)C(NC(=O)c2nn(c1cc(O)ccc12)CCC[Si](C)(C)C)C(C)(C)C | 20 | 0.116 | aromatic_hydroxylation_(para_to_carbon) | C_20_H_32_N_4_O_3_Si | 404.5786 |
| 22 | O=C(N)C(NC(=O)c2[n+](n(c1ccccc12)CCC[Si](C)(C)C)C3OC(C(=O)O)C  (O)C(O)C3O)C(C)(C)C | _21_ | 0.060 | *N*-glucuronidation_(aromatic_=n-) | C_26_H_41_N_4_O_8_Si | 565.7107 |
| 23 | O=C(N)C(NC(=O)c2nn(c1ccccc12)C/C=C/[Si](C)(C)C)C(C)(C)C | 22 | 0.052 | alkyl dehydrogenation | C_20_H_30_N_4_O_2_Si | 386.5633 |
| 24 | O=C(N)C(NC(=O)c1[n+]([O-])n(c2ccccc12)CCC[Si](C)(C)C)C(C)(C)C | 23 | 0.036 | *N*-oxidation | C_20_H_32_N_4_O_3_Si | 404.5786 |
| 25 | O=C(N)C(NC(=O)c2nn(c1ccccc12)CC(O)C[Si](C)(C)C)C(C)(C)C | 23 | 0.036 | aliphatic hydroxylation | C_20_H_32_N_4_O_3_Si | 404.5786 |
| 26 | O=C(N)C(NC(=O)c2nn(c1cccc(O)c12)CCC[Si](C)(C)C)C(C)(C)C | 23 | 0.036 | aromatic hydroxylation | C_20_H_32_N_4_O_3_Si | 404.5786 |
| 27 | O=C(N)C(NC(=O)c2nn(c1ccccc12)/C=C/C[Si](C)(C)C)C(C)(C)C | 26 | 0.027 | alkyl dehydrogenation | C_20_H_30_N_4_O_2_Si | 386.5633 |
| 28 | O=C(N)\C(=[NH+]/C(=O)c2nn(c1ccccc12)CCC[Si](C)(C)C)C(C)(C)C | 27 | 0.0256 | dehydration of *N*-*C* bond | C_20_H_31_N_4_O_2_Si | 387.5707 |
| 29 | O=C(N)\C(=N/C(=O)c2nn(c1ccccc12)CCC[Si](C)(C)C)C(C)(C)C | 27 | 0.0256 | dehydration of N-C bond | C_20_H_30_N_4_O_2_Si | 386.5633 |

**Table S7** *In silico* metabolite predictions with GLORYx for Cumyl-3TMS-PrINACA with the structures, the ranks with the appropriate calculated priority scores, the reaction type, the formula and formula weight (FW)

| **ID** | **Structure (SMILES)** | **Rank** | **Priority**  **Score** | **Reaction Type** | **Formula** | **FW** |
| --- | --- | --- | --- | --- | --- | --- |
| 1 | CC(C)(NC(=O)c2nn(CCC[Si](C)(C)C)c1ccccc12)c3ccccc3 | - | - | parent | C_23_H_31_N_3_OSi | 393.5972 |
| 2 | O=C(c2nn(c1ccccc12)CCC[Si](CO)(C)C)NC(c3ccccc3)(C)C | 1 | 0.416 | aliphatic hydroxylation | C_23_H_31_N_3_O_2_Si | 409.5966 |
| 3 | O=C(c2nn(c1ccccc12)CCC(O)[Si](C)(C)C)NC(c3ccccc3)(C)C | 2 | 0.260 | aliphatic hydroxylation | C_23_H_31_N_3_O_2_Si | 409.5966 |
| 4 | O=C(c2nn(c1ccccc12)CCC[Si](C)(C)C)NC(c3ccc(O)c(OC)c3)(C)C | 3 | 0.224 | aromatic_oxidation | C_24_H_33_N_3_O_3_Si | 439.6226 |
| 5 | O=C(c2nn(c1ccccc12)CCC[Si](C)(C)C)NC(c3ccc(O)cc3)(C)C | 3 | 0.224 | aromatic_hydroxylation_(para_to_carbon) | C_23_H_31_N_3_O_2_Si | 409.5966 |
| 6 | O=C(c2nn(c1ccccc12)CCC[Si](C)(C)C)NC(c3cccc(O)c3)(C)C | 3 | 0.224 | aromatic_hydroxylation_(meta_to_carbon) | C_23_H_31_N_3_O_2_Si | 409.5966 |
| 7 | O=C(O)c2nn(c1ccccc12)CCC[Si](C)(C)C | 6 | 0.204 | hydrolysis_(secondary_amide) | C_14_H_20_N_2_O_2_Si | 276.4063 |
| 8 | O=C(c2nn(c1ccccc12)CCC[Si](C)(C)C)N(O)C(c3ccccc3)(C)C | 6 | 0.204 | amine hydroxylation | C_23_H_31_N_3_O_2_Si | 409.5966 |
| 9 | NC(c1ccccc1)(C)C | 6 | 0.204 | hydrolysis_(secondary_amide) | C_9_H_13_N | 135.2062 |
| 10 | O=C(c2nn(c1c(O)cccc12)CCC[Si](C)(C)C)NC(c3ccccc3)(C)C | 9 | 0.152 | aromatic_hydroxylation_(ortho_to_nitrogen) | C_23_H_31_N_3_O_2_Si | 409.5966 |
| 11 | O=C(c2nn(c1cc(O)ccc12)CCC[Si](C)(C)C)NC(c3ccccc3)(C)C | 9 | 0.152 | aromatic_hydroxylation_(para_to_carbon) | C_23_H_31_N_3_O_2_Si | 409.5966 |
| 12 | O=C(c2nn(c1ccc(O)cc12)CCC[Si](C)(C)C)NC(c3ccccc3)(C)C | 9 | 0.152 | aromatic_hydroxylation_(para_to_nitrogen) | C_23_H_31_N_3_O_2_Si | 409.5966 |
| 13 | O=C(O)C(c1ccccc1)(NC(=O)c3nn(c2ccccc23)CCC[Si](C)(C)C)C | 12 | 0.148 | carboxylation_(primary_carbon_next_to_quart_  carbon) | C_23_H_29_N_3_O_3_Si | 423.5802 |
| 14 | O=C(c2nn(c1ccccc12)CCC[Si](C)(C)C)NC(c3ccccc3)(C)CO | 12 | 0.148 | aliphatic_hydroxylation_(primary_carbon_next_  to_quart_carbon) | C_23_H_31_N_3_O_2_Si | 409.5966 |
| 15 | O=CCC[Si](C)(C)C | 14 | 0.120 | *N*-dealkylation | C_6_H_14_OSi | 130.2603 |
| 16 | [O-][n+]2(nc(c1ccccc12)C(=O)NC(c3ccccc3)(C)C)CCC[Si](C)(C)C | 14 | 0.120 | *N*-oxidation | C_23_H_31_N_3_O_2_Si | 409.5966 |
| 17 | O=C(c2nnc1ccccc12)NC(c3ccccc3)(C)C | 14 | 0.120 | *N*-dealkylation_(nCH_2_) | C_17_H_17_N_3_O | 279.3364 |
| 18 | OCCC[Si](C)(C)C | 14 | 0.120 | *N*-dealkylation_(nCH_2_) | C_6_H_16_OSi | 132.2761 |
| 19 | O=C(c2nn(c1ccccc12)C(O)CC[Si](C)(C)C)NC(c3ccccc3)(C)C | 18 | 0.108 | aliphatic hydroxylation | C_23_H_31_N_3_O_2_Si | 409.5966 |
| 20 | O=C(c2nn(c1ccccc12)C/C=C/[Si](C)(C)C)NC(c3ccccc3)(C)C | 19 | 0.052 | alkyl dehydrogenation | C_23_H_29_N_3_OSi | 391.5814 |
| 21 | O=C(O)C4OC([n+]2n(c1ccccc1c2C(=O)NC(c3ccccc3)(C)C)  CCC[Si](C)(C)C)C(O)C(O)C4O | 20 | 0.044 | *N*-glucuronidation_(aromatic_=n-) | C_29_H_40_N_3_O_7_Si | 570.7288 |
| 22 | O=C(c2nn(c1cccc(O)c12)CCC[Si](C)(C)C)NC(c3ccccc3)(C)C | 20 | 0.044 | aromatic hydroxylation | C_23_H_31_N_3_O_2_Si | 409.5966 |
| 23 | O=C(c2nn(c1ccccc12)CCC[Si](C)(C)C)NC(c3ccccc3O)(C)C | 22 | 0.036 | aromatic hydroxylation | C_23_H_31_N_3_O_2_Si | 409.5966 |
| 24 | [O-][n+]2c(c1ccccc1n2CCC[Si](C)(C)C)C(=O)NC(c3ccccc3)(C)C | 23 | 0.032 | *N*-oxidation | C_23_H_31_N_3_O_2_Si | 409.5966 |
| 25 | O=C(c2nn(c1ccccc12)CC(O)C[Si](C)(C)C)NC(c3ccccc3)(C)C | 23 | 0.032 | aliphatic hydroxylation | C_23_H_31_N_3_O_2_Si | 409.5966 |
| 26 | O=C(c2nn(c1ccccc12)/C=C/C[Si](C)(C)C)NC(c3ccccc3)(C)C | 25 | 0.022 | alkyl dehydrogenation | C_23_H_29_N_3_OSi | 391.5814 |

**Table S8** *In silico* metabolite predictions with BioTransformer 3.0 for the analogue of ADMB-3TMS-PrINACA with Si-C exchange (“**ADMB-4,4-dimethyl-PINACA”**) with structures, the biosystem, the reaction, the formula and formula weight (FW)

| **ID** | **Structure (SMILES)** | **Biosystem** | | **Reaction** | **Formula** | **FW** |
| --- | --- | --- | --- | --- | --- | --- |
| 1 | N(C(=O)C(NC(=O)C1=NN(CCCC(C)(C)C)C2=CC=CC=C21)C(C)(C)C)C3C(C(O)C(C(O3)C(O)=O)O)O | | Human | N-Glucuronidation of amide | C_27_H_40_N_4_O_8_ | 548.2846 |
| 2 | NC(=O)C(NC(=O)C1=NN(CCCC(C)(C)C)C2=CC(=CC=C21)O)C(C)(C)C | | Human | Aromatic hydroxylation of fused benzene ring AndFromCyProduct | C_21_H_32_N_4_O_3_ | 388.2474 |
| 3 | NC(=O)C(NC(=O)C1=NN(CCCC(C)(C)C)C2=CC=C(C=C21)O)C(C)(C)C | | Human | Aromatic hydroxylation of fused benzene ring AndFromCyProduct | C_21_H_32_N_4_O_3_ | 388.2474 |
| 4 | NC(=O)C(NC(=O)C1=NN(CCCC(C)(C)C)C2=C(C=CC=C21)O)C(C)(C)C | | Human | Aromatic hydroxylation of fused benzene ring | C_21_H_32_N_4_O_3_ | 388.2474 |
| 5 | NC(=O)C(NC(=O)C1=NN(CCCC(C)(C)C)C2=CC=CC(=C21)O)C(C)(C)C | | Human | Aromatic hydroxylation of fused benzene ring | C_21_H_32_N_4_O_3_ | 388.2474 |
| 6 | NC(=O)C(NC(=O)C1=NN(CCCC(C)(C)C)C2=CC=CC=C21)C(C)(C)CO | | Human | Hydroxylation of terminal methyl AndFromCyProduct | C_21_H_32_N_4_O_3_ | 388.2474 |
| 7 | NC(=O)C(NC(=O)C1=NN(CCCC(C)(C)CO)C2=CC=CC=C21)C(C)(C)C | | Human | Hydroxylation of terminal methyl AndFromCyProduct | C_21_H_32_N_4_O_3_ | 388.2474 |
| 8 | NC(=O)C(NC(=O)C1=NN(CCCC(C)(C)C)C2=CC(=CC=C21)OC3OC(C(O)C(O)C3O)C(O)=O)C(C)(C)C | | Human | Aromatic OH-glucuronidation | C_27_H_40_N_4_O_9_ | 564.2795 |
| 9 | N(C(=O)C(NC(=O)C1=NN(CCCC(C)(C)C)C2=CC(=CC=C21)O)C(C)(C)C)C3C(C(O)C(C(O3)C(O)=O)O)O | | Human | N-Glucuronidation of amide | C_27_H_40_N_4_O_9_ | 564.2795 |
| 10 | NC(=O)C(NC(=O)C1=NN(CCCC(C)(C)C)C2=CC(=C(C=C21)O)O)C(C)(C)C | | Human | Aromatic hydroxylation of fused benzene ring AndFromCyProduct | C_21_H_32_N_4_O_4_ | 404.2423 |
| 11 | NC(=O)C(NC(=O)C1=NN(CCCC(C)(C)C)C2=CC(=CC(=C21)O)O)C(C)(C)C | | Human | Aromatic hydroxylation of fused benzene ring | C_21_H_32_N_4_O_4_ | 404.2423 |
| 12 | NC(=O)C(NC(=O)C1=NN(CCCC(C)(C)C)C2=CC(=CC=C21)O)C(C)(C)CO | | Human | Hydroxylation of terminal methyl AndFromCyProduct | C_21_H_32_N_4_O_4_ | 404.2423 |
| 13 | NC(=O)C(NC(=O)C1=NN(CCCC(C)(C)CO)C2=CC(=CC=C21)O)C(C)(C)C | | Human | Hydroxylation of terminal methyl AndFromCyProduct | C_21_H_32_N_4_O_4_ | 404.2423 |
| 14 | NC(=O)C(NC(=O)C1=NN(CCCC(C)(C)C)C2=CC=C(C=C21)OC3OC(C(O)C(O)C3O)C(O)=O)C(C)(C)C | | Human | Aromatic OH-glucuronidation | C_27_H_40_N_4_O_9_ | 564.2795 |
| 15 | N(C(=O)C(NC(=O)C1=NN(CCCC(C)(C)C)C2=CC=C(C=C21)O)C(C)(C)C)C3C(C(O)C(C(O3)C(O)=O)O)O | | Human | N-Glucuronidation of amide | C_27_H_40_N_4_O_9_ | 564.2795 |
| 16 | NC(=O)C(NC(=O)C1=NN(CCCC(C)(C)C)C2=C(C=C(C=C21)O)O)C(C)(C)C | | Human | Aromatic hydroxylation of fused benzene ring | C_21_H_32_N_4_O_4_ | 404.2423 |
| 17 | NC(=O)C(NC(=O)C1=NN(CCCC(C)(C)C)C2=CC=C(C(=C21)O)O)C(C)(C)C | | Human | Aromatic hydroxylation of fused benzene ring | C_21_H_32_N_4_O_4_ | 404.2423 |
| 18 | NC(=O)C(NC(=O)C1=NN(CCCC(C)(C)C)C2=CC=C(C=C21)O)C(C)(C)CO | | Human | Hydroxylation of terminal methyl AndFromCyProduct | C_21_H_32_N_4_O_4_ | 404.2423 |
| 19 | NC(=O)C(NC(=O)C1=NN(CCCC(C)(C)CO)C2=CC=C(C=C21)O)C(C)(C)C | | Human | Hydroxylation of terminal methyl AndFromCyProduct | C_21_H_32_N_4_O_4_ | 404.2423 |
| 20 | NC(=O)C(NC(=O)C1=NN(CCCC(C)(C)C)C2=C(C=CC=C21)OC3OC(C(O)C(O)C3O)C(O)=O)C(C)(C)C | | Human | Aromatic OH-glucuronidation | C_27_H_40_N_4_O_9_ | 564.2795 |
| 21 | N(C(=O)C(NC(=O)C1=NN(CCCC(C)(C)C)C2=C(C=CC=C21)O)C(C)(C)C)C3C(C(O)C(C(O3)C(O)=O)O)O | | Human | N-Glucuronidation of amide | C_27_H_40_N_4_O_9_ | 564.2795 |
| 22 | NC(=O)C(NC(=O)C1=NN(CCCC(C)(C)C)C2=C(C(=CC=C21)O)O)C(C)(C)C | | Human | Aromatic hydroxylation of fused benzene ring | C_21_H_32_N_4_O_4_ | 404.2423 |
| 23 | NC(=O)C(NC(=O)C1=NN(CCCC(C)(C)C)C2=C(C=C(C=C21)O)O)C(C)(C)C | | Human | Aromatic hydroxylation of fused benzene ring | C_21_H_32_N_4_O_4_ | 404.2423 |
| 24 | NC(=O)C(NC(=O)C1=NN(CCCC(C)(C)C)C2=C(C=CC=C21)O)C(C)(C)CO | | Human | Hydroxylation of terminal methyl AndFromCyProduct | C_21_H_32_N_4_O_4_ | 404.2423 |
| 25 | NC(=O)C(NC(=O)C1=NN(CCCC(C)(C)CO)C2=C(C=CC=C21)O)C(C)(C)C | | Human | Hydroxylation of terminal methyl AndFromCyProduct | C_21_H_32_N_4_O_4_ | 404.2423 |
| 26 | NC(=O)C(NC(=O)C1=NN(CCCC(C)(C)C)C2=CC=CC(=C21)OC3OC(C(O)C(O)C3O)C(O)=O)C(C)(C)C | | Human | Aromatic OH-glucuronidation | C_27_H_40_N_4_O_9_ | 564.2795 |
| 27 | N(C(=O)C(NC(=O)C1=NN(CCCC(C)(C)C)C2=CC=CC(=C21)O)C(C)(C)C)C3C(C(O)C(C(O3)C(O)=O)O)O | | Human | N-Glucuronidation of amide | C_27_H_40_N_4_O_9_ | 564.2795 |
| 28 | NC(=O)C(NC(=O)C1=NN(CCCC(C)(C)C)C2=CC=CC(=C21)O)C(C)(C)CO | | Human | Hydroxylation of terminal methyl AndFromCyProduct | C_21_H_32_N_4_O_4_ | 404.2423 |
| 29 | NC(=O)C(NC(=O)C1=NN(CCCC(C)(C)CO)C2=CC=CC(=C21)O)C(C)(C)C | | Human | Hydroxylation of terminal methyl AndFromCyProduct | C_21_H_32_N_4_O_4_ | 404.2423 |
| 30 | NC(=O)C(NC(=O)C1=NN(CCCC(C)(C)C)C2=CC=CC=C21)C(C)(C)COC3OC(C(O)=O)C(C(C3O)O)O | | Human | Alkyl-OH-glucuronidation | C_27_H_40_N_4_O_9_ | 564.2795 |
| 31 | N(C(=O)C(NC(=O)C1=NN(CCCC(C)(C)C)C2=CC=CC=C21)C(C)(C)CO)C3C(C(O)C(C(O3)C(O)=O)O)O | | Human | N-Glucuronidation of amide | C_27_H_40_N_4_O_9_ | 564.2795 |
| 32 | NC(=O)C(NC(=O)C1=NN(CCCC(C)(C)C)C2=CC=CC=C21)C(C)(C)COS(O)(=O)=O | | Human | Sulfation of primary alcohol | C_21_H_32_N_4_O_6_S | 468.2042 |
| 33 | NC(=O)C(NC(=O)C1=NN(CCCC(C)(C)C)C2=CC=CC=C21)C(C)(C)C=O | | Human | Oxidation of primary alcohol to aldehyde | C_21_H_30_N_4_O_3_ | 386.2317 |
| 34 | NC(=O)C(NC(=O)C1=NN(CCCC(C)(C)C)C2=CC=CC=C21)C(C)(CO)CO | | Human | Hydroxylation of terminal methyl AndFromCyProduct | C_21_H_32_N_4_O_4_ | 404.2423 |
| 35 | NC(=O)C(NC(=O)C1=NN(CCCC(C)(C)CO)C2=CC=CC=C21)C(C)(C)CO | | Human | Hydroxylation of terminal methyl AndFromCyProduct | C_21_H_32_N_4_O_4_ | 404.2423 |
| 36 | NC(=O)C(NC(=O)C1=NN(CCCC(C)(C)COC2OC(C(O)=O)C(C(C2O)O)O)C3=CC=CC=C31)C(C)(C)C | | Human | Alkyl-OH-glucuronidation | C_27_H_40_N_4_O_9_ | 564.2795 |
| 37 | N(C(=O)C(NC(=O)C1=NN(CCCC(C)(C)CO)C2=CC=CC=C21)C(C)(C)C)C3C(C(O)C(C(O3)C(O)=O)O)O | | Human | N-Glucuronidation of amide | C_27_H_40_N_4_O_9_ | 564.2795 |
| 38 | NC(=O)C(NC(=O)C1=NN(CCCC(C)(C)COS(O)(=O)=O)C2=CC=CC=C21)C(C)(C)C | | Human | Sulfation of primary alcohol | C_21_H_32_N_4_O_6_S | 468.2042 |
| 39 | NC(=O)C(NC(=O)C1=NN(CCCC(C)(C)C=O)C2=CC=CC=C21)C(C)(C)C | | Human | Oxidation of primary alcohol to aldehyde | C_21_H_30_N_4_O_3_ | 386.2317 |
| 40 | NC(=O)C(NC(=O)C1=NN(CCCC(C)(C)CO)C2=CC=CC=C21)C(C)(C)CO | | Human | Hydroxylation of terminal methyl | C_21_H_32_N_4_O_4_ | 404.2423 |
| 41 | NC(=O)C(NC(=O)C1=NN(CCCC(C)(CO)CO)C2=CC=CC=C21)C(C)(C)C | | Human | Hydroxylation of terminal methyl | C_21_H_32_N_4_O_4_ | 404.2423 |
| 42 | NC(=O)C(NC(=O)C1=NN(CCCC(C)(C)C)C2=CC(=C(C=C21)O)OC3OC(C(O)C(O)C3O)C(O)=O)C(C)(C)C | | Human | Aromatic OH-glucuronidation | C_27_H_40_N_4_O_10_ | 580.2744 |
| 43 | NC(=O)C(NC(=O)C1=NN(CCCC(C)(C)C)C2=CC(=C(C=C21)OC3OC(C(O)C(O)C3O)C(O)=O)O)C(C)(C)C | | Human | Aromatic OH-glucuronidation | C_27_H_40_N_4_O_10_ | 580.2744 |
| 44 | N(C(=O)C(NC(=O)C1=NN(CCCC(C)(C)C)C2=CC(=C(C=C21)O)O)C(C)(C)C)C3C(C(O)C(C(O3)C(O)=O)O)O | | Human | N-Glucuronidation of amide | C_27_H_40_N_4_O_10_ | 580.2744 |
| 45 | NC(=O)C(NC(=O)C1=NN(CCCC(C)(C)C)C2=CC(=C(C(=C21)O)O)O)C(C)(C)C | | Human | Aromatic hydroxylation of fused benzene ring | C_21_H_32_N_4_O_5_ | 420.2372 |
| 46 | NC(=O)C(NC(=O)C1=NN(CCCC(C)(C)C)C2=CC(=C(C=C21)O)O)C(C)(C)CO | | Human | Hydroxylation of terminal methyl AndFromCyProduct | C_21_H_32_N_4_O_5_ | 420.2372 |
| 47 | NC(=O)C(NC(=O)C1=NN(CCCC(C)(C)CO)C2=CC(=C(C=C21)O)O)C(C)(C)C | | Human | Hydroxylation of terminal methyl AndFromCyProduct | C_21_H_32_N_4_O_5_ | 420.2372 |
| 48 | NC(=O)C(NC(=O)C1=NN(CCCC(C)(C)C)C2=CC(=CC(=C21)O)OC3OC(C(O)C(O)C3O)C(O)=O)C(C)(C)C | | Human | Aromatic OH-glucuronidation | C_27_H_40_N_4_O_10_ | 580.2744 |
| 49 | NC(=O)C(NC(=O)C1=NN(CCCC(C)(C)C)C2=CC(=CC(=C21)OC3OC(C(O)C(O)C3O)C(O)=O)O)C(C)(C)C | | Human | Aromatic OH-glucuronidation | C_27_H_40_N_4_O_10_ | 580.2744 |
| 50 | N(C(=O)C(NC(=O)C1=NN(CCCC(C)(C)C)C2=CC(=CC(=C21)O)O)C(C)(C)C)C3C(C(O)C(C(O3)C(O)=O)O)O | | Human | N-Glucuronidation of amide | C_27_H_40_N_4_O_10_ | 580.2744 |
| 51 | NC(=O)C(NC(=O)C1=NN(CCCC(C)(C)C)C2=CC(=CC(=C21)O)O)C(C)(C)CO | | Human | Hydroxylation of terminal methyl AndFromCyProduct | C_21_H_32_N_4_O_5_ | 420.2372 |
| 52 | NC(=O)C(NC(=O)C1=NN(CCCC(C)(C)CO)C2=CC(=CC(=C21)O)O)C(C)(C)C | | Human | Hydroxylation of terminal methyl AndFromCyProduct | C_21_H_32_N_4_O_5_ | 420.2372 |
| 53 | NC(=O)C(NC(=O)C1=NN(CCCC(C)(C)C)C2=CC(=CC=C21)OC3OC(C(O)C(O)C3O)C(O)=O)C(C)(C)CO | | Human | Aromatic OH-glucuronidation | C_27_H_40_N_4_O_10_ | 580.2744 |
| 54 | N(C(=O)C(NC(=O)C1=NN(CCCC(C)(C)C)C2=CC(=CC=C21)O)C(C)(C)CO)C3C(C(O)C(C(O3)C(O)=O)O)O | | Human | N-Glucuronidation of amide | C_27_H_40_N_4_O_10_ | 580.2744 |
| 55 | NC(=O)C(NC(=O)C1=NN(CCCC(C)(C)C)C2=CC(=CC=C21)O)C(C)(C)COS(O)(=O)=O | | Human | Sulfation of primary alcohol | C_21_H_32_N_4_O_7_S | 484.1991 |
| 56 | NC(=O)C(NC(=O)C1=NN(CCCC(C)(C)C)C2=CC(=CC=C21)O)C(C)(C)C=O | | Human | Oxidation of primary alcohol to aldehyde | C_21_H_30_N_4_O_4_ | 402.2267 |
| 57 | NC(=O)C(NC(=O)C1=NN(CCCC(C)(C)C)C2=CC(=CC=C21)O)C(C)(CO)CO | | Human | Hydroxylation of terminal methyl AndFromCyProduct | C_21_H_32_N_4_O_5_ | 420.2372 |
| 58 | NC(=O)C(NC(=O)C1=NN(CCCC(C)(C)CO)C2=CC(=CC=C21)O)C(C)(C)CO | | Human | Hydroxylation of terminal methyl AndFromCyProduct | C_21_H_32_N_4_O_5_ | 420.2372 |
| 59 | NC(=O)C(NC(=O)C1=NN(CCCC(C)(C)CO)C2=CC(=CC=C21)OC3OC(C(O)C(O)C3O)C(O)=O)C(C)(C)C | | Human | Aromatic OH-glucuronidation | C_27_H_40_N_4_O_10_ | 580.2744 |
| 60 | N(C(=O)C(NC(=O)C1=NN(CCCC(C)(C)CO)C2=CC(=CC=C21)O)C(C)(C)C)C3C(C(O)C(C(O3)C(O)=O)O)O | | Human | N-Glucuronidation of amide | C_27_H_40_N_4_O_10_ | 580.2744 |
| 61 | NC(=O)C(NC(=O)C1=NN(CCCC(C)(C)COS(O)(=O)=O)C2=CC(=CC=C21)O)C(C)(C)C | | Human | Sulfation of primary alcohol | C_21_H_32_N_4_O_7_S | 484.1991 |
| 62 | NC(=O)C(NC(=O)C1=NN(CCCC(C)(C)C=O)C2=CC(=CC=C21)O)C(C)(C)C | | Human | Oxidation of primary alcohol to aldehyde | C_21_H_30_N_4_O_4_ | 402.2267 |
| 63 | NC(=O)C(NC(=O)C1=NN(CCCC(C)(C)CO)C2=CC(=CC=C21)O)C(C)(C)CO | | Human | Hydroxylation of terminal methyl AndFromCyProduct | C_21_H_32_N_4_O_5_ | 420.2372 |
| 64 | NC(=O)C(NC(=O)C1=NN(CCCC(C)(CO)CO)C2=CC(=CC=C21)O)C(C)(C)C | | Human | Hydroxylation of terminal methyl AndFromCyProduct | C_21_H_32_N_4_O_5_ | 420.2372 |
| 65 | NC(=O)C(NC(=O)C1=NN(CCCC(C)(C)C)C2=C(C=C(C=C21)O)OC3OC(C(O)C(O)C3O)C(O)=O)C(C)(C)C | | Human | Aromatic OH-glucuronidation | C_27_H_40_N_4_O_10_ | 580.2744 |
| 66 | NC(=O)C(NC(=O)C1=NN(CCCC(C)(C)C)C2=C(C=C(C=C21)OC3OC(C(O)C(O)C3O)C(O)=O)O)C(C)(C)C | | Human | Aromatic OH-glucuronidation | C_27_H_40_N_4_O_10_ | 580.2744 |
| 67 | N(C(=O)C(NC(=O)C1=NN(CCCC(C)(C)C)C2=C(C=C(C=C21)O)O)C(C)(C)C)C3C(C(O)C(C(O3)C(O)=O)O)O | | Human | N-Glucuronidation of amide | C_27_H_40_N_4_O_10_ | 580.2744 |
| 68 | NC(=O)C(NC(=O)C1=NN(CCCC(C)(C)C)C2=C(C=C(C=C21)O)O)C(C)(C)CO | | Human | Hydroxylation of terminal methyl AndFromCyProduct | C_21_H_32_N_4_O_5_ | 420.2372 |
| 69 | NC(=O)C(NC(=O)C1=NN(CCCC(C)(C)CO)C2=C(C=C(C=C21)O)O)C(C)(C)C | | Human | Hydroxylation of terminal methyl AndFromCyProduct | C_21_H_32_N_4_O_5_ | 420.2372 |
| 70 | NC(=O)C(NC(=O)C1=NN(CCCC(C)(C)C)C2=CC=C(C(=C21)O)OC3OC(C(O)C(O)C3O)C(O)=O)C(C)(C)C | | Human | Aromatic OH-glucuronidation | C_27_H_40_N_4_O_10_ | 580.2744 |
| 71 | NC(=O)C(NC(=O)C1=NN(CCCC(C)(C)C)C2=CC=C(C(=C21)OC3OC(C(O)C(O)C3O)C(O)=O)O)C(C)(C)C | | Human | Aromatic OH-glucuronidation | C_27_H_40_N_4_O_10_ | 580.2744 |
| 72 | N(C(=O)C(NC(=O)C1=NN(CCCC(C)(C)C)C2=CC=C(C(=C21)O)O)C(C)(C)C)C3C(C(O)C(C(O3)C(O)=O)O)O | | Human | N-Glucuronidation of amide | C_27_H_40_N_4_O_10_ | 580.2744 |
| 73 | NC(=O)C(NC(=O)C1=NN(CCCC(C)(C)C)C2=CC=C(C(=C21)O)O)C(C)(C)CO | | Human | Hydroxylation of terminal methyl AndFromCyProduct | C_21_H_32_N_4_O_5_ | 420.2372 |
| 74 | NC(=O)C(NC(=O)C1=NN(CCCC(C)(C)CO)C2=CC=C(C(=C21)O)O)C(C)(C)C | | Human | Hydroxylation of terminal methyl AndFromCyProduct | C_21_H_32_N_4_O_5_ | 420.2372 |
| 75 | NC(=O)C(NC(=O)C1=NN(CCCC(C)(C)C)C2=CC=C(C=C21)OC3OC(C(O)C(O)C3O)C(O)=O)C(C)(C)CO | | Human | Aromatic OH-glucuronidation | C_27_H_40_N_4_O_10_ | 580.2744 |
| 76 | N(C(=O)C(NC(=O)C1=NN(CCCC(C)(C)C)C2=CC=C(C=C21)O)C(C)(C)CO)C3C(C(O)C(C(O3)C(O)=O)O)O | | Human | N-Glucuronidation of amide | C_27_H_40_N_4_O_10_ | 580.2744 |
| 77 | NC(=O)C(NC(=O)C1=NN(CCCC(C)(C)C)C2=CC=C(C=C21)O)C(C)(C)COS(O)(=O)=O | | Human | Sulfation of primary alcohol | C_21_H_32_N_4_O_7_S | 484.1991 |
| 78 | NC(=O)C(NC(=O)C1=NN(CCCC(C)(C)C)C2=CC=C(C=C21)O)C(C)(C)C=O | | Human | Oxidation of primary alcohol to aldehyde | C_21_H_30_N_4_O_4_ | 402.2267 |
| 79 | NC(=O)C(NC(=O)C1=NN(CCCC(C)(C)C)C2=CC=C(C=C21)O)C(C)(CO)CO | | Human | Hydroxylation of terminal methyl AndFromCyProduct | C_21_H_32_N_4_O_5_ | 420.2372 |
| 80 | NC(=O)C(NC(=O)C1=NN(CCCC(C)(C)CO)C2=CC=C(C=C21)O)C(C)(C)CO | | Human | Hydroxylation of terminal methyl AndFromCyProduct | C_21_H_32_N_4_O_5_ | 420.2372 |
| 81 | NC(=O)C(NC(=O)C1=NN(CCCC(C)(C)CO)C2=CC=C(C=C21)OC3OC(C(O)C(O)C3O)C(O)=O)C(C)(C)C | | Human | Aromatic OH-glucuronidation | C_27_H_40_N_4_O_10_ | 580.2744 |
| 82 | N(C(=O)C(NC(=O)C1=NN(CCCC(C)(C)CO)C2=CC=C(C=C21)O)C(C)(C)C)C3C(C(O)C(C(O3)C(O)=O)O)O | | Human | N-Glucuronidation of amide | C_27_H_40_N_4_O_10_ | 580.2744 |
| 83 | NC(=O)C(NC(=O)C1=NN(CCCC(C)(C)COS(O)(=O)=O)C2=CC=C(C=C21)O)C(C)(C)C | | Human | Sulfation of primary alcohol | C_21_H_32_N_4_O_7_S | 484.1991 |
| 84 | NC(=O)C(NC(=O)C1=NN(CCCC(C)(C)C=O)C2=CC=C(C=C21)O)C(C)(C)C | | Human | Oxidation of primary alcohol to aldehyde | C_21_H_30_N_4_O_4_ | 402.2267 |
| 85 | NC(=O)C(NC(=O)C1=NN(CCCC(C)(C)CO)C2=CC=C(C=C21)O)C(C)(C)CO | | Human | Hydroxylation of terminal methyl AndFromCyProduct | C_21_H_32_N_4_O_5_ | 420.2372 |
| 86 | NC(=O)C(NC(=O)C1=NN(CCCC(C)(CO)CO)C2=CC=C(C=C21)O)C(C)(C)C | | Human | Hydroxylation of terminal methyl AndFromCyProduct | C_21_H_32_N_4_O_5_ | 420.2372 |
| 87 | NC(=O)C(NC(=O)C1=NN(CCCC(C)(C)C)C2=C(C(=CC=C21)O)OC3OC(C(O)C(O)C3O)C(O)=O)C(C)(C)C | | Human | Aromatic OH-glucuronidation | C_27_H_40_N_4_O_10_ | 580.2744 |
| 88 | NC(=O)C(NC(=O)C1=NN(CCCC(C)(C)C)C2=C(C(=CC=C21)OC3OC(C(O)C(O)C3O)C(O)=O)O)C(C)(C)C | | Human | Aromatic OH-glucuronidation | C_27_H_40_N_4_O_10_ | 580.2744 |
| 89 | N(C(=O)C(NC(=O)C1=NN(CCCC(C)(C)C)C2=C(C(=CC=C21)O)O)C(C)(C)C)C3C(C(O)C(C(O3)C(O)=O)O)O | | Human | N-Glucuronidation of amide | C_27_H_40_N_4_O_10_ | 580.2744 |
| 90 | NC(=O)C(NC(=O)C1=NN(CCCC(C)(C)C)C2=C(C(=C(C=C21)O)O)O)C(C)(C)C | | Human | Aromatic hydroxylation of fused benzene ring | C_21_H_32_N_4_O_5_ | 420.2372 |
| 91 | NC(=O)C(NC(=O)C1=NN(CCCC(C)(C)C)C2=C(C(=CC=C21)O)O)C(C)(C)CO | | Human | Hydroxylation of terminal methyl AndFromCyProduct | C_21_H_32_N_4_O_5_ | 420.2372 |
| 92 | NC(=O)C(NC(=O)C1=NN(CCCC(C)(C)CO)C2=C(C(=CC=C21)O)O)C(C)(C)C | | Human | Hydroxylation of terminal methyl AndFromCyProduct | C_21_H_32_N_4_O_5_ | 420.2372 |
| 93 | NC(=O)C(NC(=O)C1=NN(CCCC(C)(C)C)C2=C(C=CC=C21)OC3OC(C(O)C(O)C3O)C(O)=O)C(C)(C)CO | | Human | Aromatic OH-glucuronidation | C_27_H_40_N_4_O_10_ | 580.2744 |
| 94 | N(C(=O)C(NC(=O)C1=NN(CCCC(C)(C)C)C2=C(C=CC=C21)O)C(C)(C)CO)C3C(C(O)C(C(O3)C(O)=O)O)O | | Human | N-Glucuronidation of amide | C_27_H_40_N_4_O_10_ | 580.2744 |
| 95 | NC(=O)C(NC(=O)C1=NN(CCCC(C)(C)C)C2=C(C=CC=C21)O)C(C)(C)COS(O)(=O)=O | | Human | Sulfation of primary alcohol | C_21_H_32_N_4_O_7_S | 484.1991 |
| 96 | NC(=O)C(NC(=O)C1=NN(CCCC(C)(C)C)C2=C(C=CC=C21)O)C(C)(C)C=O | | Human | Oxidation of primary alcohol to aldehyde | C_21_H_30_N_4_O_4_ | 402.2267 |
| 97 | NC(=O)C(NC(=O)C1=NN(CCCC(C)(C)C)C2=C(C=CC=C21)O)C(C)(CO)CO | | Human | Hydroxylation of terminal methyl | C_21_H_32_N_4_O_5_ | 420.2372 |
| 98 | NC(=O)C(NC(=O)C1=NN(CCCC(C)(C)CO)C2=C(C=CC=C21)O)C(C)(C)CO | | Human | Hydroxylation of terminal methyl | C_21_H_32_N_4_O_5_ | 420.2372 |
| 99 | NC(=O)C(NC(=O)C1=NN(CCCC(C)(C)CO)C2=C(C=CC=C21)OC3OC(C(O)C(O)C3O)C(O)=O)C(C)(C)C | | Human | Aromatic OH-glucuronidation | C_27_H_4_0N_4_O_10_ | 580.2744 |
| 100 | N(C(=O)C(NC(=O)C1=NN(CCCC(C)(C)CO)C2=C(C=CC=C21)O)C(C)(C)C)C3C(C(O)C(C(O3)C(O)=O)O)O | | Human | N-Glucuronidation of amide | C_27_H_40_N_4_O_10_ | 580.2744 |
| 101 | NC(=O)C(NC(=O)C1=NN(CCCC(C)(C)COS(O)(=O)=O)C2=C(C=CC=C21)O)C(C)(C)C | | Human | Sulfation of primary alcohol | C_21_H_32_N_4_O_7_S | 484.1991 |
| 102 | NC(=O)C(NC(=O)C1=NN(CCCC(C)(C)C=O)C2=C(C=CC=C21)O)C(C)(C)C | | Human | Oxidation of primary alcohol to aldehyde | C_21_H_30_N_4_O_4_ | 402.2267 |
| 103 | NC(=O)C(NC(=O)C1=NN(CCCC(C)(C)CO)C2=C(C=CC=C21)O)C(C)(C)CO | | Human | Hydroxylation of terminal methyl AndFromCyProduct | C_21_H_32_N_4_O_5_ | 420.2372 |
| 104 | NC(=O)C(NC(=O)C1=NN(CCCC(C)(CO)CO)C2=C(C=CC=C21)O)C(C)(C)C | | Human | Hydroxylation of terminal methyl AndFromCyProduct | C_21_H_32_N_4_O_5_ | 420.2372 |
| 105 | NC(=O)C(NC(=O)C1=NN(CCCC(C)(C)C)C2=CC=CC(=C21)OC3OC(C(O)C(O)C3O)C(O)=O)C(C)(C)CO | | Human | Aromatic OH-glucuronidation | C_27_H_40_N_4_O_10_ | 580.2744 |
| 106 | N(C(=O)C(NC(=O)C1=NN(CCCC(C)(C)C)C2=CC=CC(=C21)O)C(C)(C)CO)C3C(C(O)C(C(O3)C(O)=O)O)O | | Human | N-Glucuronidation of amide | C_27_H_40_N_4_O_10_ | 580.2744 |
| 107 | NC(=O)C(NC(=O)C1=NN(CCCC(C)(C)C)C2=CC=CC(=C21)O)C(C)(C)COS(O)(=O)=O | | Human | Sulfation of primary alcohol | C_21_H_32_N_4_O_7_S | 484.1991 |
| 108 | NC(=O)C(NC(=O)C1=NN(CCCC(C)(C)C)C2=CC=CC(=C21)O)C(C)(C)C=O | | Human | Oxidation of primary alcohol to aldehyde | C_21_H_30_N_4_O_4_ | 402.2267 |
| 109 | NC(=O)C(NC(=O)C1=NN(CCCC(C)(C)C)C2=CC=CC(=C21)O)C(C)(CO)CO | | Human | Hydroxylation of terminal methyl AndFromCyProduct | C_21_H_32_N_4_O_5_ | 420.2372 |
| 110 | NC(=O)C(NC(=O)C1=NN(CCCC(C)(C)CO)C2=CC=CC(=C21)O)C(C)(C)CO | | Human | Hydroxylation of terminal methyl AndFromCyProduct | C_21_H_32_N_4_O_5_ | 420.2372 |
| 111 | NC(=O)C(NC(=O)C1=NN(CCCC(C)(C)CO)C2=CC=CC(=C21)OC3OC(C(O)C(O)C3O)C(O)=O)C(C)(C)C | | Human | Aromatic OH-glucuronidation | C_27_H_40_N_4_O_10_ | 580.2744 |
| 112 | N(C(=O)C(NC(=O)C1=NN(CCCC(C)(C)CO)C2=CC=CC(=C21)O)C(C)(C)C)C3C(C(O)C(C(O3)C(O)=O)O)O | | Human | N-Glucuronidation of amide | C_27_H_40_N_4_O_10_ | 580.2744 |
| 113 | NC(=O)C(NC(=O)C1=NN(CCCC(C)(C)COS(O)(=O)=O)C2=CC=CC(=C21)O)C(C)(C)C | | Human | Sulfation of primary alcohol | C_21_H_32_N_4_O_7_S | 484.1991 |
| 114 | NC(=O)C(NC(=O)C1=NN(CCCC(C)(C)C=O)C2=CC=CC(=C21)O)C(C)(C)C | | Human | Oxidation of primary alcohol to aldehyde | C_21_H_30_N_4_O_4_ | 402.2267 |
| 115 | NC(=O)C(NC(=O)C1=NN(CCCC(C)(C)CO)C2=CC=CC(=C21)O)C(C)(C)CO | | Human | Hydroxylation of terminal methyl AndFromCyProduct | C_21_H_32_N_4_O_5_ | 420.2372 |
| 116 | NC(=O)C(NC(=O)C1=NN(CCCC(C)(CO)CO)C2=CC=CC(=C21)O)C(C)(C)C | | Human | Hydroxylation of terminal methyl AndFromCyProduct | C_21_H_32_N_4_O_5_ | 420.2372 |
| 117 | NC(=O)C(NC(=O)C1=NN(CCCC(C)(C)C)C2=CC=CC=C21)C(C)(C)C(=O)O | | Human | Aldehyde oxidation | C_21_H_30_N_4_O_4_ | 402.2267 |
| 118 | N(C(=O)C(NC(=O)C1=NN(CCCC(C)(C)C)C2=CC=CC=C21)C(C)(C)C=O)C3C(C(O)C(C(O3)C(O)=O)O)O | | Human | N-Glucuronidation of amide | C_27_H_38_N_4_O_9_ | 562.2638 |
| 119 | NC(=O)C(NC(=O)C1=NN(CCCC(C)(C)C)C2=CC=CC=C21)C(C)(CO)C=O | | Human | Hydroxylation of terminal methyl AndFromCyProduct | C_21_H_30_N_4_O_4_ | 402.2267 |
| 120 | NC(=O)C(NC(=O)C1=NN(CCCC(C)(C)CO)C2=CC=CC=C21)C(C)(C)C=O | | Human | Hydroxylation of terminal methyl AndFromCyProduct | C_21_H_30_N_4_O_4_ | 402.2267 |
| 121 | NC(=O)C(NC(=O)C1=NN(CCCC(C)(C)C)C2=CC=CC=C21)C(C)(CO)COC3OC(C(O)=O)C(C(C3O)O)O | | Human | Alkyl-OH-glucuronidation | C_27_H_40_N_4_O_10_ | 580.2744 |
| 122 | N(C(=O)C(NC(=O)C1=NN(CCCC(C)(C)C)C2=CC=CC=C21)C(C)(CO)CO)C3C(C(O)C(C(O3)C(O)=O)O)O | | Human | N-Glucuronidation of amide | C_27_H_40_N_4_O_10_ | 580.2744 |
| 123 | NC(=O)C(NC(=O)C1=NN(CCCC(C)(C)C)C2=CC=CC=C21)C(C)(CO)COS(O)(=O)=O | | Human | Sulfation of primary alcohol | C_21_H_32_N_4_O_7_S | 484.1991 |
| 124 | NC(=O)C(NC(=O)C1=NN(CCCC(C)(C)C)C2=CC=CC=C21)C(CO)(CO)CO | | Human | Hydroxylation of terminal methyl AndFromCyProduct | C_21_H_32_N_4_O_5_ | 420.2372 |
| 125 | NC(=O)C(NC(=O)C1=NN(CCCC(C)(C)CO)C2=CC=CC=C21)C(C)(CO)CO | | Human | Hydroxylation of terminal methyl AndFromCyProduct | C_21_H_32_N_4_O_5_ | 420.2372 |
| 126 | NC(=O)C(NC(=O)C1=NN(CCCC(C)(C)CO)C2=CC=CC=C21)C(C)(C)COC3OC(C(O)=O)C(C(C3O)O)O | | Human | Alkyl-OH-glucuronidation | C_27_H_40_N_4_O_10_ | 580.2744 |
| 127 | NC(=O)C(NC(=O)C1=NN(CCCC(C)(C)COC2OC(C(O)=O)C(C(C2O)O)O)C3=CC=CC=C31)C(C)(C)CO | | Human | Alkyl-OH-glucuronidation | C_27_H_40_N_4_O_10_ | 580.2744 |
| 128 | N(C(=O)C(NC(=O)C1=NN(CCCC(C)(C)CO)C2=CC=CC=C21)C(C)(C)CO)C3C(C(O)C(C(O3)C(O)=O)O)O | | Human | N-Glucuronidation of amide | C_27_H_40_N_4_O_10_ | 580.2744 |
| 129 | NC(=O)C(NC(=O)C1=NN(CCCC(C)(C)CO)C2=CC=CC=C21)C(C)(C)COS(O)(=O)=O | | Human | Sulfation of primary alcohol | C_21_H_32_N_4_O_7_S | 484.1991 |
| 130 | NC(=O)C(NC(=O)C1=NN(CCCC(C)(C)COS(O)(=O)=O)C2=CC=CC=C21)C(C)(C)CO | | Human | Sulfation of primary alcohol | C_21_H_32_N_4_O_7_S | 484.1991 |
| 131 | NC(=O)C(NC(=O)C1=NN(CCCC(C)(C)CO)C2=CC=CC=C21)C(C)(C)C=O | | Human | Oxidation of primary alcohol to aldehyde | C_21_H_30_N_4_O_4_ | 402.2267 |
| 132 | NC(=O)C(NC(=O)C1=NN(CCCC(C)(C)C=O)C2=CC=CC=C21)C(C)(C)CO | | Human | Oxidation of primary alcohol to aldehyde | C_21_H_30_N_4_O_4_ | 402.2267 |
| 133 | NC(=O)C(NC(=O)C1=NN(CCCC(C)(C)CO)C2=CC=CC=C21)C(C)(CO)CO | | Human | Hydroxylation of terminal methyl AndFromCyProduct | C_21_H_32_N_4_O_5_ | 420.2372 |
| 134 | NC(=O)C(NC(=O)C1=NN(CCCC(C)(CO)CO)C2=CC=CC=C21)C(C)(C)CO | | Human | Hydroxylation of terminal methyl AndFromCyProduct | C_21_H_32_N_4_O_5_ | 420.2372 |
| 135 | NC(=O)C(NC(=O)C1=NN(CCCC(C)(C)C(=O)O)C2=CC=CC=C21)C(C)(C)C | | Human | Aldehyde oxidation | C_21_H_30_N_4_O_4_ | 402.2267 |
| 136 | N(C(=O)C(NC(=O)C1=NN(CCCC(C)(C)C=O)C2=CC=CC=C21)C(C)(C)C)C3C(C(O)C(C(O3)C(O)=O)O)O | | Human | N-Glucuronidation of amide | C_27_H_38_N_4_O_9_ | 562.2638 |
| 137 | NC(=O)C(NC(=O)C1=NN(CCCC(C)(C)C=O)C2=CC=CC=C21)C(C)(C)CO | | Human | Hydroxylation of terminal methyl AndFromCyProduct | C_21_H_30_N_4_O_4_ | 402.2267 |
| 138 | NC(=O)C(NC(=O)C1=NN(CCCC(C)(CO)C=O)C2=CC=CC=C21)C(C)(C)C | | Human | Hydroxylation of terminal methyl AndFromCyProduct | C_21_H_30_N_4_O_4_ | 402.2267 |
| 139 | NC(=O)C(NC(=O)C1=NN(CCCC(C)(CO)COC2OC(C(O)=O)C(C(C2O)O)O)C3=CC=CC=C31)C(C)(C)C | | Human | Alkyl-OH-glucuronidation | C_27_H_40_N_4_O_10_ | 580.2744 |
| 140 | N(C(=O)C(NC(=O)C1=NN(CCCC(C)(CO)CO)C2=CC=CC=C21)C(C)(C)C)C3C(C(O)C(C(O3)C(O)=O)O)O | | Human | N-Glucuronidation of amide | C_27_H_40_N_4_O_10_ | 580.2744 |
| 141 | NC(=O)C(NC(=O)C1=NN(CCCC(C)(CO)COS(O)(=O)=O)C2=CC=CC=C21)C(C)(C)C | | Human | Sulfation of primary alcohol | C_21_H_32_N_4_O_7_S | 484.1991 |
| 142 | NC(=O)C(NC(=O)C1=NN(CCCC(C)(CO)CO)C2=CC=CC=C21)C(C)(C)CO | | Human | Hydroxylation of terminal methyl AndFromCyProduct | C_21_H_32_N_4_O_5_ | 420.2372 |
| 143 | NC(=O)C(NC(=O)C1=NN(CCCC(CO)(CO)CO)C2=CC=CC=C21)C(C)(C)C | | Human | Hydroxylation of terminal methyl AndFromCyProduct | C_21_H_32_N_4_O_5_ | 420.2372 |

**Table S9** *In silico* metabolite predictions with BioTransformer 3.0 for the analogue of Cumyl-3TMS-PrINACA with Si-C exchange (“**Cumyl-4,4-dimethyl-PINACA”**) with structures, the biosystem, the reaction, the formula and formula weight (FW)

| **ID** | **Structure (SMILES)** | **Biosystem** | **Reaction** | **Formula** | **FW** |
| --- | --- | --- | --- | --- | --- |
| 1 | Oc1ccccc1C(C)(C)NC(=O)c3nn(CCCC(C)(C)C)c2ccccc23 | Human | O-Hydroxylation of monosubstituted benzene | C_24_H_31_N_3_O_2_ | 393.5218 |
| 2 | CC(C)(NC(=O)c2nn(CCCC(C)(C)C)c1cc(O)ccc12)c3ccccc3 | Human | Aromatic hydroxylation of fused benzene ring | C_24_H_31_N_3_O_2_ | 393.5218 |
| 3 | CC(C)(NC(=O)c2nn(CCCC(C)(C)C)c1ccc(O)cc12)c3ccccc3 | Human | Aromatic hydroxylation of fused benzene ring | C_24_H_31_N_3_O_2_ | 393.5218 |
| 4 | Oc1ccc(cc1)C(C)(C)NC(=O)c3nn(CCCC(C)(C)C)c2ccccc23 | Human | Hydroxylation of benzene on carbon para to electron donating group | C_24_H_31_N_3_O_2_ | 393.5218 |
| 5 | CC(C)(NC(=O)c2nn(CCCC(C)(C)C)c1c(O)cccc12)c3ccccc3 | Human | Aromatic hydroxylation of fused benzene ring | C_24_H_31_N_3_O_2_ | 393.5218 |
| 6 | CC(C)(NC(=O)c2nn(CCCC(C)(C)C)c1cccc(O)c12)c3ccccc3 | Human | Aromatic hydroxylation of fused benzene ring | C_24_H_31_N_3_O_2_ | 393.5218 |
| 7 | CC(CO)(NC(=O)c2nn(CCCC(C)(C)C)c1ccccc12)c3ccccc3 | Human | Hydroxylation of terminal methyl | C_24_H_31_N_3_O_2_ | 393.5218 |
| 8 | CC(C)(NC(=O)c2nn(CCCC(C)(C)CO)c1ccccc12)c3ccccc3 | Human | Hydroxylation of terminal methyl | C_24_H_31_N_3_O_2_ | 393.5218 |
| 9 | CC(C)(C)CCCn2nc(c1ccccc12)C(=O)NC(C)(C)c4ccccc4OC3  OC(C(O)C(O)C3O)C(=O)O | Human | Aromatic OH-glucuronidation | C_30_H_39_N_3_O_8_ | 569.6460 |
| 10 | Oc1cc(c(O)cc1)C(C)(C)NC(=O)c3nn(CCCC(C)(C)C)c2ccccc23 | Human | p-Hydroxylation of phenol | C_24_H_31_N_3_O_3_ | 409.5212 |
| 11 | Oc1ccccc1C(C)(C)NC(=O)c3nn(CCCC(C)(C)C)c2cc(O)ccc23 | Human | Aromatic hydroxylation of fused benzene ring | C_24_H_31_N_3_O_3_ | 409.5212 |
| 12 | Oc1ccccc1C(C)(C)NC(=O)c3nn(CCCC(C)(C)C)c2ccc(O)cc23 | Human | Aromatic hydroxylation of fused benzene ring | C_24_H_31_N_3_O_3_ | 409.5212 |
| 13 | Oc1cccc(O)c1C(C)(C)NC(=O)c3nn(CCCC(C)(C)C)c2ccccc23 | Human | Hydroxylation of benzene on carbon ortho to electron donating group | C_24_H_31_N_3_O_3_ | 409.5212 |
| 14 | Oc1ccc(c(O)c1)C(C)(C)NC(=O)c3nn(CCCC(C)(C)C)c2ccccc23 | Human | Hydroxylation of benzene on carbon para to electron donating group | C_24_H_31_N_3_O_3_ | 409.5212 |
| 15 | Oc1ccccc1C(C)(C)NC(=O)c3nn(CCCC(C)(C)C)c2c(O)cccc23 | Human | Aromatic hydroxylation of fused benzene ring | C_24_H_31_N_3_O_3_ | 409.5212 |
| 16 | Oc1ccccc1C(C)(C)NC(=O)c3nn(CCCC(C)(C)C)c2cccc(O)c23 | Human | Aromatic hydroxylation of fused benzene ring | C_24_H_31_N_3_O_3_ | 409.5212 |
| 17 | Oc1ccccc1C(C)(CO)NC(=O)c3nn(CCCC(C)(C)C)c2ccccc23 | Human | Hydroxylation of terminal methyl | C_24_H_31_N_3_O_3_ | 409.5212 |
| 18 | Oc1ccccc1C(C)(C)NC(=O)c3nn(CCCC(C)(C)CO)c2ccccc23 | Human | Hydroxylation of terminal methyl | C_24_H_31_N_3_O_3_ | 409.5212 |
| 19 | Oc1cccc(c1O)C(C)(C)NC(=O)c3nn(CCCC(C)(C)C)c2ccccc23 | Human | O-Hydroxylation of phenol | C_24_H_31_N_3_O_3_ | 409.5212 |
| 20 | CC(C)(NC(=O)c2nn(CCCC(C)(C)C)c3cc(OC1OC(C(O)C(O)C1O)  C(=O)O)ccc23)c4ccccc4 | Human | Aromatic OH-glucuronidation | C_30_H_39_N_3_O_8_ | 569.6460 |
| 21 | CC(C)(NC(=O)c2nn(CCCC(C)(C)C)c1cc(O)c(O)cc12)c3ccccc3 | Human | Aromatic hydroxylation of fused benzene ring | C_24_H_31_N_3_O_3_ | 409.5212 |
| 22 | Oc1ccc(cc1)C(C)(C)NC(=O)c3nn(CCCC(C)(C)C)c2cc(O)ccc23 | Human | Hydroxylation of benzene on carbon para to electron donating group | C_24_H_31_N_3_O_3_ | 409.5212 |
| 23 | CC(C)(NC(=O)c2nn(CCCC(C)(C)C)c1cc(O)cc(O)c12)c3ccccc3 | Human | Aromatic hydroxylation of fused benzene ring | C_24_H_31_N_3_O_3_ | 409.5212 |
| 24 | CC(CO)(NC(=O)c2nn(CCCC(C)(C)C)c1cc(O)ccc12)c3ccccc3 | Human | Hydroxylation of terminal methyl | C_24_H_31_N_3_O_3_ | 409.5212 |
| 25 | CC(C)(NC(=O)c2nn(CCCC(C)(C)CO)c1cc(O)ccc12)c3ccccc3 | Human | Hydroxylation of terminal methyl | C_24_H_31_N_3_O_3_ | 409.5212 |
| 26 | CC(C)(NC(=O)c2nn(CCCC(C)(C)C)c3ccc(OC1OC(C(O)C(O)C1O)  C(=O)O)cc23)c4ccccc4 | Human | Aromatic OH-glucuronidation | C_30_H_39_N_3_O_8_ | 569.6460 |
| 27 | Oc1ccc(cc1)C(C)(C)NC(=O)c3nn(CCCC(C)(C)C)c2ccc(O)cc23 | Human | Hydroxylation of benzene on carbon para to electron donating group | C_24_H_31_N_3_O_3_ | 409.5212 |
| 28 | CC(C)(NC(=O)c2nn(CCCC(C)(C)C)c1c(O)cc(O)cc12)c3ccccc3 | Human | Aromatic hydroxylation of fused benzene ring | C_24_H_31_N_3_O_3_ | 409.5212 |
| 29 | CC(C)(NC(=O)c2nn(CCCC(C)(C)C)c1ccc(O)c(O)c12)c3ccccc3 | Human | Aromatic hydroxylation of fused benzene ring | C_24_H_31_N_3_O_3_ | 409.5212 |
| 30 | CC(CO)(NC(=O)c2nn(CCCC(C)(C)C)c1ccc(O)cc12)c3ccccc3 | Human | Hydroxylation of terminal methyl | C_24_H_31_N_3_O_3_ | 409.5212 |
| 31 | CC(C)(NC(=O)c2nn(CCCC(C)(C)CO)c1ccc(O)cc12)c3ccccc3 | Human | Hydroxylation of terminal methyl | C_24_H_31_N_3_O_3_ | 409.5212 |
| 32 | CC(C)(C)CCCn2nc(c1ccccc12)C(=O)NC(C)(C)c3ccc(cc3)OC4OC  (C(O)C(O)C4O)C(=O)O | Human | Aromatic OH-glucuronidation | C_30_H_39_N_3_O_8_ | 569.6460 |
| 33 | Oc1ccc(cc1O)C(C)(C)NC(=O)c3nn(CCCC(C)(C)C)c2ccccc23 | Human | 2-Hydroxylation of 1,4-disubstituted benzene | C_24_H_31_N_3_O_3_ | 409.5212 |
| 34 | Oc1ccc(cc1)C(C)(C)NC(=O)c3nn(CCCC(C)(C)C)c2c(O)cccc23 | Human | Aromatic hydroxylation of fused benzene ring | C_24_H_31_N_3_O_3_ | 409.5212 |
| 35 | Oc1ccc(cc1)C(C)(C)NC(=O)c3nn(CCCC(C)(C)C)c2cccc(O)c23 | Human | Aromatic hydroxylation of fused benzene ring | C_24_H_31_N_3_O_3_ | 409.5212 |
| 36 | Oc1ccc(cc1)C(C)(CO)NC(=O)c3nn(CCCC(C)(C)C)c2ccccc23 | Human | Hydroxylation of terminal methyl | C_24_H_31_N_3_O_3_ | 409.5212 |
| 37 | Oc1ccc(cc1)C(C)(C)NC(=O)c3nn(CCCC(C)(C)CO)c2ccccc23 | Human | Hydroxylation of terminal methyl | C_24_H_31_N_3_O_3_ | 409.5212 |
| 38 | CC(C)(NC(=O)c2nn(CCCC(C)(C)C)c1ccccc12)c3ccccc3 | Gut microbial environment | 4'-Dehydroxylation of substituted benzene | C_24_H_31_N_3_O | 377.5224 |
| 39 | CC(C)(NC(=O)c3nn(CCCC(C)(C)C)c2c(OC1OC(C(O)C(O)C1O)C  (=O)O)cccc23)c4ccccc4 | Human | Aromatic OH-glucuronidation | C_30_H_39_N_3_O_8_ | 569.6460 |
| 40 | CC(C)(NC(=O)c2nn(CCCC(C)(C)C)c1c(O)c(O)ccc12)c3ccccc3 | Human | Aromatic hydroxylation of fused benzene ring | C_24_H_31_N_3_O_3_ | 409.5212 |
| 41 | CC(C)(NC(=O)c2nn(CCCC(C)(C)C)c1c(O)cc(O)cc12)c3ccccc3 | Human | Aromatic hydroxylation of fused benzene ring | C_24_H_31_N_3_O_3_ | 409.5212 |
| 42 | CC(CO)(NC(=O)c2nn(CCCC(C)(C)C)c1c(O)cccc12)c3ccccc3 | Human | Hydroxylation of terminal methyl | C_24_H_31_N_3_O_3_ | 409.5212 |
| 43 | CC(C)(NC(=O)c2nn(CCCC(C)(C)CO)c1c(O)cccc12)c3ccccc3 | Human | Hydroxylation of terminal methyl | C_24_H_31_N_3_O_3_ | 409.5212 |
| 44 | CC(C)(NC(=O)c3nn(CCCC(C)(C)C)c2cccc(OC1OC(C(O)C(O)C1O)  C(=O)O)c23)c4ccccc4 | Human | Aromatic OH-glucuronidation | C_30_H_39_N_3_O_8_ | 569.6460 |
| 45 | CC(CO)(NC(=O)c2nn(CCCC(C)(C)C)c1cccc(O)c12)c3ccccc3 | Human | Hydroxylation of terminal methyl | C_24_H_31_N_3_O_3_ | 409.5212 |
| 46 | CC(C)(NC(=O)c2nn(CCCC(C)(C)CO)c1cccc(O)c12)c3ccccc3 | Human | Hydroxylation of terminal methyl | C_24_H_31_N_3_O_3_ | 409.5212 |
| 47 | CC(C)(C)CCCn2nc(c1ccccc12)C(=O)NC(C)(COC3OC(C(O)C(O)  C3O)C(=O)O)c4ccccc4 | Human | Alkyl-OH-glucuronidation | C_30_H_39_N_3_O_8_ | 569.6460 |
| 48 | O=S(=O)(O)OCC(C)(NC(=O)c2nn(CCCC(C)(C)C)c1ccccc12)c3  ccccc3 | Human | Sulfation of primary alcohol | C_24_H_31_N_3_O_5_S | 473.5850 |
| 49 | O=CC(C)(NC(=O)c2nn(CCCC(C)(C)C)c1ccccc12)c3ccccc3 | Human | Oxidation of primary alcohol to aldehyde | C_24_H_29_N_3_O_2_ | 391.5060 |
| 50 | CC(CO)(NC(=O)c2nn(CCCC(C)(C)CO)c1ccccc12)c3ccccc3 | Human | Hydroxylation of terminal methyl | C_24_H_31_N_3_O_3_ | 409.5212 |
| 51 | OCC(CO)(NC(=O)c2nn(CCCC(C)(C)C)c1ccccc12)c3ccccc3 | Human | Hydroxylation of terminal methyl | C_24_H_31_N_3_O_3_ | 409.5212 |
| 52 | CC(C)(NC(=O)c3nn(CCCC(C)(C)COC1OC(C(O)C(O)C1O)C(=O)  O)c2ccccc23)c4ccccc4 | Human | Alkyl-OH-glucuronidation | C_30_H_39_N_3_O_8_ | 569.6460 |
| 53 | CC(C)(NC(=O)c2nn(CCCC(C)(C)COS(=O)(=O)O)c1ccccc12)c3  ccccc3 | Human | Sulfation of primary alcohol | C_24_H_31_N_3_O_5_S | 473.5850 |
| 54 | CC(C)(NC(=O)c2nn(CCCC(C)(C)C=O)c1ccccc12)c3ccccc3 | Human | Oxidation of primary alcohol to aldehyde | C_24_H_29_N_3_O_2_ | 391.5060 |
| 55 | CC(CO)(NC(=O)c2nn(CCCC(C)(C)CO)c1ccccc12)c3ccccc3 | Human | Hydroxylation of terminal methyl | C_24_H_31_N_3_O_3_ | 4095.212 |
| 56 | CC(C)(NC(=O)c2nn(CCCC(C)(CO)CO)c1ccccc12)c3ccccc3 | Human | Hydroxylation of terminal methyl | C_24_H_31_N_3_O_3_ | 4095.212 |
| 57 | CC(C)(C)CCCn2nc(c1ccccc12)C(=O)NC(C)(C)c4cc(O)ccc4OC3  OC(C(O)C(O)C3O)C(=O)O | Human | Aromatic OH-glucuronidation | C_30_H_39_N_3_O_9_ | 585.6454 |
| 58 | CC(C)(C)CCCn2nc(c1ccccc12)C(=O)NC(C)(C)c3cc(ccc3O)OC4  OC(C(O)C(O)C4O)C(=O)O | Human | Aromatic OH-glucuronidation | C_30_H_39_N_3_O_9_ | 585.6454 |
| 59 | Oc1cc(c(O)cc1)C(C)(C)NC(=O)c3nn(CCCC(C)(C)C)c2cc(O)ccc23 | Human | Aromatic hydroxylation of fused benzene ring | C_24_H_31_N_3_O_4_ | 425.5206 |
| 60 | Oc1cc(c(O)cc1)C(C)(C)NC(=O)c3nn(CCCC(C)(C)C)c2ccc(O)cc23 | Human | Aromatic hydroxylation of fused benzene ring | C_24_H_31_N_3_O_4_ | 425.5206 |
| 61 | Oc1cc(c(O)cc1)C(C)(C)NC(=O)c3nn(CCCC(C)(C)C)c2c(O)cccc23 | Human | Aromatic hydroxylation of fused benzene ring | C_24_H_31_N_3_O_4_ | 425.5206 |
| 62 | Oc1cc(c(O)cc1)C(C)(C)NC(=O)c3nn(CCCC(C)(C)C)c2cccc(O)c23 | Human | Aromatic hydroxylation of fused benzene ring | C_24_H_31_N_3_O_4_ | 425.5206 |
| 63 | Oc1cc(c(O)cc1)C(C)(CO)NC(=O)c3nn(CCCC(C)(C)C)c2ccccc23 | Human | Hydroxylation of terminal methyl | C_24_H_31_N_3_O_4_ | 425.5206 |
| 64 | Oc1cc(c(O)cc1)C(C)(C)NC(=O)c3nn(CCCC(C)(C)CO)c2ccccc23 | Human | Hydroxylation of terminal methyl | C_24_H_31_N_3_O_4_ | 425.5206 |
| 65 | CC(C)(C)CCCn2nc(c1ccc(O)cc12)C(=O)NC(C)(C)c4ccccc4OC3OC  (C(O)C(O)C3O)C(=O)O | Human | Aromatic OH-glucuronidation | C_30_H_39_N_3_O_9_ | 585.6454 |
| 66 | Oc1ccccc1C(C)(C)NC(=O)c3nn(CCCC(C)(C)C)c4cc(OC2OC(C(O)C  (O)C2O)C(=O)O)ccc34 | Human | Aromatic OH-glucuronidation | C_30_H_39_N_3_O_9_ | 585.6454 |
| 67 | Oc1ccccc1C(C)(C)NC(=O)c3nn(CCCC(C)(C)C)c2cc(O)c(O)cc23 | Human | Aromatic hydroxylation of fused benzene ring | C_24_H_31_N_3_O_4_ | 425.5206 |
| 68 | Oc1cccc(O)c1C(C)(C)NC(=O)c3nn(CCCC(C)(C)C)c2cc(O)ccc23 | Human | Hydroxylation of benzene on carbon ortho to electron donating group | C_24_H_31_N_3_O_4_ | 425.5206 |
| 69 | Oc1ccc(c(O)c1)C(C)(C)NC(=O)c3nn(CCCC(C)(C)C)c2cc(O)ccc23 | Human | Hydroxylation of benzene on carbon para to electron donating group | C_24_H_31_N_3_O_4_ | 425.5206 |
| 70 | Oc1ccccc1C(C)(C)NC(=O)c3nn(CCCC(C)(C)C)c2cc(O)cc(O)c23 | Human | Aromatic hydroxylation of fused benzene ring | C_24_H_31_N_3_O_4_ | 425.5206 |
| 71 | Oc1ccccc1C(C)(CO)NC(=O)c3nn(CCCC(C)(C)C)c2cc(O)ccc23 | Human | Hydroxylation of terminal methyl | C_24_H_31_N_3_O_4_ | 425.5206 |
| 72 | Oc1ccccc1C(C)(C)NC(=O)c3nn(CCCC(C)(C)CO)c2cc(O)ccc23 | Human | Hydroxylation of terminal methyl | C_24_H_31_N_3_O_4_ | 425.5206 |
| 73 | Oc1cccc(c1O)C(C)(C)NC(=O)c3nn(CCCC(C)(C)C)c2cc(O)ccc23 | Human | O-Hydroxylation of phenol | C_24_H_31_N_3_O_4_ | 425.5206 |
| 74 | CC(C)(C)CCCn2nc(c1cc(O)ccc12)C(=O)NC(C)(C)c4ccccc4OC3OC  (C(O)C(O)C3O)C(=O)O | Human | Aromatic OH-glucuronidation | C_30_H_39_N_3_O_9_ | 585.6454 |
| 75 | Oc1ccccc1C(C)(C)NC(=O)c3nn(CCCC(C)(C)C)c4ccc(OC2OC(C(O)C  (O)C2O)C(=O)O)cc34 | Human | Aromatic OH-glucuronidation | C_30_H_39_N_3_O_9_ | 585.6454 |
| 76 | Oc1cccc(O)c1C(C)(C)NC(=O)c3nn(CCCC(C)(C)C)c2ccc(O)cc23 | Human | Hydroxylation of benzene on carbon ortho to electron donating group | C_24_H_31_N_3_O_4_ | 425.5206 |
| 77 | Oc1ccc(c(O)c1)C(C)(C)NC(=O)c3nn(CCCC(C)(C)C)c2ccc(O)cc23 | Human | Hydroxylation of benzene on carbon para to electron donating group | C_24_H_31_N_3_O_4_ | 425.5206 |
| 78 | Oc1ccccc1C(C)(C)NC(=O)c3nn(CCCC(C)(C)C)c2c(O)cc(O)cc23 | Human | Aromatic hydroxylation of fused benzene ring | C_24_H_31_N_3_O_4_ | 425.5206 |
| 79 | Oc1ccccc1C(C)(C)NC(=O)c3nn(CCCC(C)(C)C)c2ccc(O)c(O)c23 | Human | Aromatic hydroxylation of fused benzene ring | C_24_H_31_N_3_O_4_ | 425.5206 |
| 80 | Oc1ccccc1C(C)(CO)NC(=O)c3nn(CCCC(C)(C)C)c2ccc(O)cc23 | Human | Hydroxylation of terminal methyl | C_24_H_31_N_3_O_4_ | 425.5206 |
| 81 | Oc1ccccc1C(C)(C)NC(=O)c3nn(CCCC(C)(C)CO)c2ccc(O)cc23 | Human | Hydroxylation of terminal methyl | C_24_H_31_N_3_O_4_ | 425.5206 |
| 82 | Oc1cccc(c1O)C(C)(C)NC(=O)c3nn(CCCC(C)(C)C)c2ccc(O)cc23 | Human | O-Hydroxylation of phenol | C_24_H_31_N_3_O_4_ | 425.5206 |
| 83 | CC(C)(C)CCCn2nc(c1ccccc12)C(=O)NC(C)(C)c4c(OC3OC(C(O)C(O)  C3O)C(=O)O)cccc4O | Human | Aromatic OH-glucuronidation | C_30_H_39_N_3_O_9_ | 585.6454 |
| 84 | Oc1cc(O)cc(O)c1C(C)(C)NC(=O)c3nn(CCCC(C)(C)C)c2ccccc23 | Human | Hydroxylation of benzene on carbon para to electron donating group | C_24_H_31_N_3_O_4_ | 425.5206 |
| 85 | Oc1cccc(O)c1C(C)(C)NC(=O)c3nn(CCCC(C)(C)C)c2c(O)cccc23 | Human | Aromatic hydroxylation of fused benzene ring | C_24_H_31_N_3_O_4_ | 425.5206 |
| 86 | Oc1cccc(O)c1C(C)(C)NC(=O)c3nn(CCCC(C)(C)C)c2cccc(O)c23 | Human | Aromatic hydroxylation of fused benzene ring | C_24_H_31_N_3_O_4_ | 425.5206 |
| 87 | Oc1cccc(O)c1C(C)(CO)NC(=O)c3nn(CCCC(C)(C)C)c2ccccc23 | Human | Hydroxylation of terminal methyl | C_24_H_31_N_3_O_4_ | 425.5206 |
| 88 | Oc1cccc(O)c1C(C)(C)NC(=O)c3nn(CCCC(C)(C)CO)c2ccccc23 | Human | Hydroxylation of terminal methyl | C_24_H_31_N_3_O_4_ | 425.5206 |
| 89 | CC(C)(C)CCCn2nc(c1ccccc12)C(=O)NC(C)(C)c4ccc(O)cc4OC3OC  (C(O)C(O)C3O)C(=O)O | Human | Aromatic OH-glucuronidation | C_30_H_39_N_3_O_9_ | 585.6454 |
| 90 | CC(C)(C)CCCn2nc(c1ccccc12)C(=O)NC(C)(C)c3ccc(cc3O)OC4OC  (C(O)C(O)C4O)C(=O)O | Human | Aromatic OH-glucuronidation | C_30_H_39_N_3_O_9_ | 585.6454 |
| 91 | Oc1ccc(c(O)c1)C(C)(C)NC(=O)c3nn(CCCC(C)(C)C)c2c(O)cccc23 | Human | Aromatic hydroxylation of fused benzene ring | C_24_H_31_N_3_O_4_ | 425.5206 |
| 92 | Oc1ccc(c(O)c1)C(C)(C)NC(=O)c3nn(CCCC(C)(C)C)c2cccc(O)c23 | Human | Aromatic hydroxylation of fused benzene ring | C_24_H_31_N_3_O_4_ | 425.5206 |
| 93 | Oc1ccc(c(O)c1)C(C)(CO)NC(=O)c3nn(CCCC(C)(C)C)c2ccccc23 | Human | Hydroxylation of terminal methyl | C_24_H_31_N_3_O_4_ | 425.5206 |
| 94 | Oc1ccc(c(O)c1)C(C)(C)NC(=O)c3nn(CCCC(C)(C)CO)c2ccccc23 | Human | Hydroxylation of terminal methyl | C_24_H_31_N_3_O_4_ | 425.5206 |
| 95 | CC(C)(C)CCCn2nc(c1cccc(O)c12)C(=O)NC(C)(C)c4ccccc4OC3OC  (C(O)C(O)C3O)C(=O)O | Human | Aromatic OH-glucuronidation | C_30_H_39_N_3_O_9_ | 585.6454 |
| 96 | Oc1ccccc1C(C)(C)NC(=O)c4nn(CCCC(C)(C)C)c3c(OC2OC(C(O)C  (O)C2O)C(=O)O)cccc34 | Human | Aromatic OH-glucuronidation | C_30_H_39_N_3_O_9_ | 585.6454 |
| 97 | Oc1ccccc1C(C)(C)NC(=O)c3nn(CCCC(C)(C)C)c2c(O)c(O)ccc23 | Human | Aromatic hydroxylation of fused benzene ring | C_24_H_31_N_3_O_4_ | 425.5206 |
| 98 | Oc1ccccc1C(C)(C)NC(=O)c3nn(CCCC(C)(C)C)c2c(O)cc(O)cc23 | Human | Aromatic hydroxylation of fused benzene ring | C_24_H_31_N_3_O_4_ | 425.5206 |
| 99 | Oc1ccccc1C(C)(CO)NC(=O)c3nn(CCCC(C)(C)C)c2c(O)cccc23 | Human | Hydroxylation of terminal methyl | C_24_H_31_N_3_O_4_ | 425.5206 |
| 100 | Oc1ccccc1C(C)(C)NC(=O)c3nn(CCCC(C)(C)CO)c2c(O)cccc23 | Human | Hydroxylation of terminal methyl | C_24_H_31_N_3_O_4_ | 425.5206 |
| 101 | Oc1cccc(c1O)C(C)(C)NC(=O)c3nn(CCCC(C)(C)C)c2c(O)cccc23 | Human | O-Hydroxylation of phenol | C_24_H_31_N_3_O_4_ | 425.5206 |
| 102 | CC(C)(C)CCCn2nc(c1c(O)cccc12)C(=O)NC(C)(C)c4ccccc4OC3OC  (C(O)C(O)C3O)C(=O)O | Human | Aromatic OH-glucuronidation | C_30_H_39_N_3_O_9_ | 585.6454 |
| 103 | Oc1ccccc1C(C)(C)NC(=O)c4nn(CCCC(C)(C)C)c3cccc(OC2OC(C(O)  C(O)C2O)C(=O)O)c34 | Human | Aromatic OH-glucuronidation | C_30_H_39_N_3_O_9_ | 585.6454 |
| 104 | Oc1ccccc1C(C)(CO)NC(=O)c3nn(CCCC(C)(C)C)c2cccc(O)c23 | Human | Hydroxylation of terminal methyl | C_24_H_31_N_3_O_4_ | 425.5206 |
| 105 | Oc1ccccc1C(C)(C)NC(=O)c3nn(CCCC(C)(C)CO)c2cccc(O)c23 | Human | Hydroxylation of terminal methyl | C_24_H_31_N_3_O_4_ | 425.5206 |
| 106 | Oc1cccc(c1O)C(C)(C)NC(=O)c3nn(CCCC(C)(C)C)c2cccc(O)c23 | Human | O-Hydroxylation of phenol | C_24_H_31_N_3_O_4_ | 425.5206 |
| 107 | CC(C)(C)CCCn2nc(c1ccccc12)C(=O)NC(C)(CO)c4ccccc4OC3OC  (C(O)C(O)C3O)C(=O)O | Human | Aromatic OH-glucuronidation | C_30_H_39_N_3_O_9_ | 585.6454 |
| 108 | Oc1ccccc1C(C)(COS(=O)(=O)O)NC(=O)c3nn(CCCC(C)(C)C)c2  ccccc23 | Human | Sulfation of primary alcohol | C_24_H_31_N_3_O_6_S | 489.5844 |
| 109 | Oc1ccccc1C(C)(C=O)NC(=O)c3nn(CCCC(C)(C)C)c2ccccc23 | Human | Oxidation of primary alcohol to aldehyde | C_24_H_29_N_3_O_3_ | 407.5054 |
| 110 | Oc1ccccc1C(C)(CO)NC(=O)c3nn(CCCC(C)(C)CO)c2ccccc23 | Human | Hydroxylation of terminal methyl | C_24_H_31_N_3_O_4_ | 425.5206 |
| 111 | Oc1ccccc1C(CO)(CO)NC(=O)c3nn(CCCC(C)(C)C)c2ccccc23 | Human | Hydroxylation of terminal methyl | C_24_H_31_N_3_O_4_ | 425.5206 |
| 112 | Oc1cccc(c1O)C(C)(CO)NC(=O)c3nn(CCCC(C)(C)C)c2ccccc23 | Human | O-Hydroxylation of phenol | C_24_H_31_N_3_O_4_ | 425.5206 |
| 113 | CC(C)(CO)CCCn2nc(c1ccccc12)C(=O)NC(C)(C)c4ccccc4OC3OC  (C(O)C(O)C3O)C(=O)O | Human | Aromatic OH-glucuronidation | C_30_H_39_N_3_O_9_ | 585.6454 |
| 114 | Oc1ccccc1C(C)(C)NC(=O)c3nn(CCCC(C)(C)COS(=O)(=O)O)c2cc  ccc23 | Human | Sulfation of primary alcohol | C_24_H_31_N_3_O_6_S | 489.5844 |
| 115 | Oc1ccccc1C(C)(C)NC(=O)c3nn(CCCC(C)(C)C=O)c2ccccc23 | Human | Oxidation of primary alcohol to aldehyde | C_24_H_29_N_3_O_3_ | 407.5054 |
| 116 | Oc1ccccc1C(C)(CO)NC(=O)c3nn(CCCC(C)(C)CO)c2ccccc23 | Human | Hydroxylation of terminal methyl | C_24_H_31_N_3_O_4_ | 425.5206 |
| 117 | Oc1ccccc1C(C)(C)NC(=O)c3nn(CCCC(C)(CO)CO)c2ccccc23 | Human | Hydroxylation of terminal methyl | C_24_H_31_N_3_O_4_ | 425.5206 |
| 118 | Oc1cccc(c1O)C(C)(C)NC(=O)c3nn(CCCC(C)(C)CO)c2ccccc23 | Human | O-Hydroxylation of phenol | C_24_H_31_N_3_O_4_ | 425.5206 |
| 119 | CC(C)(C)CCCn2nc(c1ccccc12)C(=O)NC(C)(C)c4cccc(O)c4OC3OC  (C(O)C(O)C3O)C(=O)O | Human | Aromatic OH-glucuronidation | C_30_H_39_N_3_O_9_ | 585.6454 |
| 120 | CC(C)(C)CCCn2nc(c1ccccc12)C(=O)NC(C)(C)c4cccc(OC3OC(C(O)  C(O)C3O)C(=O)O)c4O | Human | Aromatic OH-glucuronidation | C_30_H_39_N_3_O_9_ | 585.6454 |
| 121 | Oc1ccc(O)c(O)c1C(C)(C)NC(=O)c3nn(CCCC(C)(C)C)c2ccccc23 | Human | Hydroxylation of benzene on carbon ortho to electron donating group | C_24_H_31_N_3_O_4_ | 425.5206 |
| 122 | CC(C)(NC(=O)c3nn(CCCC(C)(C)C)c2cc(OC1OC(C(O)C(O)C1O)C(=O)  O)c(O)cc23)c4ccccc4 | Human | Aromatic OH-glucuronidation | C_30_H_39_N_3_O_9_ | 585.6454 |
| 123 | CC(C)(NC(=O)c2nn(CCCC(C)(C)C)c3cc(O)c(OC1OC(C(O)C(O)C1O)  C(=O)O)cc23)c4ccccc4 | Human | Aromatic OH-glucuronidation | C_30_H_39_N_3_O_9_ | 585.6454 |
| 124 | Oc1ccc(cc1)C(C)(C)NC(=O)c3nn(CCCC(C)(C)C)c2cc(O)c(O)cc23 | Human | Hydroxylation of benzene on carbon para to electron donating group | C_24_H_31_N_3_O_4_ | 425.5206 |
| 125 | CC(C)(NC(=O)c2nn(CCCC(C)(C)C)c1cc(O)c(O)c(O)c12)c3ccccc3 | Human | Aromatic hydroxylation of fused benzene ring | C_24_H_31_N_3_O_4_ | 425.5206 |
| 126 | CC(CO)(NC(=O)c2nn(CCCC(C)(C)C)c1cc(O)c(O)cc12)c3ccccc3 | Human | Hydroxylation of terminal methyl | C_24_H_31_N_3_O_4_ | 425.5206 |
| 127 | CC(C)(NC(=O)c2nn(CCCC(C)(C)CO)c1cc(O)c(O)cc12)c3ccccc3 | Human | Hydroxylation of terminal methyl | C_24_H_31_N_3_O_4_ | 425.5206 |
| 128 | CC(C)(C)CCCn2nc(c1ccc(O)cc12)C(=O)NC(C)(C)c3ccc(cc3)OC4OC  (C(O)C(O)C4O)C(=O)O | Human | Aromatic OH-glucuronidation | C_30_H_39_N_3_O_9_ | 585.6454 |
| 129 | Oc1ccc(cc1)C(C)(C)NC(=O)c3nn(CCCC(C)(C)C)c4cc(OC2OC(C(O)C  (O)C2O)C(=O)O)ccc34 | Human | Aromatic OH-glucuronidation | C_30_H_39_N_3_O_9_ | 585.6454 |
| 130 | Oc1ccc(cc1O)C(C)(C)NC(=O)c3nn(CCCC(C)(C)C)c2cc(O)ccc23 | Human | 2-Hydroxylation of 1,4-disubstituted benzene | C_24_H_31_N_3_O_4_ | 425.5206 |
| 131 | Oc1ccc(cc1)C(C)(C)NC(=O)c3nn(CCCC(C)(C)C)c2cc(O)cc(O)c23 | Human | Aromatic hydroxylation of fused benzene ring | C_24_H_31_N_3_O_4_ | 425.5206 |
| 132 | Oc1ccc(cc1)C(C)(CO)NC(=O)c3nn(CCCC(C)(C)C)c2cc(O)ccc23 | Human | Hydroxylation of terminal methyl | C_24_H_31_N_3_O_4_ | 425.5206 |
| 133 | Oc1ccc(cc1)C(C)(C)NC(=O)c3nn(CCCC(C)(C)CO)c2cc(O)ccc23 | Human | Hydroxylation of terminal methyl | C_24_H_31_N_3_O_4_ | 425.5206 |
| 134 | CC(C)(NC(=O)c2nn(CCCC(C)(C)C)c1cc(cc(O)c12)OC3OC(C(O)C(O)C3O)  C(=O)O)c4ccccc4 | Human | Aromatic OH-glucuronidation | C_30_H_39_N_3_O_9_ | 585.6454 |
| 135 | CC(C)(NC(=O)c3nn(CCCC(C)(C)C)c2cc(O)cc(OC1OC(C(O)C(O)C1O)C  (=O)O)c23)c4ccccc4 | Human | Aromatic OH-glucuronidation | C_30_H_39_N_3_O_9_ | 585.6454 |
| 136 | CC(CO)(NC(=O)c2nn(CCCC(C)(C)C)c1cc(O)cc(O)c12)c3ccccc3 | Human | Hydroxylation of terminal methyl | C_24_H_31_N_3_O_4_ | 425.5206 |
| 137 | CC(C)(NC(=O)c2nn(CCCC(C)(C)CO)c1cc(O)cc(O)c12)c3ccccc3 | Human | Hydroxylation of terminal methyl | C_24_H_31_N_3_O_4_ | 425.5206 |
| 138 | CC(CO)(NC(=O)c2nn(CCCC(C)(C)C)c3cc(OC1OC(C(O)C(O)C1O)C(=O)  O)ccc23)c4ccccc4 | Human | Aromatic OH-glucuronidation | C_30_H_39_N_3_O_9_ | 585.6454 |
| 139 | O=S(=O)(O)OCC(C)(NC(=O)c2nn(CCCC(C)(C)C)c1cc(O)ccc12)c3cc  ccc3 | Human | Sulfation of primary alcohol | C_24_H_31_N_3_O_6_S | 489.5844 |
| 140 | O=CC(C)(NC(=O)c2nn(CCCC(C)(C)C)c1cc(O)ccc12)c3ccccc3 | Human | Oxidation of primary alcohol to aldehyde | C_24_H_29_N_3_O_3_ | 407.5054 |
| 141 | CC(CO)(NC(=O)c2nn(CCCC(C)(C)CO)c1cc(O)ccc12)c3ccccc3 | Human | Hydroxylation of terminal methyl | C_24_H_31_N_3_O_4_ | 425.5206 |
| 142 | OCC(CO)(NC(=O)c2nn(CCCC(C)(C)C)c1cc(O)ccc12)c3ccccc3 | Human | Hydroxylation of terminal methyl | C_24_H_31_N_3_O_4_ | 425.5206 |
| 143 | CC(C)(NC(=O)c2nn(CCCC(C)(C)CO)c3cc(OC1OC(C(O)C(O)C1O)C(  =O)O)ccc23)c4ccccc4 | Human | Aromatic OH-glucuronidation | C_30_H_39_N_3_O_9_ | 585.6454 |
| 144 | CC(C)(NC(=O)c2nn(CCCC(C)(C)COS(=O)(=O)O)c1cc(O)ccc12)c3c  cccc3 | Human | Sulfation of primary alcohol | C_24_H_31_N_3_O_6_S | 489.5844 |
| 145 | CC(C)(NC(=O)c2nn(CCCC(C)(C)C=O)c1cc(O)ccc12)c3ccccc3 | Human | Oxidation of primary alcohol to aldehyde | C_24_H_29_N_3_O_3_ | 407.5054 |
| 146 | CC(CO)(NC(=O)c2nn(CCCC(C)(C)CO)c1cc(O)ccc12)c3ccccc3 | Human | Hydroxylation of terminal methyl | C_24_H_31_N_3_O_4_ | 425.5206 |
| 147 | CC(C)(NC(=O)c2nn(CCCC(C)(CO)CO)c1cc(O)ccc12)c3ccccc3 | Human | Hydroxylation of terminal methyl | C_24_H_31_N_3_O_4_ | 425.5206 |
| 148 | CC(C)(C)CCCn2nc(c1cc(O)ccc12)C(=O)NC(C)(C)c3ccc(cc3)OC4OC  (C(O)C(O)C4O)C(=O)O | Human | Aromatic OH-glucuronidation | C_30_H_39_N_3_O_9_ | 585.6454 |
| 149 | Oc1ccc(cc1)C(C)(C)NC(=O)c3nn(CCCC(C)(C)C)c4ccc(OC2OC(C(O)  C(O)C2O)C(=O)O)cc34 | Human | Aromatic OH-glucuronidation | C_30_H_39_N_3_O_9_ | 585.6454 |
| 150 | Oc1ccc(cc1O)C(C)(C)NC(=O)c3nn(CCCC(C)(C)C)c2ccc(O)cc23 | Human | 2-Hydroxylation of 1,4-disubstituted benzene | C_24_H_31_N_3_O_4_ | 425.5206 |
| 151 | Oc1ccc(cc1)C(C)(C)NC(=O)c3nn(CCCC(C)(C)C)c2c(O)cc(O)cc23 | Human | Aromatic hydroxylation of fused benzene ring | C_24_H_31_N_3_O_4_ | 425.5206 |
| 152 | Oc1ccc(cc1)C(C)(C)NC(=O)c3nn(CCCC(C)(C)C)c2ccc(O)c(O)c23 | Human | Aromatic hydroxylation of fused benzene ring | C_24_H_31_N_3_O_4_ | 425.5206 |
| 153 | Oc1ccc(cc1)C(C)(CO)NC(=O)c3nn(CCCC(C)(C)C)c2ccc(O)cc23 | Human | Hydroxylation of terminal methyl | C_24_H_31_N_3_O_4_ | 425.5206 |
| 154 | Oc1ccc(cc1)C(C)(C)NC(=O)c3nn(CCCC(C)(C)CO)c2ccc(O)cc23 | Human | Hydroxylation of terminal methyl | C_24_H_31_N_3_O_4_ | 425.5206 |
| 155 | CC(C)(NC(=O)c3nn(CCCC(C)(C)C)c2c(OC1OC(C(O)C(O)C1O)C(=O)  O)cc(O)cc23)c4ccccc4 | Human | Aromatic OH-glucuronidation | C_30_H_39_N_3_O_9_ | 585.6454 |
| 156 | CC(C)(NC(=O)c3nn(CCCC(C)(C)C)c1c3cc(cc1O)OC2OC(C(O)C(O)C2O)  C(=O)O)c4ccccc4 | Human | Aromatic OH-glucuronidation | C_30_H_39_N_3_O_9_ | 585.6454 |
| 157 | CC(CO)(NC(=O)c2nn(CCCC(C)(C)C)c1c(O)cc(O)cc12)c3ccccc3 | Human | Hydroxylation of terminal methyl | C_24_H_31_N_3_O_4_ | 425.5206 |
| 158 | CC(C)(NC(=O)c2nn(CCCC(C)(C)CO)c1c(O)cc(O)cc12)c3ccccc3 | Human | Hydroxylation of terminal methyl | C_24_H_31_N_3_O_4_ | 425.5206 |
| 159 | CC(C)(NC(=O)c3nn(CCCC(C)(C)C)c2ccc(OC1OC(C(O)C(O)C1O)C(=O)  O)c(O)c23)c4ccccc4 | Human | Aromatic OH-glucuronidation | C_30_H_39_N_3_O_9_ | 585.6454 |
| 160 | CC(C)(NC(=O)c2nn(CCCC(C)(C)C)c3ccc(O)c(OC1OC(C(O)C(O)C1O)C  (=O)O)c23)c4ccccc4 | Human | Aromatic OH-glucuronidation | C_30_H_39_N_3_O_9_ | 585.6454 |
| 161 | CC(CO)(NC(=O)c2nn(CCCC(C)(C)C)c1ccc(O)c(O)c12)c3ccccc3 | Human | Hydroxylation of terminal methyl | C_24_H_31_N_3_O_4_ | 425.5206 |
| 162 | CC(C)(NC(=O)c2nn(CCCC(C)(C)CO)c1ccc(O)c(O)c12)c3ccccc3 | Human | Hydroxylation of terminal methyl | C_24_H_31_N_3_O_4_ | 425.5206 |
| 163 | CC(CO)(NC(=O)c2nn(CCCC(C)(C)C)c3ccc(OC1OC(C(O)C(O)C1O)C  (=O)O)cc23)c4ccccc4 | Human | Aromatic OH-glucuronidation | C_30_H_39_N_3_O_9_ | 585.6454 |
| 164 | O=S(=O)(O)OCC(C)(NC(=O)c2nn(CCCC(C)(C)C)c1ccc(O)cc12)c3cc  ccc3 | Human | Sulfation of primary alcohol | C_24_H_31_N_3_O_6_S | 489.5844 |
| 165 | O=CC(C)(NC(=O)c2nn(CCCC(C)(C)C)c1ccc(O)cc12)c3ccccc3 | Human | Oxidation of primary alcohol to aldehyde | C_24_H_29_N_3_O_3_ | 407.5054 |
| 166 | CC(CO)(NC(=O)c2nn(CCCC(C)(C)CO)c1ccc(O)cc12)c3ccccc3 | Human | Hydroxylation of terminal methyl | C_24_H_31_N_3_O_4_ | 425.5206 |
| 167 | OCC(CO)(NC(=O)c2nn(CCCC(C)(C)C)c1ccc(O)cc12)c3ccccc3 | Human | Hydroxylation of terminal methyl | C_24_H_31_N_3_O_4_ | 425.5206 |
| 168 | CC(C)(NC(=O)c2nn(CCCC(C)(C)CO)c3ccc(OC1OC(C(O)C(O)C1O)C  (=O)O)cc23)c4ccccc4 | Human | Aromatic OH-glucuronidation | C_30_H_39_N_3_O_9_ | 585.6454 |
| 169 | CC(C)(NC(=O)c2nn(CCCC(C)(C)COS(=O)(=O)O)c1ccc(O)cc12)c3c  cccc3 | Human | Sulfation of primary alcohol | C_24_H_31_N_3_O_6_S | 489.5844 |
| 170 | CC(C)(NC(=O)c2nn(CCCC(C)(C)C=O)c1ccc(O)cc12)c3ccccc3 | Human | Oxidation of primary alcohol to aldehyde | C_24_H_29_N_3_O_3_ | 407.5054 |
| 171 | CC(CO)(NC(=O)c2nn(CCCC(C)(C)CO)c1ccc(O)cc12)c3ccccc3 | Human | Hydroxylation of terminal methyl | C_24_H_31_N_3_O_4_ | 425.5206 |
| 172 | CC(C)(NC(=O)c2nn(CCCC(C)(CO)CO)c1ccc(O)cc12)c3ccccc3 | Human | Hydroxylation of terminal methyl | C_24_H_31_N_3_O_4_ | 425.5206 |
| 173 | Oc1ccc(cc1OC)C(C)(C)NC(=O)c3nn(CCCC(C)(C)C)c2ccccc23 | Human | Catechol O-methylation | C_25_H_33_N_3_O_3_ | 423.5478 |
| 174 | COc1ccc(cc1O)C(C)(C)NC(=O)c3nn(CCCC(C)(C)C)c2ccccc23 | Human | Catechol O-methylation | C_25_H_33_N_3_O_3_ | 423.5478 |
| 175 | CC(C)(C)CCCn2nc(c1ccccc12)C(=O)NC(C)(C)c4ccc(OC3OC(C(O)  C(O)C3O)C(=O)O)c(O)c4 | Human | Aromatic OH-glucuronidation | C_30_H_39_N_3_O_9_ | 585.6454 |
| 176 | CC(C)(C)CCCn2nc(c1ccccc12)C(=O)NC(C)(C)c4cc(OC3OC(C(O)  C(O)C3O)C(=O)O)c(O)cc4 | Human | Aromatic OH-glucuronidation | C_30_H_39_N_3_O_9_ | 585.6454 |
| 177 | Oc1ccc(cc1O)C(C)(C)NC(=O)c3nn(CCCC(C)(C)C)c2c(O)cccc23 | Human | Aromatic hydroxylation of fused benzene ring | C_24_H_31_N_3_O_4_ | 425.5206 |
| 178 | Oc1ccc(cc1O)C(C)(C)NC(=O)c3nn(CCCC(C)(C)C)c2cccc(O)c23 | Human | Aromatic hydroxylation of fused benzene ring | C_24_H_31_N_3_O_4_ | 425.5206 |
| 179 | Oc1ccc(cc1O)C(C)(CO)NC(=O)c3nn(CCCC(C)(C)C)c2ccccc23 | Human | Hydroxylation of terminal methyl | C_24_H_31_N_3_O_4_ | 425.5206 |
| 180 | Oc1ccc(cc1O)C(C)(C)NC(=O)c3nn(CCCC(C)(C)CO)c2ccccc23 | Human | Hydroxylation of terminal methyl | C_24_H_31_N_3_O_4_ | 425.5206 |
| 181 | Oc1cccc(c1)C(C)(C)NC(=O)c3nn(CCCC(C)(C)C)c2ccccc23 | Gut microbial environment | Dehydroxylation of catechol | C_24_H_31_N_3_O_2_ | 393.5218 |
| 182 | CC(C)(C)CCCn2nc(c1cccc(O)c12)C(=O)NC(C)(C)c3ccc(cc3)OC4OC  (C(O)C(O)C4O)C(=O)O | Human | Aromatic OH-glucuronidation | C_30_H_39_N_3_O_9_ | 585.6454 |
| 183 | Oc1ccc(cc1)C(C)(C)NC(=O)c4nn(CCCC(C)(C)C)c3c(OC2OC(C(O)C  (O)C2O)C(=O)O)cccc34 | Human | Aromatic OH-glucuronidation | C_30_H_39_N_3_O_9_ | 585.6454 |
| 184 | Oc1ccc(cc1)C(C)(C)NC(=O)c3nn(CCCC(C)(C)C)c2c(O)c(O)ccc23 | Human | Aromatic hydroxylation of fused benzene ring | C_24_H_31_N_3_O_4_ | 425.5206 |
| 185 | Oc1ccc(cc1)C(C)(C)NC(=O)c3nn(CCCC(C)(C)C)c2c(O)cc(O)cc23 | Human | Aromatic hydroxylation of fused benzene ring | C_24_H_31_N_3_O_4_ | 425.5206 |
| 186 | Oc1ccc(cc1)C(C)(CO)NC(=O)c3nn(CCCC(C)(C)C)c2c(O)cccc23 | Human | Hydroxylation of terminal methyl | C_24_H_31_N_3_O_4_ | 425.5206 |
| 187 | Oc1ccc(cc1)C(C)(C)NC(=O)c3nn(CCCC(C)(C)CO)c2c(O)cccc23 | Human | Hydroxylation of terminal methyl | C_24_H_31_N_3_O_4_ | 425.5206 |
| 188 | CC(C)(C)CCCn2nc(c1c(O)cccc12)C(=O)NC(C)(C)c3ccc(cc3)OC4OC  (C(O)C(O)C4O)C(=O)O | Human | Aromatic OH-glucuronidation | C_30_H_39_N_3_O_9_ | 585.6454 |
| 189 | Oc1ccc(cc1)C(C)(C)NC(=O)c4nn(CCCC(C)(C)C)c3cccc(OC2OC(C(O)  C(O)C2O)C(=O)O)c34 | Human | Aromatic OH-glucuronidation | C_30_H_39_N_3_O_9_ | 585.6454 |
| 190 | Oc1ccc(cc1)C(C)(CO)NC(=O)c3nn(CCCC(C)(C)C)c2cccc(O)c23 | Human | Hydroxylation of terminal methyl | C_24_H_31_N_3_O_4_ | 425.5206 |
| 191 | Oc1ccc(cc1)C(C)(C)NC(=O)c3nn(CCCC(C)(C)CO)c2cccc(O)c23 | Human | Hydroxylation of terminal methyl | C_24_H_31_N_3_O_4_ | 425.5206 |
| 192 | CC(C)(C)CCCn2nc(c1ccccc12)C(=O)NC(C)(CO)c3ccc(cc3)OC4OC  (C(O)C(O)C4O)C(=O)O | Human | Aromatic OH-glucuronidation | C_30_H_39_N_3_O_9_ | 585.6454 |
| 193 | Oc1ccc(cc1)C(C)(COS(=O)(=O)O)NC(=O)c3nn(CCCC(C)(C)C)c2c  cccc23 | Human | Sulfation of primary alcohol | C_24_H_31_N_3_O_6_S | 489.5844 |
| 194 | Oc1ccc(cc1)C(C)(C=O)NC(=O)c3nn(CCCC(C)(C)C)c2ccccc23 | Human | Oxidation of primary alcohol to aldehyde | C_24_H_29_N_3_O_3_ | 407.5054 |
| 195 | Oc1ccc(cc1)C(C)(CO)NC(=O)c3nn(CCCC(C)(C)CO)c2ccccc23 | Human | Hydroxylation of terminal methyl | C_24_H_31_N_3_O_4_ | 425.5206 |
| 196 | Oc1ccc(cc1)C(CO)(CO)NC(=O)c3nn(CCCC(C)(C)C)c2ccccc23 | Human | Hydroxylation of terminal methyl | C_24_H_31_N_3_O_4_ | 425.5206 |
| 197 | CC(C)(CO)CCCn2nc(c1ccccc12)C(=O)NC(C)(C)c3ccc(cc3)OC4OC  (C(O)C(O)C4O)C(=O)O | Human | Aromatic OH-glucuronidation | C_30_H_39_N_3_O_9_ | 585.6454 |
| 198 | Oc1ccc(cc1)C(C)(C)NC(=O)c3nn(CCCC(C)(C)COS(=O)(=O)O)c2c  cccc23 | Human | Sulfation of primary alcohol | C_24_H_31_N_3_O_6_S | 489.5844 |
| 199 | Oc1ccc(cc1)C(C)(C)NC(=O)c3nn(CCCC(C)(C)C=O)c2ccccc23 | Human | Oxidation of primary alcohol to aldehyde | C_24_H_29_N_3_O_3_ | 407.5054 |
| 200 | Oc1ccc(cc1)C(C)(CO)NC(=O)c3nn(CCCC(C)(C)CO)c2ccccc23 | Human | Hydroxylation of terminal methyl | C_24_H_31_N_3_O_4_ | 425.5206 |
| 201 | Oc1ccc(cc1)C(C)(C)NC(=O)c3nn(CCCC(C)(CO)CO)c2ccccc23 | Human | Hydroxylation of terminal methyl | C_24_H_31_N_3_O_4_ | 425.5206 |
| 202 | CC(C)(NC(=O)c3nn(CCCC(C)(C)C)c2c(OC1OC(C(O)C(O)C1O)C(=O)  O)c(O)ccc23)c4ccccc4 | Human | Aromatic OH-glucuronidation | C_30_H_39_N_3_O_9_ | 585.6454 |
| 203 | CC(C)(NC(=O)c2nn(CCCC(C)(C)C)c3c(O)c(OC1OC(C(O)C(O)C1O)C  (=O)O)ccc23)c4ccccc4 | Human | Aromatic OH-glucuronidation | C_30_H_39_N_3_O_9_ | 585.6454 |
| 204 | CC(C)(NC(=O)c2nn(CCCC(C)(C)C)c1c(O)c(O)c(O)cc12)c3ccccc3 | Human | Aromatic hydroxylation of fused benzene ring | C_24_H_31_N_3_O_4_ | 425.5206 |
| 205 | CC(CO)(NC(=O)c2nn(CCCC(C)(C)C)c1c(O)c(O)ccc12)c3ccccc3 | Human | Hydroxylation of terminal methyl | C_24_H_31_N_3_O_4_ | 425.5206 |
| 206 | CC(C)(NC(=O)c2nn(CCCC(C)(C)CO)c1c(O)c(O)ccc12)c3ccccc3 | Human | Hydroxylation of terminal methyl | C_24_H_31_N_3_O_4_ | 425.5206 |
| 207 | CC(CO)(NC(=O)c3nn(CCCC(C)(C)C)c2c(OC1OC(C(O)C(O)C1O)C  (=O)O)cccc23)c4ccccc4 | Human | Aromatic OH-glucuronidation | C_30_H_39_N_3_O_9_ | 585.6454 |
| 208 | O=S(=O)(O)OCC(C)(NC(=O)c2nn(CCCC(C)(C)C)c1c(O)cccc12)c3c  cccc3 | Human | Sulfation of primary alcohol | C_24_H_31_N_3_O_6_S | 489.5844 |
| 209 | O=CC(C)(NC(=O)c2nn(CCCC(C)(C)C)c1c(O)cccc12)c3ccccc3 | Human | Oxidation of primary alcohol to aldehyde | C_24_H_29_N_3_O_3_ | 407.5054 |
| 210 | CC(CO)(NC(=O)c2nn(CCCC(C)(C)CO)c1c(O)cccc12)c3ccccc3 | Human | Hydroxylation of terminal methyl | C_24_H_31_N_3_O_4_ | 425.5206 |
| 211 | OCC(CO)(NC(=O)c2nn(CCCC(C)(C)C)c1c(O)cccc12)c3ccccc3 | Human | Hydroxylation of terminal methyl | C_24_H_31_N_3_O_4_ | 425.5206 |
| 212 | CC(C)(NC(=O)c3nn(CCCC(C)(C)CO)c2c(OC1OC(C(O)C(O)C1O)C(=O)  O)cccc23)c4ccccc4 | Human | Aromatic OH-glucuronidation | C_30_H_39_N_3_O_9_ | 585.6454 |
| 213 | CC(C)(NC(=O)c2nn(CCCC(C)(C)COS(=O)(=O)O)c1c(O)cccc12)c3cc  ccc3 | Human | Sulfation of primary alcohol | C_24_H_31_N_3_O_6_S | 489.5844 |
| 214 | CC(C)(NC(=O)c2nn(CCCC(C)(C)C=O)c1c(O)cccc12)c3ccccc3 | Human | Oxidation of primary alcohol to aldehyde | C_24_H_29_N_3_O_3_ | 407.5054 |
| 215 | CC(CO)(NC(=O)c2nn(CCCC(C)(C)CO)c1c(O)cccc12)c3ccccc3 | Human | Hydroxylation of terminal methyl | C_24_H_31_N_3_O_4_ | 425.5206 |
| 216 | CC(C)(NC(=O)c2nn(CCCC(C)(CO)CO)c1c(O)cccc12)c3ccccc3 | Human | Hydroxylation of terminal methyl | C_24_H_31_N_3_O_4_ | 425.5206 |
| 217 | CC(CO)(NC(=O)c3nn(CCCC(C)(C)C)c2cccc(OC1OC(C(O)C(O)C1O)C  (=O)O)c23)c4ccccc4 | Human | Aromatic OH-glucuronidation | C_30_H_39_N_3_O_9_ | 585.6454 |
| 218 | O=S(=O)(O)OCC(C)(NC(=O)c2nn(CCCC(C)(C)C)c1cccc(O)c12)c3c  cccc3 | Human | Sulfation of primary alcohol | C_24_H_31_N_3_O_6_S | 489.5844 |
| 219 | O=CC(C)(NC(=O)c2nn(CCCC(C)(C)C)c1cccc(O)c12)c3ccccc3 | Human | Oxidation of primary alcohol to aldehyde | C_24_H_29_N_3_O_3_ | 407.5054 |
| 220 | CC(CO)(NC(=O)c2nn(CCCC(C)(C)CO)c1cccc(O)c12)c3ccccc3 | Human | Hydroxylation of terminal methyl | C_24_H_31_N_3_O_4_ | 425.5206 |
| 221 | OCC(CO)(NC(=O)c2nn(CCCC(C)(C)C)c1cccc(O)c12)c3ccccc3 | Human | Hydroxylation of terminal methyl | C_24_H_31_N_3_O_4_ | 425.5206 |
| 222 | CC(C)(NC(=O)c3nn(CCCC(C)(C)CO)c2cccc(OC1OC(C(O)C(O)C1O)  C(=O)O)c23)c4ccccc4 | Human | Aromatic OH-glucuronidation | C_30_H_39_N_3_O_9_ | 585.6454 |
| 223 | CC(C)(NC(=O)c2nn(CCCC(C)(C)COS(=O)(=O)O)c1cccc(O)c12)c3c  cccc3 | Human | Sulfation of primary alcohol | C_24_H_31_N_3_O_6_S | 489.5844 |
| 224 | CC(C)(NC(=O)c2nn(CCCC(C)(C)C=O)c1cccc(O)c12)c3ccccc3 | Human | Oxidation of primary alcohol to aldehyde | C_24_H_29_N_3_O_3_ | 407.5054 |
| 225 | CC(CO)(NC(=O)c2nn(CCCC(C)(C)CO)c1cccc(O)c12)c3ccccc3 | Human | Hydroxylation of terminal methyl | C_24_H_31_N_3_O_4_ | 425.5206 |
| 226 | CC(C)(NC(=O)c2nn(CCCC(C)(CO)CO)c1cccc(O)c12)c3ccccc3 | Human | Hydroxylation of terminal methyl | C_24_H_31_N_3_O_4_ | 425.5206 |
| 227 | O=C(O)C(C)(NC(=O)c2nn(CCCC(C)(C)C)c1ccccc12)c3ccccc3 | Human | Aldehyde oxidation | C_24_H_29_N_3_O_3_ | 407.5054 |
| 228 | O=CC(C)(NC(=O)c2nn(CCCC(C)(C)CO)c1ccccc12)c3ccccc3 | Human | Hydroxylation of terminal methyl | C_24_H_29_N_3_O_3_ | 407.5054 |
| 229 | O=CC(CO)(NC(=O)c2nn(CCCC(C)(C)C)c1ccccc12)c3ccccc3 | Human | Hydroxylation of terminal methyl | C_24_H_29_N_3_O_3_ | 407.5054 |
| 230 | CC(C)(CO)CCCn2nc(c1ccccc12)C(=O)NC(C)(COC3OC(C(O)C(O)  C3O)C(=O)O)c4ccccc4 | Human | Alkyl-OH-glucuronidation | C_30_H_39_N_3_O_9_ | 585.6454 |
| 231 | CC(CO)(NC(=O)c3nn(CCCC(C)(C)COC1OC(C(O)C(O)C1O)C(=O)O)  c2ccccc23)c4ccccc4 | Human | Alkyl-OH-glucuronidation | C_30_H_39_N_3_O_9_ | 585.6454 |
| 232 | O=S(=O)(O)OCC(C)(NC(=O)c2nn(CCCC(C)(C)CO)c1ccccc12)c3cc  ccc3 | Human | Sulfation of primary alcohol | C_24_H_31_N_3_O_6_S | 489.5844 |
| 233 | CC(CO)(NC(=O)c2nn(CCCC(C)(C)COS(=O)(=O)O)c1ccccc12)c3cc  ccc3 | Human | Sulfation of primary alcohol | C_24_H_31_N_3_O_6_S | 489.5844 |
| 234 | O=CC(C)(NC(=O)c2nn(CCCC(C)(C)CO)c1ccccc12)c3ccccc3 | Human | Oxidation of primary alcohol to aldehyde | C_24_H_29_N_3_O_3_ | 407.5054 |
| 235 | CC(CO)(NC(=O)c2nn(CCCC(C)(C)C=O)c1ccccc12)c3ccccc3 | Human | Oxidation of primary alcohol to aldehyde | C_24_H_29_N_3_O_3_ | 407.5054 |
| 236 | CC(CO)(NC(=O)c2nn(CCCC(C)(CO)CO)c1ccccc12)c3ccccc3 | Human | Hydroxylation of terminal methyl | C_24_H_31_N_3_O_4_ | 425.5206 |
| 237 | OCC(CO)(NC(=O)c2nn(CCCC(C)(C)CO)c1ccccc12)c3ccccc3 | Human | Hydroxylation of terminal methyl | C_24_H_31_N_3_O_4_ | 425.5206 |
| 238 | CC(C)(C)CCCn2nc(c1ccccc12)C(=O)NC(COC3OC(C(O)C(O)C3O)  C(=O)O)(CO)c4ccccc4 | Human | Alkyl-OH-glucuronidation | C_30_H_39_N_3_O_9_ | 585.6454 |
| 239 | O=S(=O)(O)OCC(CO)(NC(=O)c2nn(CCCC(C)(C)C)c1ccccc12)c3c  cccc3 | Human | Sulfation of primary alcohol | C_24_H_31_N_3_O_6_S | 489.5844 |
| 240 | CC(C)(NC(=O)c2nn(CCCC(C)(C)C(=O)O)c1ccccc12)c3ccccc3 | Human | Aldehyde oxidation | C_24_H_29_N_3_O_3_ | 407.5054 |
| 241 | CC(CO)(NC(=O)c2nn(CCCC(C)(C)C=O)c1ccccc12)c3ccccc3 | Human | Hydroxylation of terminal methyl | C_24_H_29_N_3_O_3_ | 407.5054 |
| 242 | CC(C)(NC(=O)c2nn(CCCC(C)(C=O)CO)c1ccccc12)c3ccccc3 | Human | Hydroxylation of terminal methyl | C_24_H_29_N_3_O_3_ | 407.5054 |
| 243 | CC(C)(NC(=O)c3nn(CCCC(C)(COC1OC(C(O)C(O)C1O)C(=O)O)  CO)c2ccccc23)c4ccccc4 | Human | Alkyl-OH-glucuronidation | C_30_H_39_N_3_O_9_ | 585.6454 |
| 244 | CC(C)(NC(=O)c2nn(CCCC(C)(CO)COS(=O)(=O)O)c1ccccc12)c3c  cccc3 | Human | Sulfation of primary alcohol | C_24_H_31_N_3_O_6_S | 489.5844 |
| 245 | CC(CO)(NC(=O)c2nn(CCCC(C)(CO)CO)c1ccccc12)c3ccccc3 | Human | Hydroxylation of terminal methyl | C_24_H_31_N_3_O_4_ | 425.5206 |
| 246 | CC(C)(NC(=O)c2nn(CCCC(CO)(CO)CO)c1ccccc12)c3ccccc3 | Human | Hydroxylation of terminal methyl | C_24_H_31_N_3_O_4_ | 425.5206 |

**Table S10** *In silico* metabolite predictions with GLORYx for the analogue of ADMB-3TMS-PrINACA with Si-C exchange (“**ADMB-4,4-dimethyl-PINACA”**) with structures, the ranks with the appropriate calculated priority scores, the reaction type, the formula and formula weight (FW)

| **ID** | **Structure(SMILES)** | **Rank** | **Priority Score** | **Reaction Type** | **Formula** | **FW** |
| --- | --- | --- | --- | --- | --- | --- |
| 1 | NC(=O)C(NC(=O)c2nn(CCCC(C)(C)C)c1ccccc12)C(C)(C)C | - | - | - | C_21_H_32_N_4_O_2_ | 372_._5044 |
| 2 | O=C(N)C(NC(=O)c2nn(c1ccccc12)CCCC(C)(C)CO)C(C)(C)C | 1 | 0.372 | aliphatic hydroxylation | C_21_H_32_N_4_O_3_ | 388_._5038 |
| 3 | O=C(NO)C(NC(=O)c2nn(c1ccccc12)CCCC(C)(C)C)C(C)(C)C | 2 | 0.32 | amine hydroxylation | C_21_H_32_N_4_O_3_ | 388.5038 |
| 4 | O=C(O)C(NC(=O)c2nn(c1ccccc12)CCCC(C)(C)C)C(C)(C)C | 2 | 0.32 | hydrolysis_(primary_amide) | C_21_H_31_N_3_O_3_ | 373.4891 |
| 5 | O=C(N)C(O)(NC(=O)c2nn(c1ccccc12)CCCC(C)(C)C)C(C)(C)C | 2 | 0.32 | aliphatic_hydroxylation_(tert_carbon_next_to_SP^2^) | C_21_H_32_N_4_O_3_ | 388.5038 |
| 6 | O=C(N)C(NC(=O)c2nn(c1ccccc12)CCC(O)C(C)(C)C)C(C)(C)C | 5 | 0.292 | aliphatic hydroxylation | C_21_H_32_N_4_O_3_ | 388.5038 |
| _7_ | O=C(N)C(NC(=O)c2nn(c1ccccc12)CCCC(C)(C)C)C(C)(C)CO | 6 | 0.288 | aliphatic hydroxylation | C_21_H_32_N_4_O_3_ | 388.5038 |
| 8 | O=C(O)c2nn(c1ccccc12)CCCC(C)(C)C | 7 | 0.252 | hydrolysis_(secondary_amide) | C_15_H_20_N_2_O_2_ | 260.3315 |
| 9 | O=C(N)C(N(O)C(=O)c2nn(c1ccccc12)CCCC(C)(C)C)C(C)(C)C | 7 | 0.252 | amine hydroxylation | C_21_H_32_N_4_O_3_ | 388.5038 |
| 10 | O=C(N)C(N)C(C)(C)C | 7 | 0.252 | hydrolysis_(secondary_amide) | C_6_H_14_N_2_O | 130.1882 |
| 11 | O=C(NC1OC(C(=O)O)C(O)C(O)C1O)C(NC(=O)c3nn(c2ccccc23)CCCC(C)(C)C)C(C)(C)C | 10 | 0.231 | *N*-glucuronidation_(aliphatic_NH_2_) | C_27_H_40_N_4_O_8_ | 548.6285 |
| 12 | O=C(c2nn(c1ccccc12)CCCC(C)(C)C)N | 11 | 0.128 | *N*-dealkylation | C_15_H_21_N_3_O | 259.3467 |
| 13 | O=C(C(=O)N)C(C)(C)C | 11 | 0.128 | *N*-dealkylation | C_6_H_11_NO_2_ | 129.1570 |
| 14 | O=C(N)C(NC(=O)c2nn(c1ccccc12)C(O)CCC(C)(C)C)C(C)(C)C | 13 | 0.124 | aliphatic hydroxylation | C_21_H_32_N_4_O_3_ | 388.5038 |
| 15 | OCCCC(C)(C)C | 13 | 0.124 | *N*-dealkylation_(nCH_2_) | C_7_H_16_O | 116.2013 |
| 16 | O=C(N)C(NC(=O)c2n[n+]([O-])(c1ccccc12)CCCC(C)(C)C)C(C)(C)C | 13 | 0.124 | *N*-oxidation | C_21_H_32_N_4_O_3_ | 388.5038 |
| 17 | O=CCCC(C)(C)C | 13 | 0.124 | *N*-dealkylation | C_7_H_14_O | 114.1855 |
| 18 | O=C(N)C(NC(=O)c2nnc1ccccc12)C(C)(C)C | 13 | 0.124 | *N*-dealkylation_(nCH_2_) | C_14_H_18_N_4_O_2_ | 274.3183 |
| 19 | O=C(N)C(NC(=O)c2nn(c1cc(O)ccc12)CCCC(C)(C)C)C(C)(C)C | 18 | 0.116 | aromatic_hydroxylation_(para_to_carbon) | C_21_H_32_N_4_O_3_ | 388.5038 |
| 20 | O=C(N)C(NC(=O)c2nn(c1ccc(O)cc12)CCCC(C)(C)C)C(C)(C)C | 18 | 0.116 | aromatic_hydroxylation_(para_to_nitrogen) | C_21_H_32_N_4_O_3_ | 388.5038 |
| 21 | O=C(N)C(NC(=O)c2nn(c1c(O)cccc12)CCCC(C)(C)C)C(C)(C)C | 18 | 0.116 | aromatic_hydroxylation_(ortho_to_nitrogen) | C_21_H_32_N_4_O_3_ | 388.5038 |
| 22 | O=C(N)C(NC(=O)c2[n+](n(c1ccccc12)CCCC(C)(C)C)C3OC(C(=O)O)C(O)C(O)C3O)C(C)(C)C | _21_ | 0.060 | *N*-glucuronidation_(aromatic_=n-) | C_27_H4_1_N_4_O_8_ | 549.6359 |
| 23 | O=C(N)C(NC(=O)c2nn(c1ccccc12)C/C=C/C(C)(C)C)C(C)(C)C | 22 | 0.058 | alkyl dehydrogenation | C_21_H_30_N_4_O_2_ | 370.4885 |
| 24 | O=C(N)C(NC(=O)c2nn(c1cccc(O)c12)CCCC(C)(C)C)C(C)(C)C | 23 | 0.036 | aromatic hydroxylation | C_21_H_32_N_4_O_3_ | 388.5038 |
| 25 | O=C(N)C(NC(=O)c1[n+]([O-])n(c2ccccc12)CCCC(C)(C)C)C(C)(C)C | 23 | 0.036 | *N*-oxidation | C_21_H_32_N_4_O_3_ | 388.5038 |
| 26 | O=C(N)C(NC(=O)c2nn(c1ccccc12)CC(O)CC(C)(C)C)C(C)(C)C | 23 | 0.036 | aliphatic hydroxylation | C_21_H_32_N_4_O_3_ | 388.5038 |
| 27 | O=C(N)\C(=[NH+]/C(=O)c2nn(c1ccccc12)CCCC(C)(C)C)C(C)(C)C | 26 | 0.0256 | dehydration of *N*-*C* bond | C_21_H_31_N_4_O_2_ | 371.4959 |
| 28 | O=C(N)\C(=N/C(=O)c2nn(c1ccccc12)CCCC(C)(C)C)C(C)(C)C | 26 | 0.0256 | dehydration of *N*-*C* bond | C_21_H_30_N_4_O_2_ | 370.4885 |
| 29 | O=C(N)C(NC(=O)c2nn(c1ccccc12)/C=C/CC(C)(C)C)C(C)(C)C | 28 | 0.0248 | alkyl dehydrogenation | C_21_H_30_N_4_O_2_ | 370.4885 |

**Table S11** *In silico* metabolite predictions with GLORYx for the analogue of Cumyl-3TMS-PrINACA with Si-C exchange (“**Cumyl-4,4-dimethyl-PINACA”**) with structures, the ranks with the appropriate calculated priority scores, the reaction type, the formula and formula weight (FW)

| **ID** | **Structure (SMILES)** | **Rank** | **Priority**  **Score** | | **Reaction Type** | **Formula** | **FW** |  |  |  |
| --- | --- | --- | --- | --- | --- | --- | --- | --- | --- | --- |
| 1 | CC(C)(NC(=O)c2nn(CCCC(C)(C)C)c1ccccc12)c3ccccc3 |  | |  | parent | | | | C24H31N3O | 3.775.224 |
| 2 | O=C(c2nn(c1ccccc12)CCCC(C)(C)CO)NC(c3ccccc3)(C)C | 1 | 0.444 | | aliphatic hydroxylation | C_24_H_31_N_3_O_2_ | 393.5218 |  |  |  |
| 3 | O=C(c2nn(c1ccccc12)CCC(O)C(C)(C)C)NC(c3ccccc3)(C)C | 2 | 0.292 | | aliphatic hydroxylation | C_24_H_31_N_3_O_2_ | 393.5218 |  |  |  |
| 4 | O=C(c2nn(c1ccccc12)CCCC(C)(C)C)NC(c3cccc(O)c3)(C)C | 3 | 0.224 | | aromatic_hydroxylation_(meta_to_carbon) | C_24_H_31_N_3_O_2_ | 393.5218 |  |  |  |
| 5 | O=C(c2nn(c1ccccc12)CCCC(C)(C)C)NC(c3ccc(O)c(OC)c3)(C)C | 3 | 0.224 | | aromatic_oxidation | C_25_H_33_N_3_O_3_ | 423.5478 |  |  |  |
| 6 | O=C(c2nn(c1ccccc12)CCCC(C)(C)C)NC(c3ccc(O)cc3)(C)C | 3 | 0.224 | | aromatic_hydroxylation_(para_to_carbon) | C_24_H_31_N_3_O_2_ | 393.5218 |  |  |  |
| 7 | O=C(c2nn(c1ccccc12)CCCC(C)(C)C)N(O)C(c3ccccc3)(C)C | 6 | 0.204 | | amine hydroxylation | C_24_H_31_N_3_O_2_ | 393.5218 |  |  |  |
| 8 | NC(c1ccccc1)(C)C | 6 | 0.204 | | hydrolysis_(secondary_amide) | C_9_H_13_N | 135.2062 |  |  |  |
| 9 | O=C(O)c2nn(c1ccccc12)CCCC(C)(C)C | 6 | 0.204 | | hydrolysis_(secondary_amide) | C_15_H_20_N_2_O_2_ | 260.3315 |  |  |  |
| 10 | O=C(c2nn(c1ccccc12)CCCC(C)(C)C)NC(c3ccccc3)(C)CO | 9 | 0.148 | | aliphatic_hydroxylation_(primary_carbon_next_to_quart_carbon) | C_24_H_31_N_3_O_2_ | 393.5218 |  |  |  |
| 11 | O=C(O)C(c1ccccc1)(NC(=O)c3nn(c2ccccc23)CCCC(C)(C)C)C | 9 | 0.148 | | carboxylation_(primary_carbon_next_to_quart_carbon) | C_24_H_29_N_3_O_3_ | 407.5054 |  |  |  |
| 12 | O=C(c2nn(c1cc(O)ccc12)CCCC(C)(C)C)NC(c3ccccc3)(C)C | 11 | 0.136 | | aromatic_hydroxylation_(para_to_carbon) | C_24_H_31_N_3_O_2_ | 393.5218 |  |  |  |
| 13 | O=C(c2nn(c1c(O)cccc12)CCCC(C)(C)C)NC(c3ccccc3)(C)C | 11 | 0.136 | | aromatic_hydroxylation_(ortho_to_nitrogen) | C_24_H_31_N_3_O_2_ | 393.5218 |  |  |  |
| 14 | O=C(c2nn(c1ccc(O)cc12)CCCC(C)(C)C)NC(c3ccccc3)(C)C | 11 | 0.136 | | aromatic_hydroxylation_(para_to_nitrogen) | C_24_H_31_N_3_O_2_ | 393.5218 |  |  |  |
| 15 | [O-][n+]2(nc(c1ccccc12)C(=O)NC(c3ccccc3)(C)C)CCCC(C)(C)C | 14 | 0.100 | | *N*-oxidation | C_24_H_31_N_3_O_2_ | 393.5218 |  |  |  |
| 16 | O=CCCC(C)(C)C | 14 | 0.100 | | *N*-dealkylation | C_7_H_14_O | 114.1855 |  |  |  |
| 17 | OCCCC(C)(C)C | 14 | 0.100 | | *N*-dealkylation_(nCH_2_) | C_7_H_16_O | 116.2013 |  |  |  |
| 18 | O=C(c2nnc1ccccc12)NC(c3ccccc3)(C)C | 14 | 0.100 | | *N*-dealkylation_(nCH_2_) | C_17_H_17_N_3_O | 279.3364 |  |  |  |
| 19 | O=C(c2nn(c1ccccc12)C(O)CCC(C)(C)C)NC(c3ccccc3)(C)C | 14 | 0.100 | | aliphatic hydroxylation | C_24_H_31_N_3_O_2_ | 393.5218 |  |  |  |
| 20 | O=C(c2nn(c1ccccc12)C/C=C/C(C)(C)C)NC(c3ccccc3)(C)C | 19 | 0.058 | | alkyl dehydrogenation | C_24_H_29_N_3_O | 375.5066 |  |  |  |
| 21 | O=C(O)C4OC([n+]2n(c1ccccc1c2C(=O)NC(c3ccccc3)(C)C)CCCC  (C)(C)C)C(O)C(O)C4O | 20 | 0.048 | | *N*-glucuronidation_(aromatic_=n-) | C_30_H_40_N_3_O_7_ | 554.6540 |  |  |  |
| 22 | O=C(c2nn(c1cccc(O)c12)CCCC(C)(C)C)NC(c3ccccc3)(C)C | _21_ | 0.044 | | aromatic hydroxylation | C_24_H_31_N_3_O_2_ | 393.5218 |  |  |  |
| 23 | O=C(c2nn(c1ccccc12)CCCC(C)(C)C)NC(c3ccccc3O)(C)C | 22 | 0.036 | | aromatic hydroxylation | C_24_H_31_N_3_O_2_ | 393.5218 |  |  |  |
| 24 | O=C(c2nn(c1ccccc12)CC(O)CC(C)(C)C)NC(c3ccccc3)(C)C | 22 | 0.036 | | aliphatic hydroxylation | C_24_H_31_N_3_O_2_ | 393.5218 |  |  |  |
| 25 | [O-][n+]2c(c1ccccc1n2CCCC(C)(C)C)C(=O)NC(c3ccccc3)(C)C | 24 | 0.032 | | *N*-oxidation | C_24_H_31_N_3_O_2_ | 393.5218 |  |  |  |
| 26 | O=C(c2nn(c1ccccc12)/C=C/CC(C)(C)C)NC(c3ccccc3)(C)C | 25 | 0.020 | | alkyl dehydrogenation | C_24_H_29_N_3_O | 375.5066 |  |  |  |

**Possible mechanism for an “*N*-*O* exchange” in SCRA fragments**

The phenomenon of SCRAs and their metabolites exhibiting a fragment suggesting an “*N*-*O* exchange” is known from prior studies. This was discovered for MDMB-4en-PINACA, ADB-PINACA, 5F-ADB-PINACA and some of their metabolites lacking further explanation in the literature. (Erol Ozturk and Yeter, 2020; Gu et al., 2022; Carlier et al., 2017) For 5F-ADB (5F-MDMB-PINACA),it has been suggested that an amide cleavage followed by a nucleophilic attack of the nitrogen at position 2 of the indazole core by oxygen forms a seven-membered ring, which is subsequently degraded by the cleavage of amide and ester bonds (Richter et al., 2019). However, the contamination of the CAD gas with water residues, for example, cannot be completely ruled out. This could lead to a formal “*N-O* exchange” occurring within the ion source, explained by a nucleophilic attack of the oxygen atom of a water molecule at the indazole *N*2. The putative mechanism occurring in the HESI-ion source is illustrated in Fig. S5.


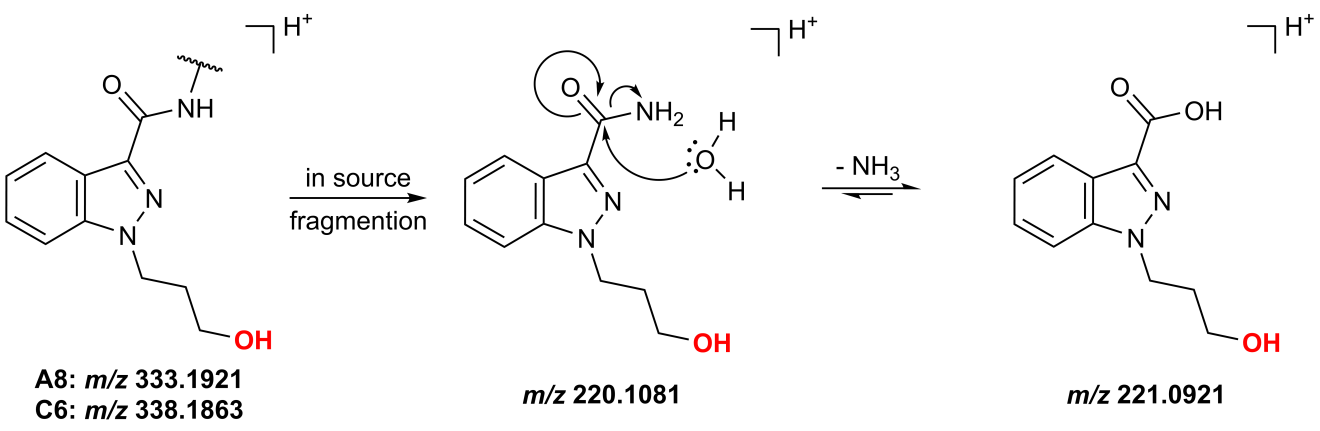


**Fig. S5** Possible ion source fragmentation and nucleophilic attack of the oxygen in a water molecule resulting in fragments seen for most metabolites and parent substances, here exemplified for metabolite A8 and C6

The MS/MS spectra of the expected indazole core-carboxamide-side chain and the corresponding ‘special’ fragment with the assumed ‘*N*-*O* exchange’ for the metabolites C6 and C8 can be found in Fig. S6. Whether this process is temperature or pH dependent requires further investigations.

**a**

**b**

^13^C

234.0870

1+

**235.0903**

1+

TS Std. Met 2 Cumyl-3TMS-PrINACA - 3TMS+COOH, 352 bbCID, ca. 40 ng_mL, 5 uL inj_1_4754.d: +MS

**234.0874**

1+

**235.0713**

1+

TS Std. Met 2 Cumyl-3TMS-PrINACA - 3TMS+COOH, 352 bbCID 15+-5eV, ca. 40 ng_mL, 5 uL inj_1_4754.d: +bbCID MS, **24.0-36.0eV**

0.0

0.5

1.0

1.5

6

x10

Intens.

0

1

2

3

4

5

5

x10

234.2

234.4

234.6

234.8

235.0

235.2

m/z

^13^C

^13^C

^13^C

^13^C

220.1080

1+

**221.1112**

1+

TS Std. Met 1 Cumyl-3TMS-PrINACA - 3TMS+OH, 338 bbCID ca. 40 ng_mL, 5 uL inj_1_4752.d: +MS

**220.1079**

1+

**221.0920**

1+

TS Std. Met 1 Cumyl-3TMS-PrINACA - 3TMS+OH, 338 bbCID ca. 40 ng_mL, 5 uL inj_1_4752.d: +bbCID MS, **24.0-36.0eV**

0

1

2

3

4

5

6

x10

Intens.

0.0

0.5

1.0

1.5

2.0

6

x10

220.2

220.4

220.6

220.8

221.0

221.2

m/z

**Fig. S6** MS and MS/MS (bbCID) spectra of fragments of metabolite C8 (a) and C6 (b). The 1-(2-carboxyethyl)-1*H*-indazole-3-carboxamidium (A, *m/z* 234.0873), the 1-(3-hydroxypropyl)-1*H*-indazole-3-carboxamidium (B, *m/z* 220.1081) and their corresponding ^13^C isotope fragments are present. In MS/MS spectra, the [1-(2-carboxyethyl)-1*H*-indazole-3-carbonyl]oxidanium (A, 235.0713) and the [1-(3-hydroxypropyl)-1*H*-indazole-3-carbonyl]oxidanium (B, 221.0921) exhibit the ion peak area of the expected fragments

**Case Report of a fatal intoxication involving ADMB-3TMS-PrINACA**

**Case History**

The deceased was found unresponsive in a locked bathroom after a social gathering; no drug paraphernalia were present at the scene, though a herbal mixture was located in the kitchen. The individual had a history of schizoaffective disorder, residual schizophrenia, generalized anxiety disorder, epilepsy, and dependence on multiple substances (cannabis, synthetic cannabinoids, stimulants, alcohol), and had been in long-term psychiatric and addiction treatment. According to the preliminary post-mortem report, acute heart failure could not be excluded.

**Sample preparation and LC-MS/MS method for screening for SCRAs**

For the detection of synthetic cannabinoids, blood samples and stomach content were processed as follows: For synthetic cannabinoid detection, 200 µL blood was fortified with internal standards, extracted using 10 M ammonium formate and 1 mL acetonitrile, then evaporated and reconstituted in mobile phase. Analysis was performed via LC–MS/MS using an Ultimate 3000RS UHPLC (Dionex) coupled to a QTRAP® 6500 (SCIEX) in positive ESI mode. Separation employed a Kinetex® C18 column (2.6 µm, 100 × 2.1 mm; Phenomenex) with a gradient per Huppertz et al. (Huppertz et al., 2014). A scheduled MRM method with two transitions per analyte and one per internal standard was applied, optimizing DP, EP, CE, and CXP. Calibration spanned 0.1 – 10 ng/mL. For ADMB-3TMS-PrINACA (retention time: 4.4 min; 389.2-> 259.1; 389.2-> 344.2) JWH-015-*D7* (335.1->155.1) was used as internal standard. For ADMB-3TMS-PrINACA *N*-propionic acid, the transitions 347.2-> 217.1; 347.2-> 145.0 (retention time: 2.35 min) were used.

Mobile phase A was 1% acetonitrile, 0.1% formic acid, and 2 mM ammonium formate in water; mobile phase B contained 0.1% formic acid and 2 mM ammonium formate in acetonitrile. Both were freshly prepared before analysis.

Blood and urine screening was performed using validated, accredited and updated LC-MS/MS methods including designer stimulants, hallucinogens, benzodiazepines, and opioids relevant to the European market. (Franz et al., 2017; Grapp et al., 2020; Koch et al., 2018)

**Quantitative results of drugs in the femoral vein blood and urine**

**Table S12** Quantitative results of drugs detected in femoral vein blood and urine of the deceased via external calibration. n.d.: not detected

| Substance | Femoral vein blood | Urine |
| --- | --- | --- |
| Synthetic cannabinoids |  |  |
| ADB-BUTINACA | ca. 0.25 ng/mL | n.d. |
| ADB-BUTINACA (*N*-3OH, hydrolysis product) | detected | detected |
| ADMB-3TMS-PrINACA | ca. 6.6 ng/mL | n.d. |
| ADMB-3TMS-PrINACA (*N*-propionic acid) | detected | detected |
| Cumyl-3TMS-PrINACA (*N*-propionic acid) | detected | detected |
| Metabolite of CH-PIATA (*N*-pentanoic acid) | detected | detected |
| Stimulants |  |  |
| Methamphetamine | ca. 120 ng/mL | > 50 ng/mL |
| Amphetamine | ca. 29 ng/mL | ca. 600 ng/mL |
| Ephedrine | n.d. | ca. 4.9 ng/mL |
| Norephedrine | n.d. | ca. 9.5 ng/mL |
| Pseudoephedrine | n.d. | ca. 2.7 ng/mL |
| Opioids |  |  |
| Fentanyl | 6.5 ng/mL | 19 ng/mL |
| Norfentanyl (Fentanyl metabolite) | 1.3 ng/mL | ca. 76 ng/mL* |
| 4-ANPP (Fentanyl metabolite) | 0.14 ng/mL | 0.29 ng/mL |
| Tilidine | 5.3 ng/mL | 16 ng/mL |
| Nortilidine (Tilidine metabolite) | 5.6 ng/mL | 31 ng/mL |
| Naloxon | n.d. | 23 ng/mL |
| Benzodiazepines |  |  |
| Diazepam | 20 ng/mL | 18 ng/mL |
| Nordazepam | 14 ng/mL | 170 ng/mL |
| Oxazepam | ca. 2.5 ng/mL | 210 ng/mL |
| Temazepam | ca. 1.7 ng/mL | 160 ng/mL |
| Hallucinogens | n.d. | n.d. |

In addition, doxepin (268 ng/mL), levomepromazine (91 ng/mL) and pregabaline 10.7 µg/mL could be detected in the femoral blood. Neither in blood nor in urine ethyl alcohol could be detected.

Only ADMB-3TMS-PrINACA was present in the herbal mixture found at the scene, analyzed by GC-MS.

Despite the presence of low concentrations of the highly potent SCRA ADB-BUTINACA in combination with high, potentially lethal concentrations of fentanyl, ADMB-3TMS-PrINACA may also have been the cause of death in combination with these substances. Even though he already had experience with the use of SCRAs. Due to the combined effects of especially fentanyl, diazepam and pregabalin on the central nervous system, the risk of dangerous sedation and life-threatening respiratory depression is increased.

Based on the results of the toxicological analyses, the cause of death was rated as mixed intoxication with multiple substance abused.

**References used in the Supplementary Information**

Carlier, J., Diao, X., Scheidweiler, K.B., Huestis, M.A., 2017. Distinguishing Intake of New Synthetic Cannabinoids ADB-PINACA and 5F-ADB-PINACA with Human Hepatocyte Metabolites and High-Resolution Mass Spectrometry. Clinical Chemistry 63, 1008–1021. https://doi.org/10.1373/clinchem.2016.267575

Erol Ozturk, Y., Yeter, O., 2020. In Vitro Phase I Metabolism of the Recently Emerged Synthetic MDMB-4en-PINACA and Its Detection in Human Urine Samples. Journal of Analytical Toxicology 44, 976–984. https://doi.org/10.1093/jat/bkaa017

Franz, F., Angerer, V., Jechle, H., Pegoro, M., Ertl, H., Weinfurtner, G., Janele, D., Schlögl, C., Friedl, M., Gerl, S., Mielke, R., Zehnle, R., Wagner, M., Moosmann, B., Auwärter, V., 2017. Immunoassay screening in urine for synthetic cannabinoids – an evaluation of the diagnostic efficiency. Clinical Chemistry and Laboratory Medicine (CCLM) 55, 1375–1384. https://doi.org/10.1515/cclm-2016-0831

Grapp, M., Kaufmann, C., Schwelm, H.M., Neukamm, M.A., Blaschke, S., Eidizadeh, A., 2020. Intoxication cases associated with the novel designer drug 3′,4′-methylenedioxy-α-pyrrolidinohexanophenone and studies on its human metabolism using high-resolution mass spectrometry. Drug Testing and Analysis 12, 1320–1335. https://doi.org/10.1002/dta.2869

Gu, K., Qin, S., Zhang, Y., Zhang, W., Xin, G., Shi, B., Wang, J., Wang, Y., Lu, J., 2022. Metabolic profiles and screening tactics for MDMB-4en-PINACA in human urine and serum samples. Journal of Pharmaceutical and Biomedical Analysis 220, 114985. https://doi.org/10.1016/j.jpba.2022.114985

Huppertz, L.M., Kneisel, S., Auwärter, V., Kempf, J., 2014. A comprehensive library-based, automated screening procedure for 46 synthetic cannabinoids in serum employing liquid chromatography-quadrupole ion trap mass spectrometry with high-temperature electrospray ionization. Journal of Mass Spectrometry 49, 117–127. https://doi.org/10.1002/jms.3328

Koch, K., Auwärter, V., Hermanns-Clausen, M., Wilde, M., Neukamm, M.A., 2018. Mixed intoxication by the synthetic opioid U-47700 and the benzodiazepine flubromazepam with lethal outcome: Pharmacokinetic data. Drug Testing and Analysis 10, 1336–1341. https://doi.org/10.1002/dta.2391

Richter, L.H.J., Maurer, H.H., Meyer, M.R., 2019. Metabolic fate of the new synthetic cannabinoid 7’N-5F-ADB in rat, human, and pooled human S9 studied by means of hyphenated high-resolution mass spectrometry. Drug Testing and Analysis 11, 305–317. https://doi.org/10.1002/dta.2493
